# Variational kinetics: elementary reaction kinetics via conic optimisation

**Published:** 2026-08-03

**Authors:** Ronan M.T. Fleming, Ines Thiele

**Affiliations:** 1 Digital Metabolic Twin Centre, University of Galway, University Road, Galway, Ireland; 2 School of Medicine,University of Galway, University Road, Galway, Ireland; 3 Institute for Clinical Trials,University of Galway, University Road, Galway, Ireland; 4 School of Microbiology, University of Galway, University Road, Galway, Ireland; 5 Ryan Institute,University of Galway, University Road, Galway, Ireland

**Keywords:** Variational kinetics, exponential cone, conic optimisation, elementary reaction kinetics, genome-scale metabolic model

## Abstract

Genome-scale modelling methods primarily predict reaction fluxes, whereas established high throughput experimental technologies primarily measure molecular species concentrations. This apparently paradoxical situation has arisen because implementing the nonlinear constraints that represent reaction kinetic rate equations is challenging without resorting to convenient yet inaccurate approximations or to expansions that are valid only near a reference state. We present a mathematically and computationally tractable solution to this problem. First, we introduce a mathematical reformulation of established knowledge of metabolic reactions and reaction kinetics in matrix–vector notation. We then present *variational kinetics*, a novel approach that satisfies steady state reaction kinetics at genome scale by exponential conic optimisation. The nonlinear rate law constraints are relaxed to exponential cones, which renders the feasible set convex, and satisfaction of elementary kinetics is recovered by minimising a strictly concave merit function over that set, which attains zero if, and only if, every rate law holds. We establish that a particular sequence of conic optimisation problems converges to a stationary point of this merit function, and that every such stationary point is a steady state satisfying elementary kinetics. Moiety conservation, thermodynamic constraints on elementary kinetic parameters, regularised steady states and linear optimisation of external reaction rates are each accommodated within the same conic formulation. We demonstrate the approach computationally on a genome-scale metabolic model.

## Graphical Abstract

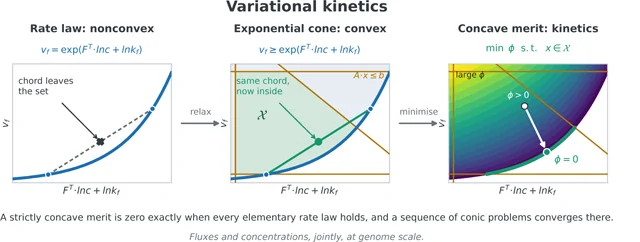

## 1 Introduction

Metabolism is the network of enzyme-catalysed reactions through which cells extract energy and synthesise the molecules required for life. Reconstructed at the scale of an entire genome, this network underpins genome-scale models of metabolism, which have become standard tools for interpreting biochemical, genetic and clinical data and for engineering cellular chemistry. A metabolic network admits two complementary quantitative descriptions: the rates at which its reactions proceed, that is their fluxes, and the concentrations of the metabolites they interconvert [1]. A model that predicted both, consistently, would let measurements of one be interpreted in terms of the other — but the two descriptions have proven far easier to obtain apart than together.

Constraint-based methods, such as flux balance analysis [2], predict fluxes from network stoichiometry together with an optimisation principle. They scale readily to genome-scale networks, but deliberately omit reaction kinetics and therefore cannot predict metabolite concentrations. High-throughput technologies, by contrast, increasingly measure concentrations rather than fluxes. The quantities that are easiest to predict are thus not the quantities that are easiest to measure. Perfectly closing this gap requires introduction of reactionkinetic rate laws that couple concentrations to fluxes in a thermodynamically consistent manner. However, the corresponding constraints are nonlinear, the resulting feasible set is non-convex [3], and therefore satisfying them at genome-scale has proven both mathematically and computationally demanding.

There are many modelling methods that introduce various aspects of reaction-kinetics, thermodynamic consistency, or both, into genome-scale models. We do not attempt an exhaustive reference list, rather we refer to certain approaches as examples of the challenges associated with different classes of methods. Thermodynamic constraints on the direction of reactions can be applied by introduction of thermochemical constraints [4], but this relies on extrapolation from measured thermochemical data [5] and sufficient metabolomic data. Thermodynamic constraints on combinations of reaction directions can be efficiently satisfied at genome-scale using linear optimisation [6] and combined with constraints relating the ratio of unidirectional fluxes to thermodynamic driving force [7] using non-linear yet convex optimisation [8], which can also be biased using available omics data [9]. However, these methods omit constraints relating molecular species abundance (enzymes, metabolites) to absolute reaction rates.

A complementary line of work takes a flux distribution as given and predicts the accompanying metabolite concentrations by convex optimisation: the max-min driving force method [10] selects concentrations that maximise the smallest thermodynamic driving force along a pathway, while enzyme cost minimisation [11] selects them to minimise the total enzyme demand implied by the rate laws. These formulations are convex and scale well, but they presuppose a known flux distribution and enforce only thermodynamic feasibility, or an enzyme-cost optimum, rather than the elementary rate law itself, so they determine concentrations for a given flux rather than fluxes and concentrations together. Provided that kinetic constraints and kinetic parameters are formulated in a thermodynamically consistent manner [12], introduction of reaction-kinetic rate laws obviates the need to apply separate thermodynamic constraints. As introduction of reaction-kinetic rate laws is challenging, a variety of different methods have instead attempted to represent various aspects of reaction kinetics using approximations to rate laws. Reaction rates can be bounded from above using the product of $k_{cat}$ times the concentration of the catalysing enzyme and this, together with an upper bound for the available enzyme pool, results in a scalable linear approximation to reaction kinetics [13, 14, 15] but does not take into account non-linear kinetic effects such as saturation, thermodynamic driving force, or regulation. Rate laws may be approximated by a log-linear expansion about a reference state, in which the logarithm of a reaction rate is a linear function of the logarithms of the reactant concentrations [16]. The closely related linear-logarithmic (lin-log) kinetics [17] instead make the rate itself, scaled by enzyme level, a linear function of the logarithmic concentrations, taking the elasticities of metabolic control analysis as its parameters. However, these approximations share a common compromise as expansions are built about a fixed reference state and lose accuracy away from it (cf. Supplementary Figure 4). An alternative stepwise approach is to represent reaction-kinetic rate laws but anchor a model ensemble to reference steady states, e.g., obtained from fluxomic data across many strains, then prune it using perturbation data [18, 19, 20], but this requires sufficient reference data at genome-scale, and the retained ensemble is not uniquely identified, so its predictions remain sensitive to the choice of reference state and to the sampling and pruning procedure.

Reaction kinetics rate laws, and various approximations thereof, can be added to genome-scale kinetic models then the feasible set of fluxes, concentrations and parameters can be sampled in a manner consistent with experimental data [21, 22], however the feasible set is non-linear and non-convex so there are no guarantees that the numerical sample distribution will match the theoretically desired distribution. Alternatively, one can embed nonlinear rate laws directly into a constraint-based model and predict network states using mixed-integer nonlinear optimisation algorithms that exploit the mathematical properties particular to systems of reaction rate laws [23], however reliable convergence is not guaranteed as the network grows. Mass-action stoichiometric simulation builds dynamic models by mapping measured concentrations and fluxes onto the network [24, 25], but it requires simultaneous measurements of concentrations and fluxes to fix the model, and its mass-action form neglects enzyme saturation and allosteric regulation, so it is accurate only near the state at which it was parameterised. A common tension runs through these approaches: one must either approximate the kinetics and forfeit biochemical fidelity, depend on a pre-specified reference state and extensive parameterisation and forfeit uniqueness and identifiability, or confront a nonconvex optimisation that does not reliably scale. What remains missing is a formulation of steady-state elementary reaction kinetics that is at once mathematically exact, independent of any reference state, and expressed as a tractable optimisation that determines reaction fluxes and metabolite concentrations together.

Here we present variational kinetics, an approach that reformulates steady-state elementary reaction kinetics as an optimisation problem and solves it at genome scale through a sequence of exponential conic optimisation problems. There is prior reason to expect such a reformulation to be attainable: the thermodynamic and kinetic relationships that govern an elementary reaction are log-linear in the chemical potentials of its species, and the exponential cone captures exactly this log-linear structure, so it was plausible in advance that elementary kinetics could be cast as conic optimisation without approximation. We first restate established results on metabolic reactions and reaction kinetics in a consistent matrix-vector notation; we then develop the variational kinetics formulation together with the numerical method used to solve it, and characterise the conditions under which the iterative scheme converges to a steady state; finally we illustrate the approach on a genome-scale model of dopaminergic neuronal metabolism. Our aim throughout is to answer a single question: can thermodynamically and kinetically consistent reaction fluxes and metabolite concentrations be predicted at genome scale from network structure and kinetic parameters alone, without approximate rate laws and without a pre-specified reference state?

## 2 Notation

Throughout , $R,R^{n}$, and $R^{m\times n}$ denote the field of real numbers, the vector space of n-tuples of real numbers, and the space of m×n matrices with entries in R, respectively. Similarly, $Z,Z^{n},Z^{m\times n}$ stand for integer numbers, the vector space of n-tuples of integer number, and the space of matrices with entries in Z, respectively. $R_{\geq 0}^{n}$ and $R_{>0}^{n}$ denote non-negative real n-tuples and positive real n-tuples in $R^{n}$, respectively, and $Z_{\geq 0}^{n}$ and $Z_{>0}^{n}$ denote non-negative integer n-tuples and positive integer n-tuples in $Z^{n}$, respectively. We use Householder notation, that is a matrix is denoted by uppercase Roman, such as $A\in R^{m\times n}$. $A_{i}$ and $A_{:,j}$ denote the $i^{\text{th}}$ row and the $j^{\text{th}}$ column of A, respectively, where i∈1,…,m and j∈1,…,n. Note that subscript indexes are lower case Roman letters set in normal font like i, rather than italic i. $A^{T}$ denotes the transpose of a matrix A. 1 denotes a vector of all ones and I denote an identity matrix, with dimensions appropriate to the circumstance. A calligraphic, uppercase, roman letter, e.g., 𝒜, denotes a set, multiset or sequence, with {⋅,⋅} denoting an unordered pair, (⋅,⋅) denoting an ordered pair and (⋅,…,⋅) denoting a sequence. Let |𝒜| denote the cardinality of the set 𝒜.The dot product of x and y is denoted by $x^{T}\cdot y$, the Hadamard product (element-wise product) is denoted by x⊙y, the Hadamard divisor (element-wise division) is denoted by x🚫y. Such products and divisors of vectors require both vectors to have compatible dimensions. Where x is a vector $x^{-1}=1🚫x$ both denote a componentwise inverse. [⋅,⋅] denotes horizontal concatenation and $\left[\begin{array}{l} \cdot \\ \cdot \end{array}\right]$ or [⋅;⋅] denote vertical concatenation. Also, exp(x) of a vector x means componentwise exponential. Where a diagonal matrix formed from a vector is required it is written diag(⋅), while the Hadamard product is used wherever both operands are vectors of the same dimension. ∇ is the gradient of a scalar valued function, or, for a vector valued function, the matrix whose columns are the gradients of the components. The expression f(a∣b) means that the vector valued function f has a vector variable argument a, given a vector of parameters b. Let

$$sign(x):=\left\{\begin{array}{ll} 1 & \text{if}\;x>0,\\ -1 & \text{if}\;x<0,\\ 0 & \text{if}\;x=0, \end{array}\right.$$

and componentwise where x is a vector.

A disadvantage of reformulating nonlinear mathematical models in terms of conic optimisation is an expansion in the number of variables. With expansion of terms beyond the 26 letters in the Roman alphabet, one is forced to compromise on established notation guidelines in a manner that maintains a reasonable correspondence between symbols in a scientific paper and the corresponding symbols in computer programming code that are supposed to have the same meaning. The exponential or natural logarithm of a vector is meant componentwise and exp(ln(0)):=0. For example, let x denote a vector, then ln(x) denotes the componentwise natural logarithm of that vector and lnx denotes a variable that is envisaged to equal ln(x) at the optimum of a conic optimisation problem, within tolerances specified by parameters input into a numerical optimisation solver. This approach enables transparency in representation of correspondence between related variables, avoids premature exhaustion of the Roman alphabet, while marginally extending beyond Householder notation guidelines.

The following exceptions to the conventions above are retained, because each is well established in its own literature, named after a person, or needed to avoid a typographic ambiguity.

- ℓ denotes the vector of moiety concentrations. A lower case script ell is used in place of l so that it cannot be confused with the digit one.
- 𝓡,𝒯 and 𝒫 denote the gas constant, temperature and pressure. These are scalars, not sets, but the calligraphic forms are conventional in chemical thermodynamics. Note that 𝓡(⋅) also denotes the range of a matrix, which is a set; the two are distinguished by the presence of an argument.
- 𝓛 denotes a Lagrangian, after Lagrange. It is a function rather than a set.
- $V_{\text{max}}$ and $K_{M}$ denote the limiting rate and the Michaelis constant of a Michaelis-Menten rate law. These are scalars in upper case Roman, which is standard in enzyme kinetics.
- Units of measurement are set in upright type, that is K for kelvin, atm for atmosphere, mol for mole and L for litre.
- A symbol that carries one established meaning in the biochemical literature and a different established meaning in the optimisation literature is not disambiguated here. For example F denotes the forward stoichiometric matrix in the sections on reaction kinetics and the matrix of the affine conic constraint in the sections on conic optimisation, each being standard in its own field. The section in which a symbol appears determines which meaning is intended.

## 3 Mathematical formulation of reaction kinetics

### 3.1 Reaction stoichiometry

Consider a biochemical network with m molecular species and n reactions. Henceforth, species means molecular species. Typically, though not always m<n. We assume that all net reactions are *reversible* and that each can be represented by a pair of unidirectional, *forward* and *reverse*, reactions. With respect to the forward direction, let the relative quantity, or *stoichiometry*, of species i participating as a substrate or catalyst in a *forward reaction* j, be a whole number entry $F_{i,j}>0$, in a *forward stoichiometric matrix* $F\in Z^{m\times n}$. Likewise, with respect to the reverse direction, let the stoichiometry of species i participating as a substrate or catalyst in *reverse reaction* j, be an entry $R_{i,j}>0$, in a *reverse stoichiometric matrix* $R\in Z^{m\times n}$. Then, $N\in Z^{m\times n}:=R-F$ is a (net) stoichiometric matrix, where $N_{i,j}$ is the number of instances of molecule i that are consumed (negative) or produced (positive) in reaction j. We assume that each column of N corresponds a reaction where mass is conserved.

### 3.2 Elementary reaction kinetics

Elementary reaction kinetics refers to the study of the individual, simple steps that occur during a chemical reaction at the molecular level. Each elementary reaction represents a single molecular event, such as the breaking or formation of a chemical bond. An elementary reaction is one for which no reaction intermediates have been detected or need to be postulated in order to describe the chemical reaction on a molecular scale. Elementary reactions provide a detailed, step-by-step description of how a substrate interacts with an enzyme at the molecular level. Let $v_{f}\in R_{>0}^{n}$ and $v_{r}\in R_{>0}^{n}$ denote forward and reverse elementary reaction rates, both of which are a function of species concentrations $c\in R_{>0}^{m}$ as well as forward and reverse elementary kinetic parameters, denoted $k_{f}\in R_{>0}^{n}$ and $k_{r}\in R_{>0}^{n}$ respectively. Any reaction rate law assumes that reaction rate is a function of concentrations and kinetic parameters, so let the net reaction rate be

$$v_{net}\left(c|k_{f},k_{r}\right):=v_{f}\left(c|k_{f}\right)-v_{r}\left(c|k_{r}\right).$$

Henceforth, we assume that the rate of an elementary reaction is proportional to the product of the concentrations of each of substrate (or catalyst), each to the power of their respective stoichiometry in the reaction. It follows that *elementary kinetics* for the forward and reverse reaction rates of reaction j are given by

$$\begin{array}{rr} v_{fj}\left(c|k_{fj}\right) & :=k_{fj}\prod c_{i}^{F_{i,j}},\\ v_{rj}\left(c|k_{rj}\right) & :=k_{rj}\prod c_{i}^{R_{i,j}}. \end{array}$$

Using matrix vector notation and elementary logarithmic and exponential identities, we formulate elementary kinetics for the forward and reverse reaction rate vectors as

(1) $$v_{f}\left(c|k_{f}\right)=exp\left(ln\left(k_{f}\right)+F^{T}\cdot ln(c)\right),$$

(2) $$v_{r}\left(c|k_{r}\right)=exp\left(ln\left(k_{r}\right)+R^{T}\cdot ln(c)\right).$$

The exponential or natural logarithm of a vector is meant componentwise ^1^. A set of concentrations, kinetic parameters and rates that satisfy Eq. (1) and Eq. (2) are said to be *kinetically feasible*. Taking the logarithm of both sides of Eqs. (1) and (2) we have

(3) $$ln\left(v_{f}\right)=ln\left(k_{f}\right)+F^{T}\cdot ln(c),$$

(4) $$ln\left(v_{r}\right)=ln\left(k_{r}\right)+R^{T}\cdot ln(c).$$

Assuming elementary reaction kinetics, net rate is

(5) $$v_{net}\left(c|k_{f},k_{r}\right)=v_{f}\left(c|k_{f}\right)-v_{r}\left(c|k_{r}\right)=exp\left(ln\left(k_{f}\right)+F^{T}\cdot ln(c)\right)-exp\left(ln\left(k_{r}\right)+R^{T}\cdot ln(c)\right).$$

Note that net rate is a function of species concentration given kinetic parameters. That is, for now we assume we are given kinetic parameters and are only interested in modelling concentrations and rates. Of course, in reality the situation is more complicated as, at best, one has experimental estimates of kinetic parameters.

### 3.3 Steady state

With respect to time t, the rate of change of species concentrations is given by the dot product of net reaction stoichiometry and net reaction rate

$$\dot{c}:=\frac{dc}{dt}=N\cdot v_{net}\left(c|k_{f},k_{r}\right),$$

which is an ordinary differential equation. When ${\dot{c}}_{i}=0$ the rate of production equals the rate of consumption of species i, that is, species i is at a steady state. When ${\dot{c}}_{i}>0$ the rate of production is greater than the rate of consumption of species i and when ${\dot{c}}_{i}<0$ the rate of production is less than the rate of consumption of species i. Assuming elementary reaction kinetics, Eq. 5, we have

(6) $$\dot{c}=N\cdot \left(exp\left(ln\left(k_{f}\right)+F^{T}\cdot ln(c)\right)-exp\left(ln\left(k_{r}\right)+R^{T}\cdot ln(c)\right)\right),\;\;=[N,-N]\cdot exp\left(ln\left(\left[\begin{array}{c} k_{f}\\ k_{r} \end{array}\right]\right)+[F,R{]}^{T}\cdot ln(c)\right)\;\;=([R,F]-[F,R])\cdot exp\left(ln\left(\left[\begin{array}{c} k_{f}\\ k_{r} \end{array}\right]\right)+[F,R{]}^{T}\cdot ln(c)\right)$$

where the latter are obtained by gathering related terms and substituting N=R-F to present, in matrix vector format, the fundamental equation representing evolution of concentration with respect to time according to elementary reaction kinetics.

### 3.4 Mass balance

Let $B\in Z^{m\times k}$ be a stoichiometric matrix, where each column corresponds to an *external reaction*, which is a modelling construct used to represent the exchange of mass between a biochemical network and its environment. Let $w\in R^{k}$ denote net exchange reaction rate. Assuming *mass balance*, the rate of change of species concentrations is equal to the net rate of exchange with the environment. By convention, this is expressed as

$$\dot{c}=-B\cdot w$$

or equivalently

(7) $$\begin{array}{rr} N\cdot \left(v_{f}\left(c|k_{f}\right)-v_{r}\left(c|k_{r}\right)\right)+B\cdot w & =0\\ N\cdot v_{net}\left(c|k_{f},k_{r}\right)+B\cdot w & =0. \end{array}$$

Note that with this convention, if $w_{j}<0$ and $B_{i,j}<0$, then this means that species i is input from the environment, while if $w_{j}>0$ and $B_{i,j}<0$, then this means that species i is output to the environment. Eq. (7) means that production + input = consumption + output for every species. Henceforth, for brevity, we use the term steady state to mean either a strict steady state, for molecular species not exchanged across the boundary of the system, or for species that are exchanged across the boundary (strictly mass balance). For each metabolite, whether this means strictly steady state or mass balance, is evident from the context.

### 3.5 Moiety conservation

Every genome-scale stoichiometric matrix has linearly dependent rows, that is r:=rank(N)<m. Let $L\in Z^{(m-r)\times m}$ denote a left nullspace basis for N, that is L⋅N=0. The number of linearly dependent rows, or row rank deficiency, is m-r. Each linearly dependent row corresponds to a conserved moiety, which is a chemical substructure that remains invariant with respect to the chemical transformations in a given network [27]. This *moiety conservation* imposes constraints on the relationship between an initial species concentration vector at time zero $c_{0}\in R_{\geq 0}^{m}$ and all subsequent species concentrations at time t, denoted $c:=c(t)\in R_{\geq 0}^{m}$, since

(8) $$\begin{array}{ll} \int_{0}^{t}N\cdot v_{net}(c)dt & =c-c_{0}\\ 0 & =L\cdot \left(c-c_{0}\right). \end{array}$$

Given a stoichiometric matrix and molecular structures for each species, using atom mapping and graph theoretical algorithms, it is possible to compute a *non-negative* left nullspace basis $L\in Z_{\geq 0}^{(m-r)\times m}$ where $L_{i,j}$ is equal to the number of instances of conserved moiety i in metabolite j [27, 28]. Hence, Eq. (8) is referred to as a moiety conservation constraint [29] and L a moiety incidence matrix [28].

### 3.6 Mass balance subject to elementary reaction kinetics

If one assumes that a system is at a steady state and that all reactions must satisfy elementary reaction kinetics, then this requires the solution to the following system of equations

(9) $$N\cdot \left(exp\left(ln\left(k_{f}\right)+F^{T}\cdot ln(c)\right)-exp\left(ln\left(k_{r}\right)+R^{T}\cdot ln(c)\right)\right)+B\cdot w=0.$$

Due to the exponential and logarithmic terms, it is clear that Eq. (9) is non-linear. Furthermore, the set of all solutions to Eq. (9) is known not to be *convex*, i.e., if one draws a straight line between two points in that set, then there may be points along that line which are not in that set. This non-convexity makes it challenging to find solutions to Eq. (9), e.g., for the high dimensional models that typically arise from genome-scale metabolic models. Furthermore, due to various reasons, the uncertainty in our knowledge of many kinetic parameters is large, so in a modelling context $k_{f}$ and $k_{r}$ are not fixed parameters, but rather variables that may be penalised from their deviation to a subset of known experimentally measured kinetic parameters.

### 3.7 Moiety conserved elementary reaction kinetics

Biochemical networks are open systems that are forced away from equilibrium by exchange of mass with their environment. Typically this is modelled with a set of exchange reactions, each of which is a modelling construct (pseudoreaction) that does not conserve mass and either uptakes species from the environment or secretes species to the environment. However, to the best of the authors’ knowledge, there exist no conditions established to guarantee that such a system admits a kinetic steady state. Previously, we established an approach to force a biochemical network away from equilibrium such that a non-equilibrium steady state still exists [30]. A corresponding existence theorem is proven below in terms of ordinary differential equation theory.

#### Theorem 1.

*Let the dynamical equation for mass conserved elementary kinetics be*

(10) $$\dot{c}=(R-F)\cdot \left(k_{f}⊙exp\left(F^{T}\cdot ln(c)\right)-k_{r}⊙exp\left(R^{T}\cdot ln(c)\right)\right),$$

*where* $c=c(t)\in R_{>0}^{m}$ *is a species concentrations at time* $t>0,\dot{c}\in R^{m}$ *is the time derivative of concentrations and* $k_{f},k_{r}\in R_{\geq 0}^{n}$ *are non-negative forward and reverse kinetic parameters and* $F,R\in Z_{\geq 0}^{m\times n}$ *are forward and reverse stoichiometric matrices. Assuming a finite and strictly positive initial concentration* $c(0)\in R_{>0}^{m}$, *and the existence of at least one strictly positive vector* $\ell \in R_{>0}^{m}$, *such that*

$$(R-F{)}^{T}\cdot \ell =0.$$

*then there exists at least one finite and non-negative steady state concentration* $c^{⋆}\in R_{>0}^{m}$, *such that* $\dot{c}=0$.

*Proof.* Consider10 an autonomous ordinary differential equation

(11) $${\dot{c}}_{i}=\sum_{j=1}^{n}\left(R_{i,j}-F_{i,j}\right)\left(k_{fj}\prod_{i=1}^{m}c_{i}^{F_{i,j}}-k_{rj}\prod_{i=1}^{m}c_{i}^{R_{i,j}}\right),\;c(0)>0.$$

By assumption, the system satisfies concentration non-negativity: if $c_{i}(t)=0$ for some i, then ${\dot{c}}_{i}(t)\geq 0$. Hence the nonnegative orthant $R_{\geq 0}^{m}$ is forward invariant. Multiplying(10) by $\ell \in R_{>0}^{m}$ yields

$$\ell^{T}\cdot \dot{c}=\ell^{T}\cdot (R-F)\cdot (\cdot )=0,$$

and therefore

(12) $$\ell^{T}\cdot c(t)=\ell^{T}\cdot c(0)\;\forall t\geq 0.$$

Define

$$\Omega :=\left\{c\in R_{\geq 0}^{m}:\ell^{T}\cdot c=\ell^{T}\cdot c(0)\right\}.$$

Because ℓ≻0, each component satisfies

$$0\leq c_{i}\leq \frac{\ell^{T}\cdot c(0)}{\ell_{i}},$$

so Ω is nonempty, closed, bounded, and convex, hence compact. By(12) and forward invariance of $R_{\geq 0}^{m},\Omega$ is invariant under the dynamics.

Write the right-hand side of(11) as the vector field $g(c):=(R-F)\cdot \left(k_{f}⊙exp\left(F^{T}\cdot ln(c)\right)-k_{r}⊙exp\left(R^{T}\cdot ln(c)\right)\right)$. The reaction rates, written componentwise as monomials in(11), extend continuously to $R_{\geq 0}^{m}$, so g is continuous on Ω. Since these monomials are smooth on $R_{>0}^{m}$, solutions are unique, and for each τ>0 the time-τ flow map

$$\Phi_{\tau }:\Omega \to \Omega ,\;\Phi_{\tau }\left(c_{0}\right)=c(\tau ),$$

is a well-defined continuous self-map of Ω.

Fix a sequence $\tau_{n}↓0$. For each $n,\Phi_{\tau_{n}}$ maps the compact convex set Ω continuously into itself, so Brouwer’s fixed point theorem yields $c_{n}\in \Omega$ with

(13) $$\Phi_{\tau_{n}}\left(c_{n}\right)=c_{n}.$$

A point fixed by $\Phi_{\tau_{n}}$ has a $\tau_{n}$-periodic orbit and is not yet a steady state, since any nonconstant orbit whose period divides $\tau_{n}$ is also fixed by $\Phi_{\tau_{n}}$. To extract an equilibrium, use compactness of Ω to pass to a subsequence with $c_{n}\to c^{⋆}\in \Omega$. In integral form,

$$0=\Phi_{\tau_{n}}\left(c_{n}\right)-c_{n}=\int_{0}^{\tau_{n}}g\left(\Phi_{s}\left(c_{n}\right)\right)ds,$$

so dividing by $\tau_{n}$ gives the time average

(14) $$\frac{1}{\tau_{n}}\int_{0}^{\tau_{n}}g\left(\Phi_{s}\left(c_{n}\right)\right)ds=0.$$

**Figure 1:**
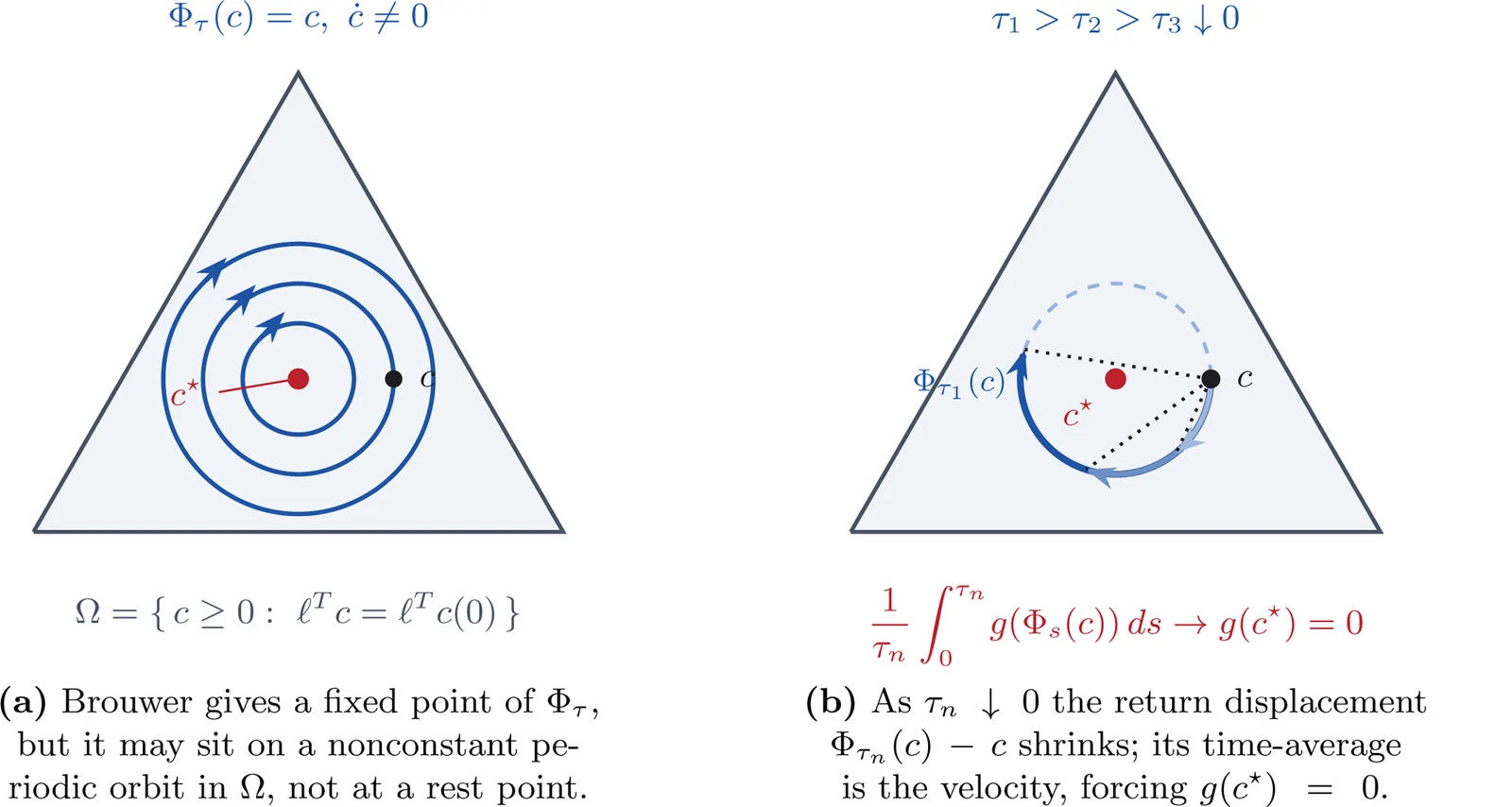
Illustration of a key part of the proof of Theorem 1 (a) A single-time statement $\Phi_{\tau }\left(c^{⋆}\right)=c^{⋆}$ for one fixed τ>0 is not by itself enough to conclude ${\dot{c}}^{⋆}=0$. It asserts only that the orbit through $c^{⋆}$ returns to $c^{⋆}$ after time τ, i.e. that the orbit is τ-periodic. A nonconstant periodic orbit whose period divides τ satisfies the same fixed-point equation while having $\dot{c}\neq 0$ throughout, so a fixed point of $\Phi_{\tau }$ need not be an equilibrium. (b) Letting $\tau_{n}↓0$ removes this gap: the time-averaged velocity over $\left[0,\tau_{n}\right]$ vanishes at each $c_{n}$, and in the limit this average becomes the instantaneous velocity $g\left(c^{⋆}\right)$, forcing $g\left(c^{⋆}\right)=0$.

Because g is bounded on the compact set Ω, for every $s\in \left[0,\tau_{n}\right]$

$$\left\|\Phi_{s}\left(c_{n}\right)-c^{⋆}\right\|\leq \tau_{n}\;\underset{c\in \Omega }{sup}\|g(c)\|+\left\|c_{n}-c^{⋆}\right\|⟶0$$

uniformly in s as n→∞. Since g is uniformly continuous on Ω, the integrand converges uniformly to $g\left(c^{⋆}\right)$, and hence the average in(14) converges to $g\left(c^{⋆}\right)$. Therefore

$$g\left(c^{⋆}\right)=0,\;\text{i.e.}\;{\dot{c}}^{⋆}=0,$$

so $c^{⋆}$ is a steady state of (11), with $c^{⋆}⪰0$ and finite (cf.. Figure 1 for an illustration of this argument). This completes the proof. □

### 3.8 Thermodynamically feasible kinetic parameters

We assume chemical potential $u\in R^{m}$ is

$$u:=u^{\circ }+𝓡𝒯\;ln\;(c)$$

where $u^{\circ }\in R^{m}$ is a vector of standard chemical potentials. This is a simplification of chemical potential in biochemical thermodynamics, but the mathematical form is the same for a variety of more sophisticated formulations (cf Appendix E) . The change in chemical potential for a system of biochemical reactions is denoted

$$\Delta u:=N^{T}\cdot u.$$

At thermodynamic equilibrium for all reactions, the sum of substrate chemical potentials equals the sum of product chemical potentials for each reaction, so the change in chemical potential for all reactions is zero and therefore

$$N^{T}\cdot u=0\Leftrightarrow \frac{-N^{T}\cdot u^{\circ }}{𝓡𝒯}=N^{T}\cdot ln\left(c_{eq}\right),$$

where $c_{eq}\in R^{m}$ is a vector of species concentrations at equilibrium. At thermodynamic equilibrium, without a driving force, the forward and reverse elementary reaction rates must be equal *(detailed balance* [26]). This requirement means that elementary kinetic parameters are thermodynamically constrained, as the following sequence of algebraic steps show

(15) $$\begin{array}{rr} v_{f}\left(c_{eq}|k_{f}\right)-v_{r}\left(c_{eq}|k_{r}\right) & =0\\ v_{f}\left(c_{eq}|k_{f}\right) & =v_{r}\left(c_{eq}|k_{r}\right)\\ exp\left(ln\left(k_{f}\right)+F^{T}\cdot ln\left(c_{eq}\right)\right) & =exp\left(ln\left(k_{r}\right)+R^{T}\cdot ln\left(c_{eq}\right)\right)\\ ln\left(k_{f}\right)+F^{T}\cdot ln\left(c_{eq}\right) & =ln\left(k_{r}\right)+R^{T}\cdot ln\left(c_{eq}\right)\\ ln\left(k_{f}\right)-ln\left(k_{r}\right) & =R^{T}\cdot ln\left(c_{eq}\right)-F^{T}\cdot ln\left(c_{eq}\right)\\ ln\left(\frac{k_{f}}{k_{r}}\right) & =(-F+R{)}^{T}\cdot ln\left(c_{eq}\right)\\ ln\left(\frac{k_{f}}{k_{r}}\right) & =N^{T}\cdot ln\left(c_{eq}\right)\\ ln\left(\frac{k_{f}}{k_{r}}\right) & =-N^{T}\cdot \frac{u^{\circ }}{𝓡𝒯} \end{array}$$

Elementary reaction kinetics (2) coupled with the thermodynamic constraints in Eq. (15) is referred to as *mass action kinetics*, represented by the following pair of equation systems

(16) $$\begin{array}{rr} \dot{c} & =N\cdot \left(exp\left(ln\left(k_{f}\right)+F^{T}\cdot ln(c)\right)-exp\left(ln\left(k_{r}\right)+R^{T}\cdot ln(c)\right)\right),\\ ln\left(\frac{k_{f}}{k_{r}}\right) & =-N^{T}\cdot \frac{u^{\circ }}{𝓡𝒯}. \end{array}$$

### 3.9 Thermodynamic constraints on reaction rates

Thermodynamic constraints on kinetic reactions imply thermodynamic constraints on reaction rates. To observe this implication, start with the definition of elementary reaction kinetics for the forward and reverse rates in Eq. (1) and (2) and let $v_{f}=v_{f}\left(c|k_{f}\right)$ and $v_{r}=v_{r}\left(c|k_{r}\right)$, so we have

$$\frac{v_{f}}{v_{r}}=\frac{exp\left(ln\left(k_{f}\right)+F^{T}\cdot ln(c)\right)}{exp\left(ln\left(k_{r}\right)+R^{T}\cdot ln(c)\right)}\;\;=exp\left(ln\left(k_{f}\right)-ln\left(k_{r}\right)+F^{T}\cdot ln(c)-R^{T}\cdot ln(c)\right)\;\;=exp\left(ln\left(k_{f}\right)-ln\left(k_{r}\right)\right)⊙exp\left(F^{T}\cdot ln(c)-R^{T}\cdot ln(c)\right)\;\;=exp\left(ln\left(k_{f}\right)-ln\left(k_{r}\right)\right)⊙exp\left(-N^{T}\cdot ln(c)\right)\;\;=\left(\frac{k_{f}}{k_{r}}\right)⊙exp\left(-N^{T}\cdot ln(c)\right)$$

Taking the logarithm of both sides, we have

$$ln\left(\frac{v_{f}}{v_{r}}\right)=ln\left(\frac{k_{f}}{k_{r}}\right)-N^{T}\cdot ln(c).$$

Using the thermodynamic constraints on kinetic parameters in (15) we observe that

$$\begin{array}{rr} ln\left(\frac{v_{f}}{v_{r}}\right) & =-N^{T}\cdot \frac{u^{\circ }}{𝓡𝒯}-N^{T}\cdot ln(c),\\ 𝓡𝒯\;ln\left(\frac{v_{f}}{v_{r}}\right) & =-N^{T}\cdot \left(u^{\circ }+𝓡𝒯ln(c)\right). \end{array}$$

Using the definition of chemical potential in Eq. (178) we then obtain

(17) $$𝓡𝒯\;ln\left(\frac{v_{f}}{v_{r}}\right)=-N^{T}\cdot u$$

which is a thermodynamic constraint on reaction rates that must hold for any pair of forward and reverse rates and any potential at a given instance, regardless of whether a system is at equilibrium or not, and regardless if a system is in a dynamic or steady state. Eq. (17) incorporates a representation of energy conservation, since each species is assigned a single chemical potential. Eq. (17) also incorporates a representation of the second law of thermodynamics, since

$$v_{f}=v_{r}\;\Leftrightarrow \;N^{T}\cdot u=0$$

$$v_{f}>v_{r}\;\Leftrightarrow \;N^{T}\cdot u<0$$

$$v_{f}<v_{r}\;\Leftrightarrow \;N^{T}\cdot u>0$$

which ensures that the net rate of each reaction is zero at thermodynamic equilibrium, and away from thermodynamic equilibrium the sign of net rate is opposite to the sign of change in chemical potential, i.e. , net rate is down a gradient in chemical potential.

### 3.10 A thermodynamically open system admitting an elementary kinetic steady state

Given a stoichiometric matrix $N\in Z^{m\times n}$ and a moiety incidence matrix $L\in Z_{\geq 0}^{(m-r)\times m}$ consider the *cyclic stoichiometric matrix* [27]

(18) $$C:=\left[\begin{array}{cc} N & -I\\ 0 & L \end{array}\right]\in Z^{\left(2m-r)\times (n+m\right)}$$

where each of the additional m columns is termed a *perpetireaction*, which is a modelling construct that represents the transformation of a single metabolite into its constituent conserved moieties. Appendix C establishes that rank(C)=m, the matrix

(19) $$\left[\begin{array}{c} I_{n}\\ N \end{array}\right]\in Z^{(n+m)\times n}$$

is a basis for the right nullspace of C, and the matrix $\left[L,\;I_{m-r}\right]\in N^{\left(m-r)\times (2m-r\right)}$ is a basis for the left nullspace of C. Since every row of 19 contains at least one non-zero, every reaction in C participates in at least one vector in the nullspace of C. We shall refer back to this property in a subsequent section. Since ${\left[\begin{array}{rr} L & I \end{array}\right]}^{T}1>0$ every reaction in C is stoichiometrically consistent [31] so by Theorem 1, the corresponding elementary kinetic system admits at least one steady state.

Assume a system of chemical reactions defined by a cyclic stoichiometric matrix 18 with corresponding parameter vectors $k_{f},k_{r}\in R^{n}$ and $p_{f},p_{r}\in R^{m}$. If both are thermodynamically feasible then the Hadamard divisors of forward and reverse parameters form a vector in the range of the cyclic stoichiometric matrix, $𝓡\left(C^{T}\right)$. That is, there exists a $u\in R^{m}$ and $q\in R^{m-r}$ such that

(20) $$\left[\begin{array}{r} ln\left(k_{f}⊘k_{r}\right)\\ ln\left(p_{f}⊘p_{r}\right) \end{array}\right]={\left[\begin{array}{cc} N & -I\\ 0 & L \end{array}\right]}^{T}\cdot \left[\begin{array}{r} u\\ q \end{array}\right]\in 𝓡\left(C^{T}\right).$$

Equivalently

$$\begin{array}{rr} ln\left(k_{f}⊘k_{r}\right) & =N^{T}\cdot u,\\ ln\left(p_{f}⊘p_{r}\right) & =-u+L^{T}\cdot q. \end{array}$$

In contrast, if both $k_{f},k_{r}$ and $p_{f},p_{r}$ are thermodynamically infeasible then the Hadamard divisors of forward and reverse parameters form a vector in the nullspace of the cyclic stoichiometric matrix 𝒩(C). That is

$$\left[\begin{array}{cc} N & -I\\ 0 & L \end{array}\right]\cdot \left[\begin{array}{r} ln\left(k_{f}⊘k_{r}\right)\\ ln\left(p_{f}⊘p_{r}\right) \end{array}\right]=0,$$

or equivalently

$$\begin{array}{rr} N\;ln\left(k_{f}⊘k_{r}\right) & =ln\left(p_{f}⊘p_{r}\right),\\ L\;ln\left(p_{f}⊘p_{r}\right) & =0. \end{array}$$

However, if $k_{f},k_{r}$ are thermodynamically feasible but $p_{f},p_{r}$ are thermodynamically infeasible, then there exists a $u\in R^{m}$ such that

$$\begin{array}{rr} ln\left(k_{f}⊘k_{r}\right) & =N^{T}\cdot u,\\ L\;ln\left(p_{f}⊘p_{r}\right) & =0. \end{array}$$

Let $\overset{‾}{N}\in Z^{m\times r}$ denote a basis for the range of the internal reactions 𝓡(N), then

(21) $$ln\left(p_{f}⊘p_{r}\right)=\overset{‾}{N}w\Rightarrow Lln\left(p_{f}⊘p_{r}\right)=0$$

where $w\in R^{r}$. Without loss of generality, let $ln\left(p_{r}\right)=0$, therefore $ln\left(p_{f}\right)=\overset{‾}{N}w$. Let $\ell \in R_{>0}^{m-r}$ denote the concentration of each conserved moiety. Given a cyclic stoichiometric matrix (18), with the aforementioned parameterisation, the dynamical equation for the corresponding elementary reaction kinetic system is

(22) $$\left[\begin{array}{cc} N & -I\\ 0 & L \end{array}\right]exp\left(\left[\begin{array}{c} ln\left(k_{f}\right)\\ ln\left(p_{f}\right) \end{array}\right]+{\left[\begin{array}{cc} F & I\\ 0 & 0 \end{array}\right]}^{T}\cdot \left[\begin{array}{c} ln(c)\\ ln(\ell ) \end{array}\right]\right)\ldots$$

(23) $$-\left[\begin{array}{cc} N & -I\\ 0 & L \end{array}\right]\exp\left(\left[\begin{array}{c} \ln\left(k_{r}\right)\\ \ln\left(p_{r}\right) \end{array}\right]+{\left[\begin{array}{cc} R & 0\\ 0 & L \end{array}\right]}^{T}\cdot \left[\begin{array}{c} \ln\left(c\right)\\ \ln\left(\ell \right) \end{array}\right]\right)=\left[\begin{array}{c} \dot{c}\\ \dot{\ell } \end{array}\right],$$

and steady states satisfy

(24) $$(R-F)\cdot \left(exp\left(ln\left(k_{f}\right)+F^{T}\cdot ln(c)\right)-exp\left(ln\left(k_{r}\right)+R^{T}\cdot ln(c)\right)\right)\ldots$$

(25) $$-(R-F)\left(exp\left(ln\left(p_{f}\right)+ln(c)\right)+exp\left(ln\left(p_{r}\right)+L^{T}\cdot ln(\ell )\right)\right)=0,$$

(26) $$L\left(exp\left(ln\left(p_{f}\right)+ln(c)\right)-exp\left(ln\left(p_{r}\right)+L^{T}\cdot ln(\ell )\right)\right)=0.$$

By Theorem (1), we are assured a solution to (24) and (26) exists. Moreover, since (26) is implied by (24), then (26) is redundant.

We now show that thermodynamic infeasibility of the exchange parameters forces the guaranteed steady state out of equilibrium. A system of cyclic mass-action kinetics is detailed balanced when every forward elementary rate equals its reverse, $v_{f}=v_{r}$. The following result shows that this cannot occur once the perpeti parameters violate the Wegscheider conditions, even when the internal kinetic parameters $k_{f},k_{r}$ remain thermodynamically feasible.

#### Theorem 2.

*Consider the cyclic stoichiometric system*(18) *with parameters* $k_{f},k_{r}\in R^{n}$ *and* $p_{f},p_{r}\in R^{m}$, *and let a steady state be guaranteed by* Theorem 1. *Assume* $k_{f},k_{r}$ *are thermodynamically feasible but* $p_{f},p_{r}$ *are thermodynamically infeasible, so that there exist* $u\in R^{m}$ *and* $w\in R^{r}$ *such that*

$$ln\left(k_{f}⊘k_{r}\right)=N^{T}\cdot u,\;ln\left(p_{f}⊘p_{r}\right)=\overset{‾}{N}w,$$

*where* $\overset{‾}{N}$ *is a basis for the range* 𝓡(N). *Then the steady state does not satisfy detailed balance; that is*, $v_{f}\neq v_{r}$.

*Proof.* By (20) the cyclic system is thermodynamically feasible exactly when the combined log-parameter vector lies in $𝓡\left(C^{T}\right)$. Since (19) is a basis for the right nullspace of C and $𝓡\left(C^{T}\right)=𝒩(C{)}^{⊥}$, this membership is equivalent to the single cycle condition obtained by pairing the parameter vector with that basis,

(27) $$a:={\left[\begin{array}{c} I_{n}\\ N \end{array}\right]}^{T}\cdot \left[\begin{array}{r} ln\left(k_{f}⊘k_{r}\right)\\ ln\left(p_{f}⊘p_{r}\right) \end{array}\right]=ln\left(k_{f}⊘k_{r}\right)+N^{T}\cdot ln\left(p_{f}⊘p_{r}\right)=0.$$

With $ln\left(k_{f}⊘k_{r}\right)=N^{T}\cdot u$ and $ln\left(p_{f}⊘p_{r}\right)=\overset{‾}{N}w$, condition (27) reads $N^{T}\cdot (u+\overset{‾}{N}w)=0$; the assumed thermodynamic infeasibility of $p_{f},p_{r}$ is precisely the failure of this identity, so a≠0. Suppose now, to the contrary, that the steady state were detailed balanced, that is $v_{f}=v_{r}$. Then the internal net flux $v:=v_{f}-v_{r}$ vanishes, and the species balance (24), which reads Nv=b for the perpeti net flux $b:=p_{f}⊙c-p_{r}⊙exp\left(L^{T}\cdot ln\;\ell \right)$, forces b=0 as well. Every forward rate then equals its reverse, so there is a state (c,ℓ) with $ln\left(k_{f}⊘k_{r}\right)=N^{T}\cdot ln\;c$ and $ln\left(p_{f}⊘p_{r}\right)=-ln\;c+L^{T}\cdot ln\;\ell$; equivalently

$$\left[\begin{array}{r} ln\left(k_{f}⊘k_{r}\right)\\ ln\left(p_{f}⊘p_{r}\right) \end{array}\right]=C^{T}\cdot \left[\begin{array}{r} ln\;c\\ ln\;\ell \end{array}\right]\in 𝓡\left(C^{T}\right).$$

Pairing this with the nullspace basis(19) and using $C\left[I_{n};N\right]=0$ gives $a={\left[I_{n};N\right]}^{T}\cdot C^{T}\cdot [ln\;c;ln\;\ell ]={\left(C\left[I_{n};N\right]\right)}^{T}$. [lnc;lnℓ]=0, contradicting a≠0. Hence $v_{f}\neq v_{r}$, so the internal reactions carry a nonzero net flux and the steady state does not satisfy detailed balance. □

The obstruction is the single cycle affinity a in (27): thermodynamic feasibility of the full cyclic system is equivalent to a=0. By (21) the infeasible exchange parameters satisfy $L\;ln\left(p_{f}⊘p_{r}\right)=0$, so $ln\left(p_{f}⊘p_{r}\right)\in 𝓡(N)$. The entire thermodynamic driving therefore lies within the internal cycle space 𝓡(N), and it is the perpeti reactions that sustain the resulting nonequilibrium steady state. Note that detailed balance is the condition $v_{f}=v_{r}$; Theorem2 asserts its negation.

The strictly positive conservation vector required by Theorem1 is furnished by the cyclic construction itself. Every left-null vector of C has the form $\ell =\left[L^{T}\cdot y;y\right]$ with $y\in R^{m-r}$: its metabolite block is $L^{T}\cdot y$ and its moiety block is y. Choosing y>0, and recalling that L≥0 with every metabolite belonging to at least one conserved moiety, so that each column of L contains a positive entry, one obtains $L^{T}\cdot y>0$ and hence ℓ>0 strictly. The cyclic matrix C is therefore constructed precisely so that the strictly positive left-null vector demanded by Theorem1 is guaranteed to exist, and this vector depends only on the structural matrices N and L, not on any kinetic parameter values. In particular, arbitrarily setting the parameters $p_{f},p_{r}$ but keeping C,L or N invariant, means ℓ>0 is preserved and a steady state continues to exist. Consistently with Theorem2, thermodynamic infeasibility of the perpeti parameters is not merely permitted but is the mechanism that drives this guaranteed steady state away from equilibrium, so that the internal reactions carry a nonzero net flux.

### 3.11 Mathematical classification of elementary reaction kinetics

Classification of a function in mathematical terms is important when one seeks to identify whether there exist established algorithms and software that either enables one to obtain a numerical solution that is in the zero set of that function, or numerically optimise over the zero set of that function. This section attempts to mathematically classify the function (6), and can be omitted on a first pass, but is a topic we shall return to in the discussion. Recall that $Q\in R^{m\times m}$ is positive definite if $x^{T}\cdot Q\cdot x>0$ for all $x\in R^{m}\neq 0$ while A is indefinite if there exist $x_{1}$ and $x_{2}$ such that $x_{1}^{T}\cdot A\cdot x_{1}>0$ and $x_{2}^{T}\cdot Ax_{2}<0$.

Given the cyclic stoichiometric matrix in 18, the corresponding forward and reverse stoichiometric matrices are denoted

$$\overset{‾}{F}:=\left[\begin{array}{cc} F & I_{m}\\ 0 & 0 \end{array}\right]\;\overset{‾}{R}:=\left[\begin{array}{cc} R & 0\\ 0 & L \end{array}\right]\in R^{\left(2m-r)\times (n+m\right)}.$$

Consider the following coordinate transformations

$$\begin{array}{rr} k & :=\left[ln\left(k_{f}\right);ln\left(p_{f}\right);ln\left(k_{r}\right);ln\left(p_{r}\right)\right],\\ x & :=[ln(c);ln(\ell )]. \end{array}$$

Therefore the set of steady sates is

(28) $$f(x):=([\overset{‾}{R},\overset{‾}{F}]-[\overset{‾}{F},\overset{‾}{R}])\cdot exp\left(k+[\overset{‾}{F},\overset{‾}{R}{]}^{T}\cdot x\right)=0,\;\;\;=[\overset{‾}{R},\overset{‾}{F}]\cdot exp\left(k+[\overset{‾}{F},\overset{‾}{R}{]}^{T}\cdot x\right)-[\overset{‾}{F},\overset{‾}{R}]\cdot exp\left(k+[\overset{‾}{F},\overset{‾}{R}{]}^{T}\cdot x\right)=0.$$

By definition $\left[\overset{‾}{F},\overset{‾}{R}\right]$ has non-negative entries. Furthermore, under biochemically realistic assumptions [1], $\left[\overset{‾}{F},\overset{‾}{R}\right]$ is full row rank. This is a form of kinetic consistency, in the sense that the stoichiometric signatures of the metabolites across the forward and reverse elementary reactions, are linearly independent, and it underpins the duality between fluxes and concentrations in biochemical networks [1]. Define the following split of f(x) into two parts

f(x):=∇φ(x)-a(x)

where

$$\begin{array}{rr} \varphi (x) & :=1^{T}\cdot exp\left(k+[\overset{‾}{F},\overset{‾}{R}{]}^{T}\cdot x\right),\\ \nabla \varphi (x) & =[\overset{‾}{F},\overset{‾}{R}]\cdot exp\left(k+[\overset{‾}{F},\overset{‾}{R}{]}^{T}\cdot x\right)>0,\\ \nabla^{2}\varphi (x) & =[\overset{‾}{F},\overset{‾}{R}]\cdot diag\left(exp\left(k+[\overset{‾}{F},\overset{‾}{R}{]}^{T}\cdot x\right)\right)\cdot [\overset{‾}{F},\overset{‾}{R}{]}^{T},\\ a(x) & :=[\overset{‾}{R},\overset{‾}{F}]\cdot exp\left(k+[\overset{‾}{F},\overset{‾}{R}{]}^{T}\cdot x\right)>0,\\ \nabla a(x) & =[\overset{‾}{F},\overset{‾}{R}]\cdot diag\left(exp\left(k+[\overset{‾}{F},\overset{‾}{R}{]}^{T}\cdot x\right)\right)\cdot [\overset{‾}{R},\overset{‾}{F}{]}^{T},\\ \nabla f(x) & =\nabla^{2}\varphi (x)-\nabla a(x). \end{array}$$

Since exp is a convex function and $\left[\overset{‾}{F},\overset{‾}{R}\right]$ is full row rank then φ(x) is a strictly convex function of x, so its gradient $\nabla \varphi (x)\in R_{\geq 0}^{m}$ is strictly monotone and is equal to the rate of consumption of each species, and $\nabla^{2}\varphi (x)\in R^{m\times m}$ is a symmetric positive definite matrix. Full row rank of $\left[\overset{‾}{F},\overset{‾}{R}\right]$ makes the Hessian $\nabla^{2}\varphi (x)$ is positive definite rather than merely positive semidefinite. In contrast, $a(x)\in R_{\geq 0}^{m}$ is the rate of production of each species and $\nabla a(x)\in R^{m\times m}$ is an asymmetric indefinite matrix, so it is not the gradient of any scalar valued function. Also, the Hessian of a strictly convex function is positive definite and vice versa. Therefore ∇f(x) is the difference between a positive definite and an asymmetric square matrix, which may be indefinite, in which case ∇f(x) is not the Hessian of any convex function [32]. This makes it difficult to solve for x such that f(x)=0 with established algorithms using convex optimisation or monotone variational inequality theory [33]. For a restricted class of biochemical networks, previously we established that f(⋅) is not monotone, however it is *duplomonotone*, that is $f(x{)}^{T}\cdot \nabla f(x)\cdot f(x)\geq 0$. This enabled formulation of a globally convergent algorithm whose stationary states solve f(x)=0, but it is not known if f(⋅) is duplomonotone in general [34]. Moreover, even if f(⋅) is duplomonotone, there still remains the unsolved problem to optimise over the set Ω:={x∣f(x)=0}.

Note that, to express concentration dynamics in the logarithmic coordinate, differentiate c=exp(x) componentwise

$$\dot{c}=exp(x)⊙\dot{x}=exp(x)⊙\dot{x}.$$

Because exp(x) is componentwise invertible the velocity field in the logarithmic coordinate is

$$\dot{x}=exp(x)⊙\cdot \dot{c}=-exp(x)⊙f(x).$$

The steady-state set is identical in both coordinates,

$$\dot{c}=0\;⟺\;\dot{x}=0\;⟺\;f(x)=0.$$

As such, up to coordinate transformation, f(x)=0 is the fundamental equation defining the set of steady states of a network.

## 4 Constraint-based modelling of biochemical networks

Instead of trying to directly find a solution to Eq (9), or in addition Eq. (16), the field of constraint-based modelling of biochemical networks arose, whereby a subset of the constraints represented by (16) are either removed or relaxed, and an optimisation problem was formulated to select an optimal vector that also satisfied the resulting simplified system of equations. The motivation for introducing the particular optimisation problems in this section is that each retains a subset of the (primal) constraints on a kinetically feasible steady state but their objectives are different and each of their optimality conditions correspond to different subsets of the feasible set of kinetically feasible steady states. In particular, as explained in Section 7, Theorem 4 establishes that these subsets form a nested chain of sets ${𝒮}_{4}\subseteq {𝒮}_{3}\subseteq {𝒮}_{2}\subseteq {𝒮}_{1}$, with the variational kinetics algorithm optimising towards the innermost set ${𝒮}_{4}$ of elementary kinetic steady states. This is further interpreted in Section 7. The first such optimisation problem was a linear optimisation problem.

### 4.1 Linear optimisation: Introduction

The standard form of a linear optimisation problem is

(29) $$\begin{array}{r} \underset{x}{min}\;c^{T}\cdot x\\ \text{s.t.}\;A\cdot x\leq b \end{array}$$

where $c\in R^{n}$ is a coefficient vector, $c^{T}\cdot x$ is a *linear objective function*, $x\in R^{n}$ is a vector of variables $c\in R^{n}$ is a given vector of coefficients, $A\in R^{m\times n}$ is a given linear constraint matrix and $b\in R^{m}$ is a given vector of data. The constraints in Eq. (29) define a polyhedral convex set, which may either be empty (no solution exists), admit one solution (hence no need for an optimisation problem) or admit an infinite number of solutions (well posed optimisation problem). Optimisation of the objective function is expressed as minimisation by convention and results in identification of an optimal vector $x^{⋆}$ wherein the value of $c^{T}\cdot x^{⋆}$ is minimal, i.e. there does not exist another vector satisfying A⋅x≤b such that the value of the objective is any less. Two distinct vectors $x_{1}^{⋆}$ and $x_{2}^{⋆}$ are referred to as alternate optimal solutions if $c^{T}\cdot x_{1}^{⋆}=c^{T}\cdot x_{2}^{⋆}$. In general, there exist an infinite number of optimal vectors $x^{⋆}$ that each have the same minimal value of the objective function.^2^

### 4.2 Flux balance analysis

Rather than modelling a system of reactions in terms of mass action kinetics, an alternative approach is to represent net reaction rate as a variable rather than as a function of kinetic parameters and species concentrations. Recall that when assuming elementary reaction kinetics, net rate is the difference between a pair of unidirectional reactions, each of which is an explicit function of kinetic parameters and species concentrations, cf Eq. (5). An advantage of this approach is that the set of net rate vectors that satisfy steady state is a polyhedral convex set. This enables one to formulate and efficiently solve various optimisation problems where the solution is a rate vector that is optimal with respect to a particular objective function.

When the objective function is linear, this approach is known as *flux balance analysis* and is represented by the optimisation problem

(30) $$\begin{array}{r} \underset{v,w}{min}c^{T}\cdot v+d^{T}\cdot w\\ \text{s.t.}\;N\cdot v+B\cdot w=0 \end{array}$$

(31) l≤v≤u,

where $v\in R^{n}$ is a vector of net rates, one for each internal reaction in the biochemical system and $c\in R^{n}$ is a given vector of linear objective coefficients, one for each internal reaction. Furthermore, to represent the exchange of mass between the system and its environment $w\in R^{k}$ is a vector of net rates, one for each external reaction and $d\in R^{k}$ is a given vector of linear objective coefficients, one for each exchange reaction. Often, in applications c=0 and only one $d_{i}\neq 0$.

As before, $N\in R^{m\times n}$ is a stoichiometric matrix representing internal reactions and B is a stoichiometric matrix representing external reactions. Compare the first constraint in Problem (30) with the steady state constraint in Eq. (7). Both seem similar but they are fundamentally different as in Eq. (7) net rate is a function of concentrations and kinetic parameters, while in Problem (30) net rate is a variable vector that is not a function of any other variables or parameters, which makes it substantially less constrained.

In Problem (30) the last constraint is a set of box constraints, represented by lower and upper bounds on the net rate for each reaction, $l,u\in R^{n}$. Qualitatively, these bounds may be set based on known directionality of biochemical reactions. The convention is that net rate is positive for a reaction that proceeds from substrates to products in a left to right direction when expressed as a reaction equation. In most metabolic networks, irreversible reactions are represented with bounds like 0≤v≤u so net rate must be positive. Reversible reactions are represented as -∞≤v≤∞ which means that box constraint is not active. Quantitatively such bounds may be set based on experimentally measured reaction rates, e.g., in an *in vitro* culture, by measuring the difference between fresh and spent medium concentrations, one may estimate the rate of uptake or secretion of a metabolite by a system, which can be used to set quantitative bounds on exchange reaction rates. Generally, there are also box constraints on net external reaction rates.

### 4.3 Cycle-free flux balance analysis

Inspired by Desouki et al. [6], we previously observed [35] that a thermodynamically feasible flux may be computed by a single linear optimisation problem

(32) $$\underset{z,w}{min}\;\|v{\|}_{1}+c^{T}\cdot w\;\text{s.t.}\;N\cdot v+B\cdot w=0:y\;\;\;l\leq v\leq u:s\;\;\;l_{w}\leq w\leq u_{w}:t$$

where l and u denote lower and upper bounds on internal reaction fluxes, with the constraint that $l\in \{0,-\infty {\}}^{n}$ and $u\in \{0,\infty {\}}^{n}$, while $l_{w}\in R^{k}$ and $u_{w}\in R^{k}$ denote lower and upper bounds on external reaction fluxes, respectively. The vectors $y\in R^{m},s\in R^{m}$ and $t\in R^{m}$, are dual variables to the steady state constraint, bounds on internal reaction rates and bounds on external reaction rates, respectively.

The optimality conditions of Problem 32 are

$$\begin{array}{rr} N\cdot v^{⋆}+B\cdot w^{⋆} & =0\\ \nabla {\left\|v^{⋆}\right\|}_{1}=sign\left(v^{⋆}\right) & =-N^{T}\cdot y^{⋆}-s^{⋆}\\ c & =-B^{T}\cdot y^{⋆}-t^{⋆} \end{array}$$

where $-y^{⋆}$ may interpreted as a vector proportional to the chemical potentials of each metabolite and $-N_{j}^{T}\cdot y^{⋆}$ is proportional to the change of chemical potential for reaction j. When $l\in \{0,-\infty {\}}^{n}$ and $u\in \{0,\infty {\}}^{n}$ then the optimal dual variable $s_{j}^{⋆}$ to the inequality constraints on internal reaction j, is non-zero if and only if $v_{j}^{⋆}$ is zero, that is $z_{j}^{⋆}=0⟺s_{j}^{⋆}\neq 0$ and $v_{j}^{⋆}\neq 0⟺s_{j}^{⋆}=0$. Therefore $v_{j}^{⋆}\neq 0⟺sign\left(v_{j}^{⋆}\right)=-N_{j}^{T}\cdot y^{⋆}$, which enforces energy conservation and the second law of thermodynamics on the optimal vector of nonzero internal reaction fluxes [8]. However, when $z_{j}=0$, this is a relaxation of the thermodynamic sign constraint $sign\left(v_{j}\right)=-sign\left(N_{j}^{T}\cdot y\right)$, since $v_{j}^{⋆}=0⟺v_{j}^{⋆}=N_{j}^{T}\cdot y^{⋆}+s_{j}^{⋆}=0$ , so $v_{j}^{⋆}=0⟺N_{j}^{T}\cdot y^{⋆}=-s_{j}^{⋆}$ and therefore $v_{j}=0⇎N_{j}^{T}\cdot y=0$. Biochemically, one may interpret this relaxation as saying that a zero internal reaction flux does not imply a zero change in chemical potential. For example, a nonzero change in chemical potential may still be consistent with zero net flux when an enzyme is absent for the corresponding reaction. To summarise, herein we define thermodynamic consistency as the requirement that any nonzero net flux be driven by a change in chemical potential for the corresponding reaction, that is

$$\begin{array}{c} v_{j}^{⋆}>0\Rightarrow N_{j}^{T}\cdot y^{⋆}<0,\\ v_{j}^{⋆}<0\Rightarrow N_{j}^{T}\cdot y^{⋆}>0. \end{array}$$

However, a reactions must admit a non-zero flux that is thermodynamically consistent to be deemed thermodynamically flux consistent, so we omit reactions where zero net flux is the only thermodynamically consistent solution obtained.

### 4.4 Convex optimisation: Introduction

#### 4.4.1 Convex functions

In convex optimisation, the constraints define a polyhedral convex, set while the objective may be non-linear, but it must be *convex function*. The *epigraph* of a function is the set of points lying on or above the function’s graph. A function is convex if and only if its epigraph is a convex set. The function $\phi (x):R^{n}\to R$ given by $x^{T}\cdot x$ is convex. Given $h\in R^{n}$, the quadratic function $\phi (x|h):R^{n}\to R$, given by

$$\phi (x|h):=1/2(h-x{)}^{T}\cdot (h-x)$$

is convex. The exponential function $\phi (x):R^{n}\to R$, given by

$$\phi (x):=1^{T}\cdot exp(x)$$

is convex. The negative entropy function $\phi (x):R_{\geq 0}^{n}\to R$, given by

$$\phi (x):=x^{T}\cdot ln(x)$$

is also convex.

#### 4.4.2 Standard form convex optimisation

The standard form of a convex optimisation problem is

(33) $$\begin{array}{r} \underset{x}{min}\phi (x)\\ \text{s.t.}\;A\cdot x\leq b \end{array}$$

where ϕ(x) is a *convex objective function*, $x\in R^{n}$ is a vector of variables, $A\in R^{m\times n}$ is a given linear constraint matrix and $b\in R^{m}$ is a given vector of data.

### 4.5 Entropic flux balance analysis

There are several shortcomings with flux balance analysis. An important one is that the prediction of internal reaction rate may not, and for genome-scale computational models usually do not, satisfy thermodynamic constraints. In particular do not satisfy energy conservation and the second law of thermodynamics. Previously, we developed a novel method to satisfy the aforementioned constraints using a convex optimisation problem [8]. Since the negative entropy function is convex, the following is a convex optimisation problem

() $$\begin{array}{ll} \underset{v_{f},v_{r}>0}{min} & v_{f}^{T}\cdot ln\left(v_{f}\right)+v_{r}^{T}\cdot ln\left(v_{r}\right)\\ \text{s.t.} & N\cdot \left(v_{f}-v_{r}\right)+B\cdot w=0\;:y \end{array}$$

where we introduce the dual variable $y\in R^{m}$, which by convention is written to the right hand side of the primal constraints. The Lagrangian for this problem is

$$𝓛\left(v_{f},v_{r},w,y\right)=v_{f}^{T}\cdot ln\left(v_{f}\right)+v_{r}^{T}\cdot ln\left(v_{r}\right)-y^{T}\cdot \left(N\cdot \left(v_{f}-v_{r}\right)+B\cdot w\right)$$

and by setting its partial derivatives to equal zero we obtain the optimality conditions for Problem (), which are

(34) $$\frac{\partial 𝓛}{\partial v_{f}}=ln\left(v_{f}^{⋆}\right)+1+N^{T}\cdot y^{⋆}=0$$

(35) $$\frac{\partial 𝓛}{\partial v_{r}}=ln\left(v_{r}^{⋆}\right)+1-N^{T}\cdot y^{⋆}=0\;\frac{\partial 𝓛}{\partial w}=B^{T}\cdot y^{⋆}=0\;\frac{\partial 𝓛}{\partial y}=N\cdot \left(v_{f}^{⋆}-v_{r}^{⋆}\right)+B\cdot w^{⋆}=0$$

By subtracting (35) from (34) we obtain

(36) $$\begin{array}{rr} ln\left(v_{f}^{⋆}\right)-ln\left(v_{r}^{⋆}\right)+2N^{T}\cdot y^{⋆} & =0\\ ln\left(\frac{v_{f}^{⋆}}{v_{r}^{⋆}}\right) & =-2N^{T}\cdot y^{⋆}. \end{array}$$

Comparing (36) with (17), we can see that the optimality conditions of Problem () satisfy the desired thermodynamic constraints and $2y^{*}$ can be interpreted as a vector proportional to the chemical potential of each species.

Consider Problem () with the addition of box constraints and a linear objective on external net rates

(37) $$\underset{v_{f},v_{r},w}{min}v_{f}^{T}\cdot ln\left(v_{f}\right)+v_{r}^{T}\cdot ln\left(v_{r}\right)+d^{T}\cdot w\;\;\;\;\text{s.t.}\;N\cdot \left(v_{f}-v_{r}\right)+B\cdot w=0\;:-y$$

(38) $$l-\left(v_{f}-v_{r}\right)\leq 0\;:-z_{l}$$

(39) $$\left(v_{f}-v_{r}\right)-u\leq 0\;:-z_{u}$$

where $l,u\in R^{n}$ are given lower and upper bounds on internal net rate and where $y\in R^{m},z_{l}\in R_{\geq 0}^{n}$ and $z_{u}\in R_{\geq 0}^{n}$ are dual variables to the steady state constraints, lower bounds on internal net rate, and upper bounds on internal net rate, respectively. A dual variable to an equality constraint may be positive or negative, while a dual variable to an inequality constraint must be restricted in sign. By convention, they are non-negative. The Lagrangian for Problem (37) is

(40) $$𝓛\left(v_{f},v_{r},w,y,z_{l},z_{u}\right):=v_{f}^{T}\cdot ln\left(v_{f}\right)+v_{r}^{T}\cdot ln\left(v_{r}\right)-y^{T}\cdot \left(N\cdot \left(v_{f}-v_{r}\right)+B\cdot w\right)-z_{l}^{T}\cdot \left(l-\left(v_{f}-v_{r}\right)\right)-z_{u}^{T}\cdot \left(\left(v_{f}-v_{r}\right)-u\right)+d^{T}\cdot w$$

and its partial derivatives are

(41) $$\frac{\partial 𝓛}{\partial v_{f}}=ln\left(v_{f}^{⋆}\right)+1-N^{T}\cdot y^{⋆}+z_{l}^{⋆}-z_{u}^{⋆}=0$$

(42) $$\frac{\partial 𝓛}{\partial v_{r}}=ln\left(v_{r}^{⋆}\right)+1+N^{T}\cdot y^{⋆}-z_{l}^{⋆}+z_{u}^{⋆}=0\;\frac{\partial 𝓛}{\partial w}=d-B^{T}\cdot y^{⋆}=0\;\frac{\partial 𝓛}{\partial y}=N\cdot \left(v_{f}^{⋆}-v_{r}^{⋆}\right)+B\cdot w^{⋆}=0$$

Additionally, it can be shown (5.5 in [36]) that an optimum solution satisfies

(43) $$z_{l}^{⋆}⊙\left(l-\left(v_{f}^{⋆}-v_{r}^{⋆}\right)\right)=0$$

(44) $$z_{u}^{⋆}⊙\left(\left(v_{f}^{⋆}-v_{r}^{⋆}\right)-u\right)=0$$

where ⊙ denotes the componentwise (Hadamard) product of a pair of vector arguments. These are referred to as *complementary slackness* conditions, because

$$\begin{array}{r} z_{lj}^{⋆}>0\;\Leftrightarrow \;l_{j}-\left(v_{fj}^{⋆}-v_{rj}^{⋆}\right)=0,\\ z_{lj}^{⋆}=0\;\Leftrightarrow \;l_{j}-\left(v_{fj}^{⋆}-v_{rj}^{⋆}\right)<0, \end{array}$$

and the same for the upper bound constraints, under strict complementarity. By subtracting (42) from (41) we obtain

(45) $$\begin{array}{rr} ln\left(v_{f}^{⋆}\right)-ln\left(v_{r}^{⋆}\right)-2N^{T}\cdot y^{⋆}+2\left(z_{l}^{⋆}-z_{u}^{⋆}\right) & =0,\\ ln\left(\frac{v_{f}^{⋆}}{v_{r}^{⋆}}\right) & =2\left(N^{T}\cdot y^{⋆}-\left(z_{l}^{⋆}-z_{u}^{⋆}\right)\right). \end{array}$$

The dual variables to the box constraints in (45) could potentially interfere with satisfaction of 17, so context-specific models must be generated in a thermodynamically consistent way [37], and bounds on net rate must be set in a thermodynamically consistent way to avoid this issue [35]. Essentially, a solution to Problem (37) will be thermodynamically feasible provided Problem (37) is posed in a way that admits a thermodynamically feasible solution [35]. The existence of a thermodynamically feasible solution is necessary, but not sufficient, for the existence of a kinetically feasible solution, as elaborated further below (cf Section (7)).

*Entropic flux balance analysis* is a parameterised variant of Problem () that also enables penalisation of deviation from measured rates [38]. Compared with a variety of constraint-based modelling approaches, entropic flux balance analysis has been shown to enable superior predictions of reaction rates in a context specific model of dopaminergic neuronal metabolism. . However, entropy maximisation alone it has several shortcomings. Each primal solution $\left(v_{f}^{⋆},v_{r}^{⋆}\right)$ to an entropic flux balance analysis problem is a unique function of input parameters, but it is not obvious what the most appropriate parameterisation is. Maximisation of the relative entropy of unidirectional fluxes, with a prior derived from transcriptomic data, has been demonstrated to further increase prediction accuracy [9], which partly addresses the question of the ideal parameters. However, entropy maximisation tends to bias net rate to reactions whose stoichiometric coefficients are large in magnitude because, all else being equal, for a single unit of rate, a reaction with large stoichiometric coefficient will move more mass than one with a small stoichiometric coefficient. This issue arises because of reactions in a model with high molecularity, which are lumped representations of sets of reactions.

With (relative) entropy maximisation, the predicted potentials are linearly dependent, since the stoichiometric matrix is row rank deficient. Therefore, only the predicted change in chemical potential $\left(N^{T}\cdot y^{⋆}\right)$ is unique, not the potential vector $y^{⋆}$ itself. The variables experimentally measured most frequently are species concentrations while (entropic) flux balance analysis predicts rates and change in chemical potential. This makes comparison of measurements and predictions difficult. When concentrations of molecular species are not represented as variables, it makes it difficult to incorporate data on metabolite concentrations. While predicted rates are thermodynamically feasible, they may not satisfy known reaction rate laws and as such they are not guaranteed to be kinetically feasible. Because known reaction rate laws are not represented, an important established feature of (bio)chemistry is not represented, potentially resulting in prediction artefacts. This motivates a search for novel modelling methods that also incorporate kinetic constraints, yet retain the theoretical and numerical advantages of convex optimisation methods.

## 5 Variational elementary kinetics

### 5.1 Conic optimisation: Introduction

In *conic optimisation*, the objective is linear, but the constraints are an intersection of a polyhedral convex set and one or more *convex cones*. A cone 𝒦 is proper when it is (a) closed (contains its boundary or more technically limit points), (b) pointed, 𝒦∩(-𝒦)={0} and (c) it has nonempty interior. A *proper convex cone* defines a convex set. Henceforth, for brevity, cone means proper convex cone. A *conic inequality* is a constraint

x∈𝒦

where 𝒦 is a proper convex cone. Each conic inequality satisfies certain properties, e.g., a conic inequality is preserved by non-negative linear combinations, that is

x∈𝒦,y∈𝒦⇒αx+βy∈𝒦∀α,β≥0.

A simple example of a cone is the set defined by the non-negative orthant,

$${𝒦}_{\geq 0}:=\left\{x\in R^{n}|x\geq 0\right\}\Leftrightarrow x\in {𝒦}_{\geq 0}.$$

The boundary of a cone is denoted int∂𝒦. The boundary of the non-negative orthant cone is where there exists at least one coordinate that is zero, that is

$$\partial {𝒦}_{\geq 0}:=\left\{x\in R^{n}|x\geq 0,\exists x_{j}=0\right\}$$

The interior of a cone is denoted int𝒦. The interior of the non-negative orthant cone is the set of points where all coordinates are strictly positive, that is

$$int{𝒦}_{\geq 0}:=\left\{x\in R^{n}|x>0\right\}.$$

Conic optimisation is focused on optimisation over certain types of cones that admit the expression of a well behaved barrier function, that is a smooth function with mathematical properties that enable an interior point algorithms to enforce feasibility with respect to a conic equality, while optimising within the cone. For example, the *exponential cone* is a 3 dimensional cone defined^3^ as the closure of the set of points that satisfy

(46) $${𝒦}_{exp}:=\left\{\left(x_{1},x_{2},x_{3}\right)\left|x_{1}\geq x_{2}exp\left(\frac{x_{3}}{x_{2}}\right)\right.,x_{1},x_{2}>0\right\}\Leftrightarrow \left(\begin{array}{c} x_{1}\\ x_{2}\\ x_{3} \end{array}\right)\in {𝒦}_{exp}\subset R^{3}.$$

Observe that the epigraph of an exponential function is a two dimensional slice of an exponential cone where $x_{2}=1$, that is

$$\left(\begin{array}{c} x_{1}\\ 1\\ x_{3} \end{array}\right)\in {𝒦}_{exp}\Leftrightarrow x_{1}\geq exp\left(x_{3}\right),x_{1}>0.$$

Equivalently, the exponential cone may be defined in logarithmic rather than exponential terms as the closure of the set of points that satisfy

(47) $${𝒦}_{exp}:=\left\{\left(x_{1},x_{2},x_{3}\right)\left|-x_{3}\geq x_{2}ln\left(\frac{x_{2}}{x_{1}}\right)\right.,x_{1},x_{2}>0\right\}\Leftrightarrow \left(\begin{array}{c} x_{1}\\ x_{2}\\ x_{3} \end{array}\right)\in {𝒦}_{exp}\subset R^{3}.$$

The rotated quadratic cone is a 2+n dimensional cone

$$Q^{2+n}:=\left\{(x,y,z)|2xy\geq z_{1}^{2}+z_{2}^{2}+\ldots +z_{n}^{2},x\geq 0,y\geq 0\right\}\Leftrightarrow \left(\begin{array}{c} x\\ y\\ z_{1}\\ \vdots \\ z_{n} \end{array}\right)\in Q^{2+n},$$

which is a 2+n dimensional cone. Any positive semidefinite matrix may be factorised as $H^{T}\cdot H$, where $H\in R^{k\times n}$ and k=rank(H). Given such a factor H and a vector $h\in R^{n}$ a convex quadratic set can be represented by a rotated quadratic cone of the form

(48) $$t\geq 1/2(h-x{)}^{T}\cdot H^{T}\cdot H\cdot (h-x)\Leftrightarrow \left(\begin{array}{c} t\\ 1\\ H\cdot (x-h) \end{array}\right)\in {𝒬}^{2+n},$$

which is an affine quadratic constraint.

Any convex constraint can be represented as a conic inequality, with minor modifications to make 𝒦 proper. A cone can be constructed from any convex function $\phi (x):R^{n}\to R$ because the epigraph of the perspective of a convex function is a convex cone. It follows, that any constraint involving a convex function $\phi \left(x_{1}\right):R^{m}\to R$ can be represented as a conic linear inequality, that is

$$x_{3}\geq \phi \left(x_{1}\right)\Leftrightarrow \left(\begin{array}{c} x_{1}\\ 1\\ x_{3} \end{array}\right)\in {𝒦}_{\phi }.$$

Let $A\in R^{m\times n},b\in R^{m},F\in R^{k\times n}$ and $g\in R^{k}$, then the standard form for a *conic optimisation* problem is

(49) $$\begin{array}{cc} \underset{x}{min} & c^{T}\cdot x\\ \text{s.t.} & A\cdot x\leq b, \end{array}$$

(50) F⋅x+g∈𝒦.

In practice, current conic optimisation solvers support a limited number of types of cones, therefore whether a problem can be solved by a given solver depends on which types of cone the solver supports.

### 5.2 Conification of elementary reaction kinetics

Our novel approach is to reformulate Eq. (9) into a system of equations that represent a convex set, which is amenable to optimisation, yet satisfy Eq. (9) at the solution to an optimisation problem. Eq. (9) may be rewritten as

(51) $$N\cdot \left(v_{f}-v_{r}\right)+B\cdot w=0,$$

(52) $$v_{f}=exp\left(ln\left(k_{f}\right)+F^{T}\cdot ln(c)\right),$$

(53) $$v_{r}=exp\left(ln\left(k_{r}\right)+R^{T}\cdot ln(c)\right),$$

which still represents the same non-convex set as Eq. (9). By introducing logarithmic variables in place of kinetic parameters and concentration,

(54) $${lnk}_{f}\;:=ln\left(k_{f}\right),$$

(55) $${lnk}_{r}\;:=ln\left(k_{r}\right),$$

(56) lnc:=ln(c),

then Eq. (51), (52) and (53) become

(57) $$N\cdot \left(v_{f}-v_{r}\right)+B\cdot w=0,$$

(58) $$v_{f}=exp\left({lnk}_{f}+F^{T}\cdot lnc\right),$$

(59) $$v_{r}=exp\left({lnk}_{r}+R^{T}\cdot lnc\right),$$

(60) $$k_{f}=exp\left({lnk}_{f}\right),$$

(61) $$k_{r}=exp\left({lnk}_{r}\right),$$

(62) c=exp(lnc),

which, again, still represents the same non-convex set as Eq. (9). Assume we have a solution $\left(v_{f},v_{r},{lnk}_{f},{lnk}_{r},lnc\right)$ to Eq. (57)–(59), then it is straightforward to compute the exponentials of ${lnk}_{f},{lnk}_{r},lnc$ to obtain $k_{f},k_{r},c$ so for the sake of clarity, we will momentarily omit (60), (61) and (62) from consideration.

Eq. (52) and (53) each involves an exponential term, which can be relaxed by replacing each equality by an inequality to give

(63) $$N\cdot \left(v_{f}-v_{r}\right)+B\cdot w=0,$$

(64) $$v_{f}\geq exp\left({lnk}_{f}+F^{T}\cdot lnc\right),$$

(65) $$v_{r}\geq exp\left({lnk}_{r}+R^{T}\cdot lnc\right),$$

It is clear that Eq. (63) represents a (polyhedral) convex set. However, each of the inequalities (64) and (65) also represents a convex set, even though it is nonlinear. This can be appreciated by recognising that the exponential is a convex function which is the same as saying that the epigraph of an exponential function is a convex set. In fact, each term of the form $x_{1}\geq exp\left(x_{3}\right)$ is a two dimensional plane within an exponential cone (46) with $x_{2}=1$. That is, one may express each of the constraints involving an exponential term as either of the two equivalent forms

$$x_{1}\geq exp\left(x_{3}\right)\Leftrightarrow \left(\begin{array}{c} x_{1}\\ 1\\ x_{3} \end{array}\right)\in {𝒦}_{exp},$$

where it is implicit in this representation that $x_{2}=1$. Therefore, Eq. (63), (64) and (65) may be expressed as

(66) $$N\cdot \left(v_{f}-v_{r}\right)+B\cdot w=0,$$

(67) $$\left(\begin{array}{c} v_{f}\\ 1\\ F^{T}\cdot lnc+{lnk}_{f} \end{array}\right)\in {𝒦}_{exp}^{n},$$

(68) $$\left(\begin{array}{c} v_{r}\\ 1\\ R^{T}\cdot lnc+{lnk}_{r} \end{array}\right)\in {𝒦}_{exp}^{n},$$

where there are a set of n exponential cone constraints, one for each forward reaction, and a second set n exponential cone constraints, one for each reverse reaction. The order of the variables is to be interpreted as set of n componentwise exponential cone constraints, where 1 is a n×1 dimensional vector of constants. That is

$$\left(\begin{array}{c} v_{f}\\ 1\\ F^{T}\cdot lnc+{lnk}_{f} \end{array}\right)\in {𝒦}_{exp}^{n}\Leftrightarrow \left(\begin{array}{c} v_{fj}\\ 1\\ F_{:,j}^{T}\cdot lnc+{lnk}_{f,j} \end{array}\right)\in {𝒦}_{exp}\forall j\in 1\ldots n.$$

Without loss of generality, assume that we are given logarithmic kinetic parameters ${lnk}_{f}$ and ${lnk}_{r}$. Constraints (66)–(68) define a nonlinear, yet convex set, at the boundary of which are solutions to Eq. (57)–(59), which may be obtained at the optimum of the following *conic optimisation* problem

(69) $$\begin{array}{rr} \underset{v_{f},v_{r}w,ln\;c}{min} & c_{v_{f}}^{T}\cdot v_{f}+c_{v_{r}}^{T}\cdot v_{r}+c_{lnc}^{T}\cdot lnc\\ \text{s.t.} & N\cdot \left(v_{f}-v_{r}\right)+B\cdot w=0 \end{array}$$

(70) $$\left(\begin{array}{c} v_{f}\\ 1\\ F^{T}\cdot lnc+{lnk}_{f} \end{array}\right)\in {𝒦}_{exp}^{n}\;⟺\;v_{f}\geq exp\left(F^{T}\cdot lnc+{lnk}_{f}\right)$$

(71) $$\left(\begin{array}{c} v_{r}\\ 1\\ R^{T}\cdot lnc+{lnk}_{r} \end{array}\right)\in {𝒦}_{exp}^{n}\;⟺\;v_{r}\geq exp\left(R^{T}\cdot lnc+{lnk}_{r}\right).$$

This begs the question, how does one choose the parameters $c_{v_{f}},c_{v_{r}}$ and $c_{lnc}$ such that the optimal solution to this problem lies at the boundary of each exponential cone, where $v_{f}^{⋆}=exp\left(F^{T}\cdot {lnc}^{⋆}+{lnk}_{f}\right)$ and $v_{r}^{⋆}=exp\left(R^{T}\cdot {lnc}^{⋆}+{lnk}_{r}\right)$? An answer to this questions requires some additional conic optimisation theory, which is framed in terms of generic conic optimisation problem.

### 5.3 Correspondence with reaction kinetics

The correspondence between the reaction kinetic Problem 69 and the generic conic optimisation Problem 49 can be understood from the following

$$x:=\left[\begin{array}{c} v_{f}\\ v_{r}\\ 1\\ 1\\ lnc\\ w \end{array}\right],\;c:=\left[\begin{array}{c} c_{v_{f}}\\ c_{v_{r}}\\ 0\\ 0\\ c_{lnc}\\ 0 \end{array}\right],\;A:=\left[\begin{array}{rrrrr} N & -N & 0 & 0 & 0 \end{array}\right],\;b:=B\cdot w,$$

and

$$F:=\left[\begin{array}{cccccc} I & 0 & 0 & 0 & 0 & 0\\ 0 & 0 & 0 & I & 0 & 0\\ 0 & 0 & F^{T} & 0 & 0 & 0\\ 0 & I & 0 & 0 & 0 & 0\\ 0 & 0 & 0 & 0 & I & 0\\ 0 & 0 & R^{T} & 0 & 0 & 0 \end{array}\right],g:=\left[\begin{array}{c} 0\\ 0\\ {lnk}_{f}\\ 0\\ 0\\ {lnk}_{r} \end{array}\right],$$

Where

$$F_{1}:=\left[\begin{array}{rrrrrr} I & 0 & 0 & 0 & 0 & 0\\ 0 & I & 0 & 0 & 0 & 0 \end{array}\right],\;g_{1}:=\left[\begin{array}{r} 0\\ 0 \end{array}\right],$$

$$F_{2}:=\left[\begin{array}{rrrrrr} 0 & 0 & 0 & I & 0 & 0\\ 0 & 0 & 0 & 0 & I & 0 \end{array}\right],\;g_{1}:=\left[\begin{array}{c} 0\\ 0 \end{array}\right],$$

$$F_{3}:=\left[\begin{array}{rrrrrr} 0 & 0 & F^{T} & 0 & 0 & 0\\ 0 & 0 & R^{T} & 0 & 0 & 0 \end{array}\right],\;g_{1}:=\left[\begin{array}{c} {lnk}_{f}\\ {lnk}_{f} \end{array}\right],$$

and therefore the exponential cone constraints are

$$F_{1}\cdot x+g_{1}\geq exp\left(F_{3}\cdot x+g_{3}\right),\;F_{2}\cdot x+g_{2}=1,$$

with equality of each row when the corresponding exponential cone constraint is active.

### 5.4 Conic optimisation: optimality conditions

#### 5.4.1 Dual cone

We define a *dual cone* as

$${𝒦}^{⋆}:=\left\{s\in R^{n}|x^{T}\cdot s\geq 0\;\forall x\in 𝒦\right\}.$$

Since $x\in 𝒦,s\in {𝒦}^{⋆}$ and $x^{T}\cdot s\geq 0$ then x and s are referred to as *primal* and *dual* variables, as they reside within primal and dual cones. Where 𝒦 is a proper convex cone, the dual cone ${𝒦}^{⋆}$ has the following properties, (a) it is a proper cone, (b) the dual of the dual cone is the primal cone, that is ${\left({𝒦}^{⋆}\right)}^{⋆}=𝒦$, and (c) the interior of the dual cone $int{𝒦}^{⋆}$ is given by

$$int{𝒦}^{⋆}=\left\{s\in R^{n}|s\neq 0,x\neq 0,x^{T}\cdot s>0\forall x\in 𝒦\right\}.$$

Let $A\in R^{m\times n}$, then dual of a linear subspace is the orthogonal subspace. For example let the nullspace be $𝓛:=\left\{x\in R^{n}|Ax=0\right\}$ then the dual to the nullspace is ${𝓛}^{⊥}={𝓛}^{⋆}$, which is the row space ${𝓛}^{⊥}={𝓛}^{⋆}=\left\{s\in R^{n}|s=A^{T}\cdot z,z\in R^{m}\right\}$. The dual of the exponential cone is the closure

(72) $${𝒦}_{exp}^{⋆}:=cl\left\{\left(s_{1},s_{2},s_{3}\right)\left|s_{1}\geq -s_{3}exp\left(\frac{s_{2}}{s_{3}}-1\right)\right.,s_{1}>0,s_{3}<0\right\}\Leftrightarrow \left(\begin{array}{c} s_{1}\\ s_{2}\\ s_{3} \end{array}\right)\in {𝒦}_{exp}^{⋆}.$$

The non-negative orthant cone

$$𝒦:=R_{\geq 0}^{n}$$

is *self-dual* since the dual of the non-negative orthant is the non-negative orthant, that is

$$R_{\geq 0}^{n}=\left\{s|x^{T}\cdot s\geq 0,\forall x\in R_{\geq 0}^{n}\right\}={𝒦}^{⋆}.$$

#### 5.4.2 Optimality conditions

Let $A\in R^{m\times n},b\in R^{m},F\in R^{3k\times n}$, and $g\in R^{3k}$, then consider the primal *exponential conic linear optimisation* problem

(73) $$\begin{array}{cc} \underset{x}{min} & c^{T}\cdot x\\ \text{s.t.} & A\cdot x=b\;\;:y \end{array}$$

(74) $$F\cdot x+g\in {𝒦}_{\text{exp}}^{k}\;:s$$

where $y\in R^{m}$ is a vector of dual variables to the linear equality constraints and $s\in R^{3k}$ is a vector of dual variables to each conically constrained term. The *Lagrangian* analogue of Problem (73) is

(75) $$𝓛(x,y,s):=c^{T}\cdot x-y^{T}\cdot (A\cdot x-b)-s^{T}\cdot (F\cdot x+g)\;\;\;\;\;F\cdot x+g\in 𝒦$$

(76) $$s\in {𝒦}^{⋆}$$

The gradient of the Lagrangian with respect to x and y are zero at the optimum of Problem (73), that is

$$\begin{array}{l} \frac{\partial 𝓛(x,y,s)}{\partial x}=c-A^{T}\cdot y^{⋆}-F^{T}\cdot s^{⋆}=0\\ \frac{\partial 𝓛(x,y,s)}{\partial y}=b-Ax^{⋆}=0. \end{array}$$

Since F⋅x+g∈𝒦 and $s\in {𝒦}^{⋆}$ we have $s^{T}\cdot (F\cdot x+g)\geq 0$ because they are primal and dual variables. At the optimum of 75 we have the complementarity condition

$$s^{⋆T}\cdot \left(F\cdot x^{⋆}+g\right)=0,$$

because if $s^{T}\cdot (F\cdot x+g)>0$ then it would be possible to further minimise the Lagrangian, which contradicts minimality, therefore $s^{T}\cdot (F\cdot x+g)=0$. This is a complementary condition, rather than complementary slackness as in (43) or (44) as $s^{T}\cdot (F\cdot x+g)=0$ does not imply that s⊙(F⋅x+g)=0.

Actually, there is one complementarity condition involving 3 primal and 3 dual terms per primal exponential conic constraint. Specifically, each cone corresponds to one complementarity constraint of the form

$$s_{1}(F\cdot x+g{)}_{1}+s_{2}(F\cdot x+g{)}_{2}+s_{2}(F\cdot x+g{)}_{2}=0.$$

We now express these conditions for a set of k exponential cones in matrix-vector form. Let $B\in \{0,1{\}}^{k\times 3k}$ be a matrix where k is the number of exponential cones in the cone product ${𝒦}_{\text{exp}}^{k}$, and 3k is the number of rows of F, each corresponding to a conically constrained term. Let $B_{i,j}=1$ if cone i involves conically constrained term j and zero otherwise. Then the set of complementarity conditions may be expressed as $B\cdot \left(s^{⋆}⊙\left(F\cdot x^{⋆}+g\right)\right)=0$. Combining the aforementioned constraints then x,y and s are optimal if and only if

(77) $$A\cdot x^{⋆}-b=0,\;:y^{⋆}$$

(78) $$c-A^{T}\cdot y^{⋆}-F^{T}\cdot s^{⋆}=0,\;:x^{⋆}$$

(79) $$F\cdot x^{⋆}+g\in 𝒦,\;:s^{⋆}$$

(80) $$s^{⋆}\in {𝒦}^{⋆},$$

(81) $$B\cdot \left(s^{⋆}⊙\left(F\cdot x^{⋆}+g\right)\right)=0,$$

which represent the optimality conditions of Problem 73, and the corresponding dual variables.

#### 5.4.3 Primal and dual problems in conic optimisation

Let $F\in R^{k\times n}$ and $g\in R^{k}$. Note that we follow the notation in the conic optimisation community by using F to denote the matrix in the affine conic constraints, which clashes with the use of the same upper case roman letter for forward stoichiometric matrix. Let $c^{⋆}:=c^{T}\cdot x^{⋆}$ denote the optimal value of the objective of the following primal *conic linear optimisation* problem

(82) $$\begin{array}{cc} \underset{x}{min} & c^{T}\cdot x\\ \text{s.t.} & F\cdot x+g\in 𝒦\;:s \end{array}$$

Let $d^{⋆}:=g^{T}\cdot y^{⋆}$ be the optimal value of the following *dual conic linear optimisation* problem

(83) $$\begin{array}{cc} \underset{y}{max} & g^{T}\cdot y\\ \text{s.t.} & c-F^{T}\cdot y=0\;:x \end{array}$$

(84) $$y\in {𝒦}^{⋆}$$

then, without exception, *weak duality* holds

$$c^{⋆}=c^{T}\cdot x^{⋆}\geq d^{⋆}=-g^{T}\cdot y^{⋆}.$$

If the primal or dual problem is strictly feasible, then *strong duality* holds, that is $c^{⋆}=d^{⋆}$. If the primal is *strictly* feasible, then the dual optimum is attained if, $d^{⋆}$ is finite. If $d^{⋆}$ is not finite, a solver will report that the dual problem is unbounded. If the dual is *strictly* feasible, then the primal optimum is attained, if $c^{⋆}$ is finite. If $c^{⋆}$ is not finite, a solver will report that the primal problem is unbounded. This is more restrictive than linear programming duality, where the strong duality holds if the primal or dual problem is feasible (not strictly feasible).

### 5.5 Variational elementary kinetics: optimality conditions

The primal optimisation problem in Problem (69), with the addition of dual variables is

(85) $$\begin{array}{rr} \underset{v_{f},v_{r},w,lnc}{min} & c_{v_{f}}^{T}\cdot v_{f}+c_{v_{r}}^{T}\cdot v_{r}+c_{lnc}^{T}\cdot lnc\\ \text{s.t.} & \;N\cdot \left(v_{f}-v_{r}\right)+B\cdot w=0\;:-y \end{array}$$

(86) $$\left(\begin{array}{c} v_{f}\\ 1\\ F^{T}\cdot lnc+{lnk}_{f} \end{array}\right)\in {𝒦}_{exp}^{n}:-\left(\begin{array}{c} s_{vf}\\ s_{f1}\\ s_{F} \end{array}\right)$$

(87) $$\left(\begin{array}{c} v_{r}\\ 1\\ R^{T}\cdot lnc+{lnk}_{r} \end{array}\right)\in {𝒦}_{exp}^{n}:-\left(\begin{array}{c} s_{vr}\\ s_{r1}\\ s_{R} \end{array}\right)$$

Note that s is used to denote any dual variable to a cone constraint, its subscript is reflective of the corresponding primal term and $s_{vf},s_{vr}s_{f1},s_{r1},s_{F},s_{R}\in R^{n}$. The *Lagrangian* corresponding to Problem (85) is

(88) $$𝓛\left(v_{f},v_{r},w,lnc,y,s\right):=c_{v_{f}}^{T}\cdot v_{f}+c_{v_{r}}^{T}\cdot v_{r}+c_{lnc}^{T}\cdot lnc-y^{T}\cdot \left(N\cdot \left(v_{f}-v_{r}\right)+B\cdot w\right)$$

(89) $$-{\left(\begin{array}{c} s_{vf}\\ s_{f1}\\ s_{F} \end{array}\right)}^{T}\cdot \left(\begin{array}{c} v_{f}\\ 1\\ F^{T}\cdot lnc+{lnk}_{f} \end{array}\right)-{\left(\begin{array}{c} s_{vr}\\ s_{r1}\\ s_{R} \end{array}\right)}^{T}\cdot \left(\begin{array}{c} v_{r}\\ 1\\ R^{T}\cdot lnc+{lnk}_{r} \end{array}\right)$$

(90) $$\left(\begin{array}{c} v_{f}\\ 1\\ F^{T}\cdot lnc+{lnk}_{f} \end{array}\right)\in {𝒦}_{exp}^{n},\left(\begin{array}{c} v_{r}\\ 1\\ R^{T}\cdot lnc+{lnk}_{r} \end{array}\right)\in {𝒦}_{exp}^{n}$$

$$\left(\begin{array}{c} s_{vf}\\ s_{f1}\\ s_{F} \end{array}\right)\in {𝒦}_{exp}^{n⋆},\left(\begin{array}{c} s_{vr}\\ s_{r1}\\ s_{R} \end{array}\right)\in {𝒦}_{exp}^{n⋆}$$

where the last two pairs of terms represent the requirement for primal and dual terms to be constrained to lie within primal and dual exponential cones. The optimality conditions may be obtained by (a) setting the partial derivatives of the Lagrangian with respect to the variables $v_{f},v_{r},w,lnc,y$ to zero, that is (85)

(91) $$\frac{\partial 𝓛}{\partial v_{f}}=c_{v_{f}}-N^{T}\cdot y^{⋆}-s_{vf}^{⋆}=0$$

(92) $$\frac{\partial 𝓛}{\partial v_{r}}=c_{v_{r}}+N^{T}\cdot y^{⋆}-s_{vr}^{⋆}=0$$

$$\frac{\partial 𝓛}{\partial w}=-B^{T}\cdot y^{⋆}=0$$

(93) $$\frac{\partial 𝓛}{\partial lnc}=c_{lnc}-F\cdot s_{F}^{⋆}-R\cdot s_{R}^{⋆}=0$$

$$\frac{\partial 𝓛}{\partial y}=N\cdot \left(v_{f}^{⋆}-v_{r}^{⋆}\right)+B\cdot w^{⋆}=0$$

(b) expressing the complementarity conditions between primal and dual variables, and

(94) $${\left(\begin{array}{c} s_{vf}^{⋆}\\ s_{f1}^{⋆}\\ s_{F}^{⋆} \end{array}\right)}^{T}\cdot \left(\begin{array}{c} v_{f}^{⋆}\\ 1\\ F^{T}\cdot {lnc}^{⋆}+{lnk}_{f} \end{array}\right)=0$$

$$\begin{array}{r} {\left(\begin{array}{c} s_{vr}^{⋆}\\ s_{r1}^{⋆}\\ s_{R}^{⋆} \end{array}\right)}^{T}\cdot \left(\begin{array}{c} v_{r}^{⋆}\\ 1\\ R^{T}\cdot {lnc}^{⋆}+{lnk}_{r} \end{array}\right)=0 \end{array}$$

(c) specifying that the primal and dual terms are constrained to reside within primal and dual conic cones, respectively, with

$$\begin{array}{rr} \left(\begin{array}{c} v_{f}^{⋆}\\ 1\\ F^{T}\cdot {lnc}^{⋆}+{lnk}_{f} \end{array}\right)\in {𝒦}_{exp}^{n} & \left(\begin{array}{c} v_{r}^{⋆}\\ 1\\ R^{T}\cdot {lnc}^{⋆}+{lnk}_{r} \end{array}\right)\in {𝒦}_{exp}^{n}\\ \left(\begin{array}{c} s_{vf}^{⋆}\\ s_{f1}^{⋆}\\ s_{F}^{⋆} \end{array}\right)\in {𝒦}_{exp}^{⋆} & \left(\begin{array}{c} s_{vr}^{⋆}\\ s_{r1}^{⋆}\\ s_{R}^{⋆} \end{array}\right)\in {𝒦}_{exp}^{⋆}n \end{array}$$

where by the definition of the primal exponential cone we have $v_{f}^{⋆},v_{r}^{⋆}>0$ and from the definition of the closure of the dual exponential cone we have $s_{vf}^{⋆},s_{vr}^{⋆}>0$ and $s_{F}^{⋆},s_{R}^{⋆}<0$.

Elementary reaction kinetics requires satisfaction of the constraints

(95) $$\left[\begin{array}{c} v_{f}\\ v_{r} \end{array}\right]=exp\left({\left[\begin{array}{rr} F & ,R \end{array}\right]}^{T}\cdot lnc+\left[\begin{array}{r} {lnk}_{f}\\ {lnk}_{r} \end{array}\right]\right),$$

while the optimality conditions of Problem (85) has relaxed these constraints to

$$\begin{array}{r} \left(\begin{array}{c} v_{f}^{⋆}\\ 1\\ F^{T}\cdot {lnc}^{⋆}+{lnk}_{f} \end{array}\right)\in {𝒦}_{exp}^{n}\;\Leftrightarrow \;v_{f}^{⋆}\geq exp\left(F^{T}\cdot {lnc}^{⋆}+{lnk}_{f}\right),\\ \left(\begin{array}{c} v_{r}^{⋆}\\ 1\\ R^{T}\cdot {lnc}^{⋆}+{lnk}_{r} \end{array}\right)\in {𝒦}_{exp}^{n}\;\Leftrightarrow \;v_{r}^{⋆}\geq exp\left(R^{T}\cdot {lnc}^{⋆}+{lnk}_{r}\right). \end{array}$$

so when any one of these inequalities is strict, the corresponding unidirectional flux is in the interior of an exponential cone. Let $\left(v_{f}^{∙},v_{r}^{∙},{lnc}^{∙}\right)$ denote an optimal solution of Problem (85) that is also at the boundary of the exponential cone, that is

(96) $$\left(\begin{array}{c} v_{f}^{∙}\\ 1\\ F^{T}\cdot {lnc}^{∙}+{lnk}_{f} \end{array}\right)\in \partial {𝒦}_{exp}^{n}\;\Leftrightarrow \;v_{f}^{∙}=exp\left(F^{T}\cdot {lnc}^{∙}+{lnk}_{f}\right),$$

(97) $$\left(\begin{array}{c} v_{r}^{∙}\\ 1\\ R^{T}\cdot {lnc}^{∙}+{lnk}_{r} \end{array}\right)\in \partial {𝒦}_{exp}^{n}\;\Leftrightarrow \;v_{r}^{∙}=exp\left(R^{T}\cdot {lnc}^{∙}+{lnk}_{r}\right),$$

which are identical to constraints in Eq. (95) required for elementary reaction kinetics to hold. Problem (85) has two free parameter vectors $c_{v_{f}}\in R^{n}$ and $c_{v_{r}}\in R^{n}$.

The optimality conditions (91) and (92) relate the free parameters $c_{v_{f}}\in R^{n}$ and $c_{v_{r}}\in R^{n}$ to the dual variables via

(98) $$-c_{v_{r}}+s_{v_{r}}^{⋆}=N^{T}\cdot y^{⋆}=c_{v_{f}}-s_{v_{f}}^{⋆}$$

and since $s_{v_{f}}^{⋆},s_{v_{r}}^{⋆}R_{\geq 0}^{n}$, we have

$$-c_{v_{r}}\leq N^{T}\cdot y^{⋆}\leq c_{v_{f}}$$

which demonstrates that the values of $-c_{v_{r}}$ and $c_{v_{f}}$ are lower and upper bounds on $N^{T}\cdot y^{⋆}$, therefore $-c_{v_{r}}\leq c_{v_{f}}$, therefore $0\leq c_{v_{f}}+c_{v_{r}}$. A sufficient condition for the latter is $c_{v_{f}},c_{v_{r}}\in R_{\geq 0}^{n}$. The optimality condition (93) relates the free parameters $c_{lnc}$ to the dual variables via

$$c_{lnc}=\left[\begin{array}{rr} F & ,R \end{array}\right]\cdot \left[\begin{array}{c} s_{F}^{⋆}\\ s_{R}^{⋆} \end{array}\right]$$

but since $s_{F}^{⋆},s_{R}^{⋆}\leq 0$ and $[F\;,R]\in R_{\geq 0}^{m\times 2n}$, then $c_{lnc}\in R_{\leq 0}^{m}$, that is, the objective coefficients corresponding to logarithmic concentration must be negative. An intuitive explanation of signs of the linear objective coefficients is that prior to a stationary point

$$\left[\begin{array}{c} v_{f}\\ v_{r} \end{array}\right]>exp\left({\left[\begin{array}{rr} F & ,R \end{array}\right]}^{T}\cdot lnc+\left[\begin{array}{r} {lnk}_{f}\\ {lnk}_{r} \end{array}\right]\right),$$

therefore minimising $v_{f}$ and $v_{r}$ and maximising lnc will encourage each exponential cone constraint to be active at an optimal solution. For any choice of positive values for the entries of $c_{f},c_{r}$ and negative values for the entries of $c_{lnc}$ one can obtain an optimal solution to Problem (85) where (96) and (97) are satisfied, provided the linear constraints are omitted. Therefore, in Section (6) we introduce an algorithm, consisting of a iterative sequence of conic optimisation problems, to optimise these parameters and prove its convergence and in Section (7) we demonstrate that it converges to satisfy (96) and (97).

## 6 Convergence of a sequence of conic optimisation problems

### 6.1 Conic optimisation: classes of optimal solutions

In this section, we introduce an algorithm that considers the linear objective coefficient vectors, $\left(c_{f},c_{r},c_{lnc}\right)$ in (85), as parameters to be optimised such that elementary kinetics is satisfied. This algorithm is explained as an abstract sequence of exponential conic linear optimisation Problems, each as in (73), rather than directly in terms of kinetics because the result is more general and the explanation more concise. In Problem 73 a constraint is said to be *active* if perturbing it would change the value of the optimal linear objective. Given the input data {c,A,b,F,g}, optimality conditions in Equations 77 – 81 define an optimal solution to Problem 73 and each non-zero entry in one of optimal variable vectors in the set $\left\{y^{⋆},x^{⋆},s^{⋆}\right\}$ indicates a constraint that is active at an optimal solution. Equivalently, the dual variable corresponding to the active primal constraint, or primal variable corresponding to the active dual constraint is nonzero. For Problem 73, assuming the input data {A,b,F,g} are invariant, it is the vector of linear objective coefficients $c\in R^{n}$ that determines the constraints that are active at an optimal solution. From this perspective, the set of optimal variable vectors $\left\{y^{⋆},x^{⋆},s^{⋆}\right\}$ is a nonlinear function of a parameter c.

The solutions to Problem 73, each a function of a particular linear objective coefficient vector $c\in R^{n}$, may be classed by the combination of optimality constraints that are active, equivalently the set of optimal variable vectors $\left\{y^{⋆},x^{⋆},s^{⋆}\right\}$ that are non-zero. In certain circumstances, we seek a class of optimal solution where each of the conic constraints are active, equivalently each $s^{⋆}\neq 0$. For example, a nonlinear yet convex conic constraint is a relaxation of a desired nonlinear and non-convex constraint, one may seek a optimal solution where that conic constraint is active. For example, given Problem (73), we may seek to identify a $c^{∙}$ such that each exponential cone constraint is active at an optimal solution, equivalently the corresponding dual variables are nonzero, that is $s_{j}^{⋆}\left(c^{∙}\right)\neq 0$ in (77–81) for all j∈1…3k. In the following, we approach the problem of identifying a $c^{∙}$ such that $s_{j}^{⋆}\left(c^{∙}\right)\neq 0$ as a major optimisation problem, where a merit function $\phi :R^{n}\to R$ is minimised subject to constraints, denoted 𝒳, by solving an iterative sequence of minor conic optimisation problems, each of the form 73. First, in Section 6 Theorem 3 demonstrates, in an abstract sense, that this iterative sequence of conic optimisation problems converges to a stationary point of the merit function, subject to the constraints. We do not attempt to demonstrate, for the abstract case, that each stationary point corresponds to a solution where a set of exponential conic constraints is active, because it depends on the particular properties of the input data {A,b,F,g}However, in Section 7, Theorem 4 demonstrates, for the particular constraints that appear in variational elementary kinetics, each stationary point must correspond to an optimal solution where every exponential cone constraint in Problem 85 is active.

### 6.2 Convergence to stationarity

The theorem below introduces an iterative sequence of conic optimisation problems that converges to a stationary point.

#### Theorem 3.

*Let* $A\in R^{m\times n},b\in R^{m},F\in R^{3k\times n}$, *and* $g\in R^{3k}$. *Partition* F *and* g *into* v-*row blocks*

$$F=\left[\begin{array}{r} F_{1}\\ F_{2}\\ F_{3} \end{array}\right],\;g=\left[\begin{array}{r} g_{1}\\ g_{2}\\ g_{3} \end{array}\right],\;F_{i}\in R^{v\times n},g_{i}\in R^{v}(i=1,2,3).$$

*Define the convex feasible set*

(99) $$𝒳:=\left\{x\in R^{n}:A\cdot x\leq b,\;F\cdot x+g\in {𝒦}_{exp}^{v},\;F_{2}\cdot x+g_{2}=1\right\}.$$

*Assume* 𝒳 *is nonempty and compact. Define the boundary-seeking merit function* $\phi :R^{n}\to R$ *by*

(100) $$\phi (x):=1^{T}\cdot \left(\left(F_{1}x+g_{1}\right)-exp\left(F_{3}x+g_{3}\right)+ln\left(F_{1}x+g_{1}\right)-\left(F_{3}x+g_{3}\right)\right),$$

*and the major optimisation problem*

(101) $$\begin{array}{rr} \underset{x}{min} & \phi (x)\\ \text{s.t.} & x\in 𝒳, \end{array}$$

*where* ϕ(x)≥0 *and*

$$\phi (x)=0⟺F_{1}x+g_{1}=exp\left(F_{3}x+g_{3}\right).$$

*Consider the iterative scheme: choose any* $x_{0}\in 𝒳$, *and for each* k≥0 *select*

(102) $$x_{k+1}\in arg\underset{x\in 𝒳}{min}\nabla \phi {\left(x_{k}\right)}^{T}\cdot x.$$

*Then*

- ***(Concavity)*** ϕ *is concave on* 𝒳, *and* ∇ϕ(x) *exists for all* x∈𝒳.
- ***(Lyapunov descent)** The sequence* $\left\{\phi \left(x_{k}\right)\right\}$ *is nonincreasing, and with*
$$\delta_{k}:=\nabla \phi {\left(x_{k}\right)}^{T}\cdot \left(x_{k}-x_{k+1}\right)\geq 0,$$
  *one has the one-step decrease bound*
(103) $$\phi \left(x_{k+1}\right)\leq \phi \left(x_{k}\right)-\delta_{k}\;\forall k\geq 0.$$
- ***(Summability of stationarity gaps)*** $\sum_{k=0}^{\infty }\delta_{k}<\infty ,$ *hence* $\delta_{k}\to 0$.
- ***(Limit points are stationary, equivalently Variational Inequality solutions)** Every accumulation point* $x^{∙}$ *of* $\left\{x_{k}\right\}$ *is a stationary point of* Problem 101, *equivalently, it satisfies the variational inequality*

(104) $$\nabla \phi {\left(x^{∙}\right)}^{T}\cdot \left(x-x^{∙}\right)\geq 0\;\forall x\in 𝒳.$$

*Proof.* Note that $x_{k+1}$ is the optimum of Problem (73) with $c:=\nabla \phi \left(x_{k}\right)$. **Step 1: Concavity and explicit gradient.** When x∈𝒳, the constraint $F\cdot x+g\in {𝒦}_{\text{exp}}^{v}$ implies $F_{1}x+g_{1}>0$ componentwise, hence $ln\left(F_{1}x+g_{1}\right)$ and ${\left(F_{1}x+g_{1}\right)}^{-1}$ are well-defined. In (100) each summand is a sum of: a linear function of x, plus ln(⋅) composed with an affine map, plus -exp(⋅) composed with an affine map, plus another linear function of x. Since ln is concave on (0,∞) and exp is convex on R, the function − exp is concave, and composing concave functions with affine maps preserves concavity on their domains. Therefore ϕ is concave on any set where $F_{1}x+g_{1}>0$ componentwise; in particular, it is concave on 𝒳. The gradient of ϕ is

(105) $$\nabla \phi (x)=F_{1}^{T}\cdot \left(1+{\left(F_{1}x+g_{1}\right)}^{-1}\right)-F_{3}^{T}\cdot \left(exp\left(F_{3}x+g_{3}\right)+1\right).$$

For $x\in 𝒳,F_{1}x+g_{1}>0$, so ${\left(F_{1}x+g_{1}\right)}^{-1}$ is well-defined and ∇ϕ(x) exists on 𝒳.

**Step 2: Supporting hyperplane inequality for concave ϕ.** A standard consequence of concavity and differentiability is that for all x,y in the domain of ϕ,

(106) $$\phi (y)\leq \phi (x)+\nabla \phi (x{)}^{T}\cdot (y-x).$$

We will apply (106) with $x=x_{k}$ and $y=x_{k+1}$.

**Step 3: Optimality of $x_{k+1}$ in the linearised exponential-conic subproblem.** By definition (102), $x_{k+1}$ minimises the linear functional $x\mapsto \nabla \phi {\left(x_{k}\right)}^{T}\cdot x$ over 𝒳. Hence, for every x∈𝒳,

(107) $$\nabla \phi {\left(x_{k}\right)}^{T}\cdot x_{k+1}\leq \nabla \phi {\left(x_{k}\right)}^{T}\cdot x.$$

In particular, taking $x=x_{k}\in 𝒳$ gives

(108) $$\nabla \phi {\left(x_{k}\right)}^{T}\cdot \left(x_{k+1}-x_{k}\right)\leq 0.$$

Define the stationarity gap

(109) $$\delta_{k}:=\nabla \phi {\left(x_{k}\right)}^{T}\cdot \left(x_{k}-x_{k+1}\right)\geq 0,$$

which is nonnegative by (108).

**Step 4: Lyapunov descent inequality.** Apply the supporting inequality (106) with $x=x_{k}$ and $y=x_{k+1}$:

$$\phi \left(x_{k+1}\right)\leq \phi \left(x_{k}\right)+\nabla \phi {\left(x_{k}\right)}^{T}\cdot \left(x_{k+1}-x_{k}\right).$$

Using (108) (or equivalently (109)) yields

$$\phi \left(x_{k+1}\right)\leq \phi \left(x_{k}\right)-\delta_{k},$$

which is exactly (103) (cf. Figure 3). In particular, $\phi \left(x_{k+1}\right)\leq \phi \left(x_{k}\right)$, so $\left\{\phi \left(x_{k}\right)\right\}$ is nonincreasing. Thus ϕ is a (discrete-time) Lyapunov function for the iteration (102). □

*Proof.* **Step 5: Summability of** $\delta_{k}$ **and** $\delta_{k}\to 0$. Because 𝒳 is compact and ϕ is continuous on 𝒳,ϕ attains a finite lower bound on 𝒳

$$\phi_{inf}:=\underset{x\in 𝒳}{inf}\phi (x)>-\infty .$$

Sum (103) from k=0 to k=n-1:

$$\phi \left(x_{n}\right)\leq \phi \left(x_{0}\right)-\sum_{k=0}^{n-1}\delta_{k}.$$

Rearrange and use $\phi \left(x_{n}\right)\geq \phi_{\text{inf}}$:

$$\sum_{k=0}^{n-1}\delta_{k}\leq \phi \left(x_{0}\right)-\phi \left(x_{n}\right)\leq \phi \left(x_{0}\right)-\phi_{inf}.$$

Letting N→∞ shows $\sum_{k=0}^{\infty }\delta_{k}<\infty$. Since each $\delta_{k}\geq 0$, it follows that $\delta_{k}\to 0$.

**Step 6: Accumulation points satisfy the variational inequality.** Let $x^{∙}$ be any accumulation point of $\left\{x_{k}\right\}$. Since 𝒳 is compact and all iterates lie in 𝒳, there exists a subsequence $\left\{x_{k_{j}}\right\}$ such that

$$x_{k_{j}}\to x^{∙}\in 𝒳\;\text{as}\;j\to \infty .$$

We prove that $x^{∙}$ satisfies (104). Assume for contradiction that (104) fails. Then there exists $\overset{ˆ}{x}\in 𝒳$ and a scalar ε>0 such that

(110) $$\nabla \phi {\left(x^{∙}\right)}^{T}\cdot \left(\overset{ˆ}{x}-x^{∙}\right)\leq -2\varepsilon .$$

Because ∇ϕ is continuous on 𝒳 (see (105) and continuity of exp and reciprocal on $F_{1}x+g_{1}>0$, we have $\nabla \phi \left(x_{k_{j}}\right)\to \nabla \phi \left(x^{∙}\right)$. Also $x_{k_{j}}\to x^{∙}$. Therefore the scalar sequence

$$\nabla \phi {\left(x_{k_{j}}\right)}^{T}\cdot \left(\overset{ˆ}{x}-x_{k_{j}}\right)$$

converges to $\nabla \phi {\left(x^{∙}\right)}^{T}\cdot \left(\overset{ˆ}{x}-x^{∙}\right)$. Hence, for all sufficiently large j,

(111) $$\nabla \phi {\left(x_{k_{j}}\right)}^{T}\cdot \left(\overset{ˆ}{x}-x_{k_{j}}\right)\leq -\varepsilon .$$

Rearranging (111) gives

(112) $$\nabla \phi {\left(x_{k_{j}}\right)}^{T}\cdot \left(x_{k_{j}}-\overset{ˆ}{x}\right)\geq \varepsilon .$$

Now use the defining optimality property (107) for the step from $x_{k_{j}}$ to $x_{k_{j}+1}$, choosing $x=\overset{ˆ}{x}$:

$$\nabla \phi {\left(x_{k_{j}}\right)}^{T}\cdot x_{k_{j}+1}\leq \nabla \phi {\left(x_{k_{j}}\right)}^{T}\cdot \overset{ˆ}{x},$$

which is equivalent to

(113) $$\nabla \phi {\left(x_{k_{j}}\right)}^{T}\cdot \left(x_{k_{j}}-x_{k_{j}+1}\right)\geq \nabla \phi {\left(x_{k_{j}}\right)}^{T}\cdot \left(x_{k_{j}}-\overset{ˆ}{x}\right).$$

The left-hand side is $\delta_{k_{j}}$ by (109), so (113) and (112) imply

$$\delta_{k_{j}}\geq \varepsilon \;\text{for}\;\text{all}\;\text{sufficiently}\;\text{large}\;j.$$

This contradicts $\delta_{k}\to 0$ proven in Step 5. Therefore the assumption (110) was false, and $x^{∙}$ satisfies (104). Since $x^{∙}$ was an arbitrary accumulation point, every accumulation point satisfies (104). This completes the proof. □

Theorem 3 demonstrates the iteration 102 converges to a stationary point $x^{∙}$ of the merit function ϕ subject to the constraints 𝒳, defined as a solution to the variational inequality (104). Furthermore, there may exist multiple solutions to the variational inequality (104). It is a standard result in variational analysis ($6.13 in [33]) that, for any convex set 𝒳, and any mapping $f(x):𝒳\to R^{n}$, a solution $x^{∙}$ to the variational inequality (104) may be interpreted as

$$x^{∙}\in \underset{x\in 𝒳}{arg\;min}f{\left(x^{∙}\right)}^{T}\cdot x.$$

The affine function $x\to f{\left(x^{∙}\right)}^{T}\cdot x$ defines a supporting hyperplane to the convex set 𝒳 at $x^{∙}$. The halfspace $\left\{x:f{\left(x^{∙}\right)}^{T}\cdot \left(x-x^{∙}\right)\geq 0\right\}$ contains all of 𝒳 while the hyperplane $f{\left(x^{∙}\right)}^{T}\cdot \left(x-x^{∙}\right)=0$ touches 𝒳 at $x^{∙}$, so $x^{∙}$ cannot be a strict interior point of 𝒳 because an interior point admits feasible perturbations in both directions of any vector. A solution to the variational inequality (104) does not imply that any $x^{∙}$ is on the boundary of any particular combination of the constraints that define 𝒳. Equivalently, it does not imply that any particular combination of the constraints that define 𝒳 are active. Note that a stationary point may be interpreted as a fixed point of the iteration (102), where $x^{∙}:=x_{k+1}=x_{k}$.

**Figure 2:**
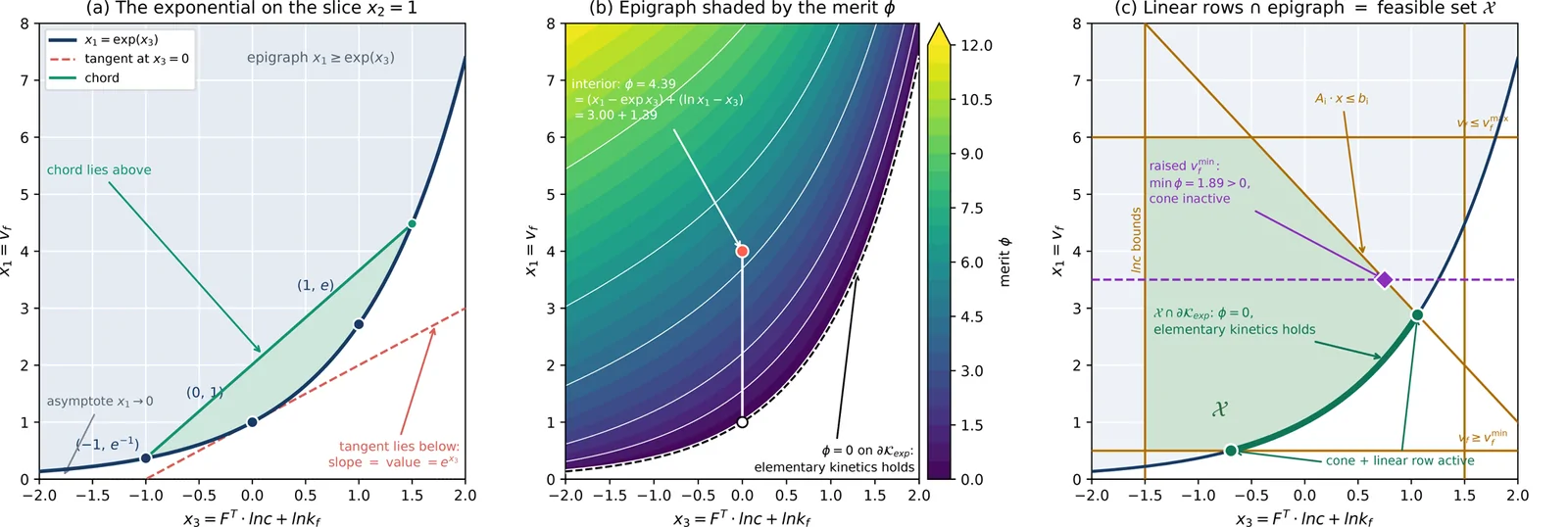
Building the variational kinetics feasible set from the exponential cone. All three panels are drawn on the two-dimensional slice $x_{2}=1$ of the exponential cone ${𝒦}_{exp}$ of (46), on which cone membership reduces to the epigraph of the exponential, $x_{1}\geq exp\left(x_{3}\right)$. For a single forward reaction the slice is read kinetically by $x_{1}=v_{f}$ and $x_{3}=F^{T}\cdot lnc+{lnk}_{f}$, so the curve $x_{1}=exp\left(x_{3}\right)$ is the elementary forward rate law (58) and the region above it is its conic relaxation $v_{f}\geq exp\left(F^{T}\cdot lnc+{lnk}_{f}\right)$ of (64). (a) The exponential, with the points $\left(-1,e^{-1}\right),(0,1)$ and (1,e) marked. Two constructions establish that the region above the curve is convex, which is what permits the relaxation to be conic at all: every chord joining two points of the curve lies above it (green), and every tangent lies below it (red, drawn at $x_{3}=0$, where the slope equals the value because exp is its own derivative). The curve approaches $x_{1}\to 0$ as $x_{3}\to -\infty$ without attaining it, the kinetic content being that the elementary rate law returns a strictly positive forward flux at every finite concentration. (b) The same epigraph, shaded by the boundaryseeking merit function (100), which for one forward reaction is $\phi =\left(v_{f}-exp\left(F^{T}\cdot lnc+{lnk}_{f}\right)\right)+\left(ln\;v_{f}-F^{T}\cdot lnc-{lnk}_{f}\right)$. It is nonnegative throughout the epigraph and vanishes exactly on the boundary $\partial {𝒦}_{exp}$ (dashed), where the rate law holds with equality. The white segment decomposes 𝜙 at the interior point $\left(x_{3},x_{1}\right)=(0,4)$ into its two additive contributions, the linear gap $v_{f}-exp\left(F^{T}\cdot lnc+{lnk}_{f}\right)=3.00$ and the logarithmic gap $ln\;v_{f}-F^{T}\cdot lnc-{lnk}_{f}=1.39$, so that ϕ=4.39; the two vanish together and only on the boundary, which is why a strictly positive ϕ certifies that at least one exponential cone constraint is inactive. Note that ϕ is strictly *concave*, its Hessian on this slice being $diag\left(-1/x_{1}^{2},-exp\left(x_{3}\right)\right)$, so minimising it is not a convex programme and its minimum over a compact convex set is attained at an extreme point of that set. (c) The feasible set 𝒳 of (99), formed as the intersection of the linear rows A⋅x≤b (orange) with the epigraph. Four rows are the box bounds any kinetic model carries, $v_{f}^{min}\leq v_{f}\leq v_{f}^{max}$, and the two bounds on lnc; the fifth, $A_{i}\cdot x\leq b_{i}$, is a general row coupling flux to the rate exponent, as a steady state row does once projected onto this slice. The bold arc is $𝒳\cap \partial {𝒦}_{exp}$, the kinetically consistent subset on which ϕ=0. It is a continuum rather than a single point, so the merit alone does not determine the solution and the remaining constraints select within it; its two endpoints, (−0.693, 0.500) and (1.059, 2.883), are points at which the exponential cone and a linear row are simultaneously active. The dashed line shows the consequence of raising $v_{f}^{min}$ until the polyhedron no longer reaches the boundary: no feasible point then satisfies elementary kinetics, and minϕ over 𝒳 is attained at the vertex marked by the diamond, (0.75, 3.50), with $\phi^{⋆}=1.89>0$ and the exponential cone inactive throughout 𝒳. This is the strictly positive merit stationary point that additional assumptions on the data {A,b,F,g} are required to exclude, and detecting it is precisely what the sign of ϕ at convergence is for.

**Figure 3:**
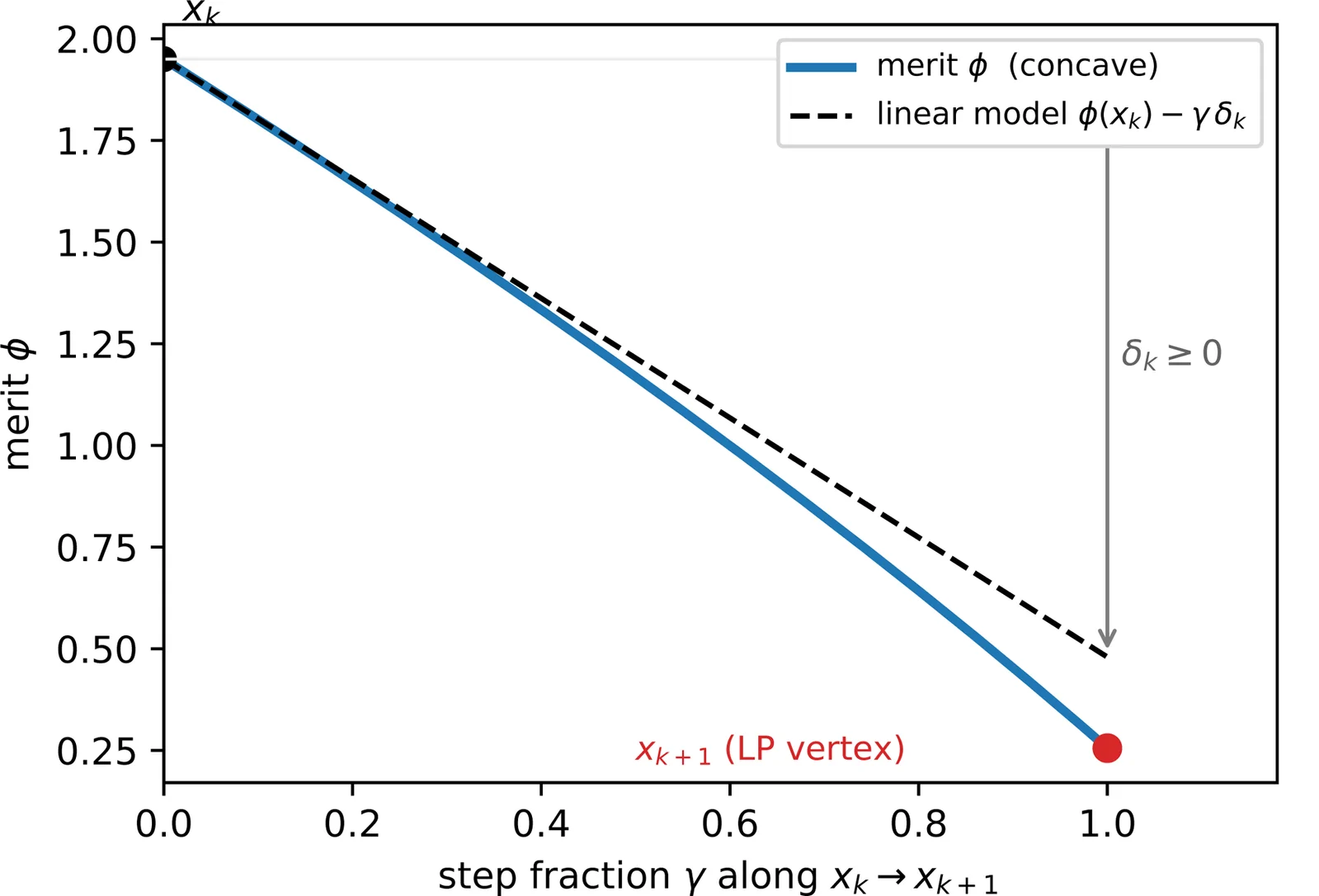
Concavity, the full step, and the guaranteed decrease underlying the convergence proof. Along the segment from the current iterate $x_{k}$ to the linear-minimisation vertex $x_{k+1}$, parameterised by the step fraction γ∈[0,1], the merit ϕ (blue) is concave, so it lies below its supporting line $\phi \left(x_{k}\right)-\gamma \delta_{k}$ (dashed), where $\delta_{k}=\nabla \phi {\left(x_{k}\right)}^{T}\cdot \left(x_{k}-x_{k+1}\right)\geq 0$ is the stationarity gap. At the full step γ=1 (reaching $x_{k+1}$) this gives $\phi \left(x_{k+1}\right)\leq \phi \left(x_{k}\right)-\delta_{k}$, the monotone decrease underlying the Lyapunov argument.

There are two types of constraints on the feasible set 99: (i) a linear constraint, defined by $A_{i}\cdot x\leq b_{i}$, that is active when $A_{i}x=b_{i}$, and, (ii) an exponential cone constraint defined by ${\left(F_{1}x+g_{1}\right)}_{i}\geq exp{\left(F_{3}x+g_{3}\right)}_{i}$, that is active when ${\left(F_{1}x+g_{1}\right)}_{i}=exp{\left(F_{3}x+g_{3}\right)}_{i}$. The merit function 100, illustrated in Figure 2, is zero when all exponential cone constraints are active and strictly positive when at least one exponential cone constraint is inactive, that is

$$\begin{array}{l} \phi (x)=0⟺\left(F_{1}x+g_{1}\right)=exp\left(F_{3}x+g_{3}\right),\\ \phi (x)>0⟺\exists \;i\in 1\ldots v,\text{s.t.}\;{\left(F_{1}x+g_{1}\right)}_{i}>exp{\left(F_{3}x+g_{3}\right)}_{i}. \end{array}$$

Therefore, a stationary point with at least one exponential cone constraint inactive (strict interior) may be recognised by a strictly positive merit function $\phi \left(x^{∙}\right)>0$. To ensure that every stationary point corresponds to activity of every exponential cone constraint, one requires additional assumptions on the input data {A,b,F,g}. That is, additional assumptions are required to eliminate the existence of a stationary point where one or more exponential cone constraints is inactive. It may be that exponential cone and linear constraints are simultaneously active. Theorem 3 proves stationarity of accumulation points, not convergence of the entire sequence to a unique point.

Supplementary Section F describes an adaptive sequential conic linear approximation algorithm that numerically implements the iterative mathematical algorithm in Theorem 3. It is but one approach to implements the iterative mathematical algorithm. It is included for completeness, underlies the numerical experiments in 10, but is agnostic to the biochemical origins that motivated it and is purely a numerical optimisation construct. The solver was developed with the assistance of AI coding tools (Claude, Anthropic; OpenAI Codex).

### 6.3 Correspondence with reaction kinetics

Theorem (3) may be applied reaction kinetics by defining the exponential boundary-seeking merit function

(114) $$\phi \left(v_{f},v_{r},lnc\right):=1^{T}\cdot \left(\left[\begin{array}{c} v_{f}\\ v_{r} \end{array}\right]-exp\left([F,R{]}^{T}\cdot lnc+\left[\begin{array}{c} {lnk}_{f}\\ {lnk}_{r} \end{array}\right]\right)+ln\left(\left[\begin{array}{c} v_{f}\\ v_{r} \end{array}\right]\right)-[F,R{]}^{T}\cdot lnc-\left[\begin{array}{c} {lnk}_{f}\\ {lnk}_{r} \end{array}\right]\right),$$

with partial derivatives

$$\begin{array}{rr} \nabla_{v_{f}}\phi & =1+1⊘v_{f}\\ \nabla_{v_{r}}\phi & =1+1⊘v_{r}\\ \nabla_{lnc}\phi & =-[F,R]\cdot \left(exp\left([F,R{]}^{T}\cdot lnc+\left[\begin{array}{c} lnk_{f}\\ lnk_{r} \end{array}\right]\right)+1\right). \end{array}$$

Theorem (3) proves that an iterative sequence of exponential conic optimisation problems each of the form of (49), generates descent of this merit function over the feasible set, with convergence to a stationary state. The correspondence between the general exponential conic optimisation problem in (49) and reaction kinetic optimisation Problem (85) is provided in Section 5.3. In particular, at the $(k+1{)}^{\text{th}}$ iteration of the iterative sequence, the linear objective coefficients in Problem (85) are

(115) $$c_{v_{f}}^{\left(k+1\right)}:=\nabla_{v_{f}}\phi =1+1⊘v_{f}^{\left(k\right)},$$

(116) $$c_{v_{r}}^{\left(k+1\right)}:=\nabla_{v_{r}}\phi =1+1⊘v_{r}^{\left(k\right)},$$

(117) $$c_{lnc}^{\left(k+1\right)}:=\nabla_{lnc}\phi =-[F,R]\cdot \left(exp\left([F,R{]}^{T}\cdot lnc^{\left(k\right)}+\left[\begin{array}{c} {lnk}_{f}\\ {lnk}_{r} \end{array}\right]\right)+1\right).$$

where $\left\{v_{f}^{\left(k\right)},v_{r}^{\left(k\right)},c_{lnc}^{\left(k\right)}\right\}$ are the optimal values of the previous minor exponential conic optimisation problem.

## 7 Variational kinetics: convergence to a steady state

The following theorem establishes sufficient conditions on the input data {F,R} such that $\left\{v_{f}^{∙},v_{r}^{∙},lnc^{∙}\right\}$ is a stationary point of the merit function ϕ subject to the constraints in Problem (85) (with b:=B⋅w), implies that elementary reaction kinetics is satisfied, that is,

$$\left[\begin{array}{c} v_{f}^{∙}\\ v_{r}^{∙} \end{array}\right]=exp\left([F,R{]}^{T}\cdot {lnc}^{∙}+\left[\begin{array}{c} {lnk}_{f}\\ {lnk}_{r} \end{array}\right]\right).$$

### Theorem 4.

*Let* $N\in R^{m\times n}$ *and* $b\in R^{m}$. *Let* $F,R\in R_{\geq 0}^{m\times n}$, *where* N=R-F, *and let the variables be* $v_{f},v_{r}\in R^{n}$ *and* $lnc\in R^{m}$. *Define the slack vectors*

$$\delta_{f}:=v_{f}-exp\left(F^{T}\cdot lnc+{lnk}_{f}\right)\geq 0,\;\delta_{r}:=v_{r}-exp\left(R^{T}\cdot lnc+{lnk}_{r}\right)\geq 0,$$

*and the sets* ${𝒮}_{4}\subseteq {𝒮}_{3}\subseteq {𝒮}_{2}\subseteq {𝒮}_{1}$ *by:*

$$\begin{array}{l} {𝒮}_{1}:=\left\{\left(v_{f},v_{r},lnc\right):N\left(v_{f}-v_{r}\right)=b,v_{f}\geq exp\left(F^{T}\cdot lnc+{lnk}_{f}\right),v_{r}\geq exp\left(R^{T}\cdot lnc+{lnk}_{r}\right),v_{f}>0,v_{r}>0\right\},\\ {𝒮}_{2}:=\left\{\left(v_{f},v_{r},lnc\right)\in {𝒮}_{1}:\exists u\in R^{m}\text{s.t.}\;sign\left(v_{f}-v_{r}\right)=-sign\left(N^{T}\cdot u\right)\right\},\\ {𝒮}_{3}:=\left\{\left(v_{f},v_{r},lnc\right)\in {𝒮}_{1}:\exists u\in R^{m}\text{s.t.}\;ln\left(v_{f}⊘v_{r}\right)=-N^{T}\cdot u⟺ln\left(v_{f}⊘v_{r}\right)\in 𝓡\left(N^{T}\right)\right\},\\ {𝒮}_{4}:=\left\{\left(v_{f},v_{r},lnc\right)\in {𝒮}_{1}:v_{f}=exp\left(F^{T}\cdot lnc+{lnk}_{f}\right),v_{r}=exp\left(R^{T}\cdot lnc+{lnk}_{r}\right)\right\}, \end{array}$$

*where* $lnc\in R^{m}$. *On the domain* $\left\{v_{f},v_{r},lnc\right\}\in {𝒮}_{1}$, *define the continuously differentiable merit function*

(118) $$\phi \left(v_{f},v_{r},lnc\right):=1^{T}\cdot \left(\left[\begin{array}{c} v_{f}\\ v_{r} \end{array}\right]-exp\left([F,R{]}^{T}\cdot lnc+\left[\begin{array}{c} {lnk}_{f}\\ {lnk}_{r} \end{array}\right]\right)+ln\left(\left[\begin{array}{c} v_{f}\\ v_{r} \end{array}\right]\right)-[F,R{]}^{T}\cdot lnc-\left[\begin{array}{c} {lnk}_{f}\\ {lnk}_{r} \end{array}\right]\right).$$

*A point* $\left(v_{f}^{∙},v_{r}^{∙}\right.,{lnc}^{∙}\left.\right)\in {𝒮}_{1}$ *is called* a first-order stationary point of ϕ over ${𝒮}_{1}$ *if, for every direction* $\left(d_{f},d_{r},d_{c}\right)\in R^{2n+m}$ *for which there exists* ϵ>0 *such that*

$$\left(v_{f}^{∙}+\alpha d_{f},v_{r}^{∙}+\alpha d_{r},{lnc}^{∙}+\alpha d_{c}\right)\in {𝒮}_{1},\;\forall \alpha \in [0,\epsilon ],$$

*the directional derivative is non-negative, that is*

(119) $$\nabla \phi {\left(v_{f}^{∙},v_{r}^{∙},{lnc}^{∙}\right)}^{T}\cdot \left[\begin{array}{r} d_{f}\\ d_{r}\\ d_{c} \end{array}\right]\geq 0.$$

*Assume the data satisfy the following:*

*(2.1)* ***(Independent forward and reverse stoichiometry)** For every* j∈{1,…,n},

(120) $$supp\left(F_{:,j}\right)\cap supp\left(R_{:,j}\right)=\emptyset ,\;F_{:,j}\neq 0,R_{:,j}\neq 0.$$

*(2.2) **(Cyclic flux consistency)** Every reaction participates in at least one stoichiometrically balanced cycle. That is, for every* j, *there exists a* $z\in R^{n}$ *such that*

(121) $$Nz=0,\;z_{j}\neq 0.$$

*Then every first-order stationary point* $\left(v_{f}^{∙},v_{r}^{∙},{lnc}^{∙}\right)\in {𝒮}_{1}$ *lies in* ${𝒮}_{4}$. *Equivalently*,

$$\left[\begin{array}{c} v_{f}^{∙}\\ v_{r}^{∙} \end{array}\right]=exp\left([F,R{]}^{T}\cdot {lnc}^{∙}+\left[\begin{array}{c} {lnk}_{f}\\ {lnk}_{r} \end{array}\right]\right).$$

*so there is no bidirectional slack and no unidirectional slack in the kinetic inequalities at* $\left(v_{f}^{∙},v_{r}^{∙},{lnc}^{∙}\right)$.

*Proof.* We prove three claims: **Claim 1:** No stationary point lies in ${𝒮}_{1}\setminus {𝒮}_{2}$. **Claim 2:** No stationary point lies in ${𝒮}_{2}\setminus {𝒮}_{3}$. **Claim 3:** No stationary point lies in ${𝒮}_{3}\setminus {𝒮}_{4}$. Since ${𝒮}_{4}\subseteq {𝒮}_{3}\subseteq {𝒮}_{2}\subseteq {𝒮}_{1}$, these claims imply any stationary point in ${𝒮}_{1}$ must lie in ${𝒮}_{4}$. To rule out stationary of the merit function (118) at a point $\left(v_{f},v_{r}\right.,lnc)$, it suffices to establish the existence of one feasible direction with strictly negative directional derivative (119). All stationary statements are made with respect to the feasible set ${𝒮}_{1}$, even if it leaves ${𝒮}_{2}$ or ${𝒮}_{3}$. Thus, in each claim it suffices to construct a descent direction that remains feasible in ${𝒮}_{1}$. For clarity, henceforth in this section we omit the constants $k_{f},k_{r}$. **Preliminaries (first order feasibility for kinetic inequalities).** For each j, in the forward direction, if

(122) $$v_{f}(j)=exp\left(F_{:,j}^{T}\cdot lnc\right)$$

then we require the direction $d_{c}$ to satisfy $v_{f}(j)\geq exp\left(F_{:,j}^{T}\cdot lnc\right)$ for a small step $\alpha d_{c}$, where α>0, that is

(123) $$v_{f}(j)+\alpha d_{f}(j)\geq exp\left(F_{:,j}^{T}\cdot \left(lnc+\alpha d_{c}\right)\right).$$

The first-order expansion of $exp\left(F_{:,j}^{T}\cdot lnc\right)$ is

$$exp\left(F_{:,j}^{T}\cdot \left(lnc+\alpha d_{c}\right)\right)=exp\left(F_{:,j}^{T}\cdot lnc\right)\left(1+\alpha F_{:,j}^{T}\cdot d_{c}+o(\alpha )\right).$$

where o(α) denotes a remainder satisfying o(α)/α→0 as α↓0. Adding $\alpha d_{f}(j)$ and then subtracting $exp\left(F_{:,j}^{T}\right.\cdot \left.\left(lnc+\alpha d_{c}\right)\right)$ from 122, we have

$$v_{f}(j)+\alpha d_{f}(j)-exp\left(F_{:,j}^{T}\cdot \left(lnc+\alpha d_{c}\right)\right)=exp\left(F_{:,j}^{T}\cdot lnc\right)+\alpha d_{f}(j)-exp\left(F_{:,j}^{T}\cdot lnc\right)\left(1+\alpha F_{:,j}^{T}\cdot d_{c}+o(\alpha )\right),\;\;\;\;\;\;\;\;\;\;\;\;\;=\alpha \left(d_{f}(j)-exp\left(F_{:,j}^{T}\cdot lnc\right)\left(F_{:,j}^{T}\cdot d_{c}\right)\right)+o(\alpha ).$$

Thus a sufficient first-order condition to ensure 123 holds for sufficiently small α is

(124) $$d_{f}(j)-exp\left(F_{:,j}^{T}\cdot lnc\right)\left(F_{:,j}^{T}\cdot d_{c}\right)\geq 0.$$

Similarly, for a reverse direction, if $v_{r}(j)=exp\left(R_{:,j}^{T}\cdot lnc\right)$, a sufficient first-order condition to ensure $v_{r}(j)+\alpha d_{r}(j)\geq exp\left(R_{:,j}^{T}\cdot \left(lnc+\alpha d_{c}\right)\right)$ for a sufficiently small α is

(125) $$d_{r}(j)-exp\left(R_{:,j}^{T}\cdot lnc\right)\left(R_{:,j}^{T}\cdot d_{c}\right)\geq 0.$$

Slack forward or reverse directions impose no restriction on $d_{c}$. **Gradient sign facts.** Since $\nabla_{v_{f}}\phi =1+v_{f}^{-1}>0$ and $\nabla_{v_{r}}\phi =1+v_{r}^{-1}>0$, for any $d_{f}\leq 0,d_{r}\leq 0$ with $\left(d_{f},d_{r}\right)\neq 0$,

$${\left(\nabla_{v_{f}}\phi \right)}^{T}\cdot d_{f}+{\left(\nabla_{v_{r}}\phi \right)}^{T}\cdot d_{r}<0.$$

**Justification of Claim 1.** Take any $\left(v_{f},v_{r},lnc\right)\in {𝒮}_{1}\setminus {𝒮}_{2}$ and orthogonally decompose the net flux as $v_{f}-v_{r}=v+z$ with $v\in 𝓡\left(N^{T}\right),z\in 𝒩(N)$, so Nv=b and Nz=0. If z=0 then $v_{f}-v_{r}=N^{T}\cdot \overset{‾}{u}$ for some $\overset{‾}{u}$; with $u:=-\overset{‾}{u}$ we get $sign\left(v_{f}-v_{r}\right)=-sign\left(N^{T}\cdot u\right)$, which is the membership condition for ${𝒮}_{2}$, contradicting $\left(v_{f},v_{r},lnc\right)\in {𝒮}_{1}\setminus {𝒮}_{2}$. Hence z≠0. Decompose 𝑧 as follows:

(126) $$z=z_{f}-z_{r},\;z_{f}\geq 0,z_{r}\geq 0,\;z_{f}⊙z_{r}=0,$$

so $z_{f}$ and $z_{r}$ are non-negative and have disjoint support. Set $d_{f}:=-z_{f},d_{r}:=-z_{r}$. Since z≠0 and $z_{f},z_{r}$ have disjoint support, $\left(d_{f},d_{r}\right)\neq 0$; moreover $N\left(d_{f}-d_{r}\right)=N(-z)=-Nz=0$, so the steady-state equality $N\left(v_{f}-v_{r}\right)=b$ is preserved to first order. The log-concentration lnc does not appear in this equality, so its direction $d_{c}$ is unconstrained by it: rescaling $d_{c}\mapsto \lambda d_{c}$ leaves $N\left(d_{f}-d_{r}\right)=0$ intact and $d_{c}$ enters only the exponential cone tightness conditions, which are positively homogeneous in $d_{c}$. Hence there is ϵ>0 with $\left(v_{f}+\alpha d_{f},v_{r}+\alpha d_{r},lnc+\alpha d_{c}\right)\in {𝒮}_{1}$ for α∈[0,ϵ], and scaling $d_{c}$ then the whole direction by τ∈(0,1) yields $\nabla \phi {\left(v_{f},v_{r},lnc\right)}^{T}\cdot \left(d_{f},d_{r},d_{c}\right)<0$, the strictness using $\left(d_{f},d_{r}\right)\neq 0$. This contradicts stationarity, so no stationary point lies in ${𝒮}_{1}\setminus {𝒮}_{2}$. **Claim 2.** Take any $\left(v_{f},v_{r}\right.,lnc)\in {𝒮}_{2}\setminus {𝒮}_{3}$. By the characterisation of ${𝒮}_{3}$ this membership gives $ln\left(v_{f}🚫v_{r}\right)\notin 𝓡\left(N^{T}\right)=𝒩(N{)}^{⊥}$, so the component of $ln\left(v_{f}🚫v_{r}\right)$ in 𝒩(N) is nonzero: for any Z whose columns span 𝒩(N) (so $NZ=0),Z^{T}\cdot ln\left(v_{f}🚫v_{r}\right)\neq 0$. Hence there is z∈𝒩(N) with $z_{j}\neq 0$ for some j; in particular z≠0. Split $z=z_{f}-z_{r}$ as in 126 and set $d_{f}:=-z_{f},d_{r}:=-z_{r}$. As in Claim 1, $\left(d_{f},d_{r}\right)=\left(-z_{f},-z_{r}\right)\neq 0$ and $N\left(d_{f}-d_{r}\right)=-Nz=0$, so the direction is nonzero and preserves the steady-state equality to first order. Choosing $d_{c}$ as in Claim 1 so the exponential cone tightness conditions (124)–(125) hold with slack gives ϵ>0 with the perturbed point in ${𝒮}_{1}$ for α∈[0,ϵ], and rescaling $d_{c}$ yields $\nabla \phi^{T}\cdot \left(d_{f},d_{r},d_{c}\right)<0$. The strict inequality requires $\left(d_{f},d_{r}\right)\neq 0$: the first-order descent is carried by the flux terms, whose contribution is proportional to $\left(d_{f},d_{r}\right)$ and would vanish if $\left(d_{f},d_{r}\right)=0$; nonzeroness is exactly what the ${𝒮}_{2}\setminus {𝒮}_{3}$ membership provides. This contradicts stationarity, so no stationary point lies in ${𝒮}_{2}\setminus {𝒮}_{3}$.

**Claim 3.** Take any $\left(v_{f},v_{r},lnc\right)\in {𝒮}_{3}\setminus {𝒮}_{4}$. Then $\delta_{f}\neq 0$ or $\delta_{r}\neq 0$, so there exists at least one index j such that either

$$v_{f}(j)>exp\left(F_{:,j}^{T}\cdot (lnc)\right)\;\text{or}\;v_{r}(j)>exp\left(R_{:,j}^{T}\cdot (lnc)\right).$$

We treat the case $\delta_{r}\neq 0⟺v_{r}(j)>exp\left(R_{:,j}^{T}\cdot (lnc)\right)$; the other case is symmetric. Because $v_{r}(j)$ is strictly above $exp\left(R_{:,j}^{T}\cdot (lnc)\right)$, there exists ϵ>0 such that for all α∈(0,ϵ],

$$v_{r}(j)-\alpha \geq exp\left(R_{:,j}^{T}\cdot (lnc)\right).$$

Thus decreasing $v_{r}(j)$ slightly does not violate the reverse inequality at index j. By (121), choose z∈𝒩(N) and $z_{j}\neq 0$. If necessary replace z←-z so that $z_{j}<0$. Decompose z as in 126 and define

$$d_{f}:=-z_{f},\;d_{r}:=-z_{r}.$$

Then $N\left(d_{f}-d_{r}\right)=0$, so the equality constraint is preserved to first order. Since $z_{j}<0$ then $z_{r}(j)=-z_{j}>0$ so $d_{r}(j)=-z_{r}(j)<0$ decreases $v_{r}(j)$. Choose $d_{c}$ as in Claim 1 so that (124)–(125) hold on the tight sets, ensuring feasibility in ${𝒮}_{1}$ for small steps. As in Claim 1, scaling $d_{c}$ if necessary yields a feasible direction with

$$\nabla \phi {\left(v_{f},v_{r},lnc\right)}^{T}\cdot \left[\begin{array}{r} d_{f}\\ d_{r}\\ d_{c} \end{array}\right]<0,$$

contradicting stationarity. Hence no stationary point lies in ${𝒮}_{3}\setminus {𝒮}_{4}$. Combining Claims 1–3, every first-order stationary point $\left(v_{f}^{∙},v_{r}^{∙},{lnc}^{∙}\right)\in {𝒮}_{1}$ must lie in ${𝒮}_{4}$, so all kinetic inequalities are tight, that is

$$v_{f}^{∙}=exp\left(F^{T}\cdot {lnc}^{∙}\right),\;v_{r}^{∙}=exp\left(R^{T}\cdot {lnc}^{∙}\right).$$

□

#### 7.1 Interpretation of Theorem (4).

The right hand side of both kinetic inequalities $v_{f}\geq exp\left(F^{T}\cdot lnc+{lnk}_{f}\right)$ and $v_{r}\geq exp\left(R^{T}\cdot lnc+{lnk}_{r}\right)$ are lower bounds on each one-way rate implied by elementary kinetics. The slack vectors $\delta_{f}$ and $\delta_{r}$ therefore quantify the extent to which the chosen rates are *in excess* of what is kinetically implied by lnc. In biochemical terms, nonzero slack corresponds to a “rate assignment” that cannot be attributed to the stated substrate/product dependencies alone (e.g., it would implicitly require unmodelled activation, inhibition, regulation, or additional species).

The merit function ϕ is a separable barrier-like penalty that strictly prefers smaller positive one-way fluxes while also penalising positive slack vectors. Its gradient with respect to $v_{f}$ and $v_{r}$ is strictly positive componentwise; consequently, any feasible perturbation that decreases any component of $v_{f}$ or $v_{r}$ produces an immediate decrease in the merit value, unless doing so violates feasibility. The proof leverages this fact by explicitly constructing feasible “rate-reducing” directions and showing that, unless all kinetic inequalities are tight, such a direction always exists.

The three-stage argument admits a direct biochemical interpretation. In ${𝒮}_{1}\setminus {𝒮}_{2}$, the net flux vector $v_{f}-v_{r}$ contains a component lying in the internal flux cycle space 𝒩(N). Such cycle flux can circulate without changing the external exchange balance 𝑏 [6]. The proof shows that whenever such a cyclic component is present, one can reduce a subset of one-way rates along the cycle while maintaining the steady-state balance $N\left(v_{f}-v_{r}\right)=b$. Because ϕ strictly decreases when one-way rates decrease, any point with a removable cyclic component cannot be stationary. This corresponds to the biochemical notion that purely internal futile cycling is disfavoured by the merit: it is “unproductive” with respect to meeting the exchange demands b, yet it increases one-way turnover. ${𝒮}_{1}$ corresponds to the feasible set of Problem 30. ${𝒮}_{2}$ corresponds to the set of optimal solutions to Problem 32.

In ${𝒮}_{2}\setminus {𝒮}_{3}$, the natural log ratio $ln\left(v_{f}🚫v_{r}\right)$ is not compatible with a potential-like representation in the stoichiometric row space. Biochemically, $ln\left(v_{f}🚫v_{r}\right)$ plays the role of a force term (affinity-like quantity) driving the net direction of each reaction; requiring $ln\left(v_{f}🚫v_{r}\right)\in 𝓡\left(N^{T}\right)$ enforces that these forces are consistent with a globally defined set of chemical potentials. The proof shows that if this compatibility fails, then there again exists a nullspace direction along which one can decrease one-way rates without affecting the steady state constraints, contradicting stationarity. ${𝒮}_{3}$ corresponds to optimality condition 36 in one of the optimality conditions of Problem 37, which requires $ln\left(v_{f}⊘v_{r}\right)\in 𝓡\left(N^{T}\right)$.

Finally, in ${𝒮}_{3}\setminus {𝒮}_{4}$, the system is already “potential-consistent” (the ln forward/reverse ratios can be written as $-N^{T}\cdot u$), but at least one kinetic inequality is slack. In biochemical terms, this means that even though the directionality pattern is consistent with a global potential, at least one reaction has an excess one-way rate beyond what the concentrations would imply. The cyclic flux consistency assumption ensures that for any reaction index j exhibiting slack, there exists an internal cycle that includes reaction j. This guarantees a feasible cycle-based perturbation that reduces the slack one-way rate at j while preserving the net exchanges b. Hence slack cannot persist at stationarity: the only stationary configurations are those in which every one-way rate is exactly matched to the monomial kinetics implied by lnc, i.e., $\delta_{f}=\delta_{r}=0$. The innermost set ${𝒮}_{4}$ is the set of elementary kinetic steady states, in which both kinetic inequalities hold with equality (1, 2); it is the feasible set of the variational kinetics Problem 69, over which the algorithm optimises.

The proof is structured in stages because different types of non-physical or non-minimal behaviour arise from distinct geometric features of the feasible set. Internal cycling $\left({𝒮}_{1}\setminus {𝒮}_{2}\right)$, thermodynamic inconsistency $\left({𝒮}_{2}\setminus {𝒮}_{3}\right)$, and kinetic slack $\left({𝒮}_{3}\setminus {𝒮}_{4}\right)$ correspond to progressively stronger notions of feasibility that may be difficult to be ruled out by a single argument. Each stage identifies one source of excess in the merit function and constructs a descent direction tailored to that mechanism, while maintaining feasibility with respect to all constraints. This staged approach mirrors a biochemical hierarchy from mass-balance yet net flux direction inconsistent with thermodynamics $\left({𝒮}_{1}\setminus {𝒮}_{2}\right)$, to steady state and net flux direction consistent with thermodynamics, yet inconsistent with the ratio of forward over reverse unidirectional fluxes $\left({𝒮}_{2}\setminus {𝒮}_{3}\right)$, to steady state and full thermodynamic consistency yet kinetic inconsistency $\left({𝒮}_{3}\setminus {𝒮}_{4}\right)$, and makes explicit how the merit function eliminates each form of inconsistency in turn.

Overall, the proof formalises the following biochemical principle: if a flux configuration contains any removable internal cycling or any excess one-way turnover beyond that implied by concentrations, then the merit function provides a direction of feasible improvement that reduces total one-way turnover while maintaining the same external demands. Under assumptions (120)–(121), the only points where this is no longer possible are those where all kinetic inequalities are tight, meaning the rates are fully explained by the kinetic monomials for the same lnc. This justifies the method as a constructive mechanism for eliminating futile internal cycling and enforcing kinetically coherent one-way rates consistent with steady-state exchange requirements.

At a stationary point of Theorem (3) the objective in Problem (85) is

$$\begin{array}{rr} c_{v_{f}}^{T}\cdot v_{f}^{∙} & :={\left(1+1⊘v_{f}^{∙}\right)}^{T}\cdot v_{f}^{∙}=1^{T}\cdot v_{f}^{∙}+n\\ c_{v_{r}}^{T}\cdot v_{r}^{∙} & :={\left(1+1⊘v_{r}^{∙}\right)}^{T}\cdot v_{r}^{∙}=1^{T}\cdot v_{r}^{∙}+n\\ c_{lnc}^{T}\cdot {lnc}^{∙} & :=-{\left([F,R]\cdot exp\left([F,R{]}^{T}\cdot {lnc}^{∙}+\left[\begin{array}{c} {lnk}_{f}\\ {lnk}_{r} \end{array}\right]\right)+1\right)}^{T}\cdot {lnc}^{∙}=\nabla \phi {\left({lnc}^{∙}\right)}^{T}\cdot {lnc}^{∙} \end{array}$$

which may be interpreted as minimising the sum of unidirectional fluxes and maximising the rate of consumption of every species weighted by the logarithm of species concentration.

## 8 Additional constraints and regularisation

Section 5, 6 and 7 establish convergence properties for an algorithm to obtain a steady state, as defined by Eq. 9. Beyond that, there are additional constraints that can be added, such as moiety conservation and thermodynamic constraints on kinetic parameters. Strictly, Theorem 4 applies to solutions to Eq. 9, but in practice, addition of the constraints below is observed to be numerically compatible with convergence to satisfaction of elementary kinetics also.

### 8.1 Moiety conservation constraints

The moiety conservation constraints in Eq. (8) are linear in linear concentrations, while the formulation of variational elementary kinetics in Problem (69) is expressed in terms of logarithmic concentrations, therefore to add moiety conservation to Problem (69), one can employ an exponential cone to constrain the relationship between linear and logarithmic concentration variables then add terms to optimise to the boundary of this cone, to give the conic optimisation problem

(127) $$\begin{array}{cc} \underset{c,lnc}{min} & c_{c}^{T}\cdot c+c_{lnc}^{T}\cdot lnc\\ \text{s.t.} & L\cdot c=L\cdot c(0), \end{array}$$

(128) $$\left(\begin{array}{c} c\\ 1\\ lnc \end{array}\right)\in {𝒦}_{exp}^{m},$$

where at an optimum we have $c_{i}^{⋆}=exp\left({lnc}_{i}^{⋆}\right)$ when $c_{i}$ and ${lnc}_{i}$ lie on the exponential face of the $i^{\text{th}}$ exponential cone.

### 8.2 Thermodynamic constraints on elementary kinetic parameters

In an extension to (69), logarithmic forward and reverse kinetic parameters may be modelled as variables, in which case it is possible to implement thermodynamic constraints on kinetic parameters using

(129) $${lnk}_{f}-{lnk}_{r}+N^{T}\cdot u^{\circ }=0,$$

which is linear system of equations in logarithmic variables.

### 8.3 Regularisation of kinetic steady states

Assuming a solution exists to (9) implies that there exists an optimal solution to (69), where each exponential cone constraint is active, that is, the optimal solution is on the exponential face of each exponential cone. However, it may occur that there does not exist a steady state solution to (7) that also satisfies the elementary reaction rate laws (52) and (53), as well as box constraints on internal and external reaction rates. As described in 3.5, replacement of stoichiometrically inconsistent exchange reactions with perpetireactions, that are stoichiometrically consistent but driven by intentionally thermodynamically infeasible kinetic parameters, is the principled and theoretically supported approach to ensure there exists a non-equilibrium steady state. However, in practice there are a large cadre of established modes that employ exchange reactions so it is useful to have the option to solve for a regularised steady state solution that penalises deviation from steady state in case the given exchange reactions are not compatible with a kinetically feasible steady state. This can be achieved by the addition of a quadratic penalty on a regularisation variable, $r\in R^{m}$, which is conically representable with a rotated quadratic cone, that is

(130) $$\begin{array}{r} \underset{v_{f},v_{r},w,r,t_{r},}{min}\;c_{t_{r}}\cdot t_{r}\\ \text{s.t.}\;N\cdot \left(v_{f}-v_{r}\right)+r+B\cdot w=0, \end{array}$$

(131) $$\left(\begin{array}{c} t_{r}\\ 1\\ H_{r}\cdot r \end{array}\right)\in Q^{2+m}.$$

where $t_{r}\in R_{\geq 0}$ is an auxiliary variable. Penalisation can be weighted for or against different molecular species by selection of a suitable set of weights on the diagonal of $H_{r}\in R^{m\times m}$, but the default is $H_{r}:=I$, Regularisation enables one to relax one or more steady state constraints in (7) yet still satisfy elementary reaction kinetics, in the case where a solution to (9) does not exist.

### 8.4 Optimisation of network states

A key feature of constraint-based modelling is the ability to optimise over a feasible set of network states. In flux balance analysis, such optimisation is over a feasible set of steady state fluxes. Typically, optimisation is of one or more exchange fluxes, rather than internal fluxes. Although optimisation over any linear combination of internal and external fluxes is possible, internal fluxes with support in the nullspace of the internal stoichiometric matrix must be bounded by given lower or upper bounds to avoid an unbounded optimisation problem, if any of those reactions is optimised. In entropic flux balance analysis [8], every thermodynamically feasible steady state net flux can be obtained as a function of parameters corresponding to internal reactions that may be interpreted as prior information in a relative entropy optimisation problem [9]. Entropic flux balance analysis can also include optimisation of any linear combination of external net fluxes, via a trade off between (relative) entropy optimisation of internal fluxes and optimisation of exchange fluxes. However, this is not optimisation *over* a set of thermodynamically feasible fluxes, because that set is non-convex. In variational kinetics, it is currently not possible to arbitrarily optimise internal net fluxes, concentrations, or kinetic parameters, without interfering with convergence to satisfaction of elementary reaction kinetics, because it is the objective coefficients corresponding to unidirectional fluxes, concentrations (± kinetic parameters) that are iteratively optimised to ensure that elementary reaction kinetics is satisfied. However, one can optimise over the set of net external fluxes as in flux balance analysis, by adding a linear objective over exchange reactions, that is $c_{w}^{T}\cdot w$. In practice, in each conic optimisation problem in the terms in the linear objective compete with one another, therefore it is beneficial to add scalar parameter to balance optimisation of exchange fluxes with satisfaction of kinetics, that is \alpha_{w}c_{w}^{T}\cdot w.

each term in the linear objective. Therefore, to establish priorities between the different objectives within the combined formulation of variational elementary kinetics, we introduce non-negative scalar weights, denoted $\alpha_{x}\in R_{\geq 0}$ where the subscript x is replaced by a symbol that corresponds to the primal variable. That is, the objective in the combined formulation of variational elementary kinetics becomes

(132) $$min\;\alpha_{v_{f}}c_{v_{f}}^{T}\cdot v_{f}+\alpha_{v_{r}}c_{v_{r}}^{T}\cdot v_{r}+\alpha_{{lnk}_{f}}c_{lnk_{f}}^{T}\cdot {lnk}_{f}+\alpha_{lnk_{r}}c_{lnk_{r}}^{T}\cdot {lnk}_{r}\ldots$$

(133) $$+\alpha_{lnc}c_{lnc}^{T}\cdot lnc\ldots +\alpha_{c}c_{c}^{T}\cdot c\ldots$$

(134) $$+\alpha_{t_{r}}c_{t_{r}}^{T}\cdot t_{r}\ldots$$

(135) $$+\alpha_{w}c_{w}^{T}\cdot w.$$

The relative values of these scalar weights substantially affects the type of variational kinetic solution obtained.

## 9 Variational elementary kinetics

In this section, each of the conic optimisation problems in the preceding sections is combined into a single conic optimisation problem. Each constraint is represented with the corresponding dual variables, where y denotes a dual variable to a linear equality constraint, s denotes a dual variable to a cone constraint and z denotes a dual variable to a box constraint. The subscript to these dual variables is chosen to reflect a correspondence to a primal term. The combined formulation of variational elementary kinetics is

(136) $$min\;c_{v_{f}}^{T}\cdot v_{f}+c_{v_{r}}^{T}\cdot v_{r}+c_{lnc}^{T}\cdot lnc+c_{lnk_{f}}^{T}\cdot {lnk}_{f}+c_{lnk_{r}}^{T}\cdot {lnk}_{r}\ldots$$

(137) $$+c_{c}^{T}\cdot c\ldots$$

(138) $$+c_{t_{r}}^{T}\cdot t_{r}\ldots$$

(139) $$+c_{w}^{T}\cdot w$$

(140) $$\text{s.t.}\;N\cdot \left(v_{f}-v_{r}\right)+r+B\cdot w=0:y_{N}$$

(141) $${lnk}_{f}-{lnk}_{r}+N^{T}\cdot u^{\circ }=0:y_{u^{\circ }}$$

(142) $$L\cdot c=L\cdot c(0):y_{L}$$

(143) $$\left(\begin{array}{c} v_{f}\\ 1\\ F^{T}\cdot lnc+{lnk}_{f} \end{array}\right)\in {𝒦}_{exp}^{n}:\left(\begin{array}{c} s_{vf}\\ s_{f1}\\ s_{F} \end{array}\right),$$

(144) $$\left(\begin{array}{c} v_{r}\\ 1\\ R^{T}\cdot lnc+{lnk}_{r} \end{array}\right)\in {𝒦}_{exp}^{n}:\left(\begin{array}{c} s_{vr}\\ s_{r1}\\ s_{R} \end{array}\right),$$

(145) $$\left(\begin{array}{c} c\\ 1\\ lnc \end{array}\right)\in {𝒦}_{exp}^{m}:\left(\begin{array}{c} s_{c}\\ s_{c1}\\ s_{lnc} \end{array}\right),$$

(146) $$\left(\begin{array}{c} t_{r}\\ 1\\ H_{r}\cdot r \end{array}\right)\in {𝒬}^{2+m}:\left(\begin{array}{c} s_{t_{r}}\\ s_{r1}\\ s_{H_{r}} \end{array}\right).$$

The following are the intent of the terms in the objective. The linear coefficients in the terms (136) are iteratively updated to optimise fluxes, logarithmic concentrations, and logarithmic kinetic parameters (unless they are fixed) so the corresponding exponential cone constraints representing elementary kinetics are active at a stationary state (143). The linear coefficients in the term (137) is iteratively updated to so the corresponding exponential cone constraints ((143),144,145) are active at a stationary state and therefore $c^{∙}=exp\left({lnc}^{∙}\right)$. The term (138) implements regularisation of steady state constraints. The term (139) implements linear optimisation of net external reaction flux. The following are the intent of the equality constraints. Equation (140) implements regularised steady state, in conjunction with the term (138) and the rotated quadratic cone (146). Equation (141) implements thermodynamic constraints on logarithmic elementary kinetic parameters. Finally, Eq. (142) implements moiety conservation.

When implementing this conic optimisation problem numerically, one must encode upper and lower bounds for each variable, but they may be specified to be unbounded, except in the case where they are required to be fixed to be a certain given value specified as prior data, or where an unbounded variable may result in an unbounded optimisation problem, e.g., upper bounds on elementary fluxes and lower bounds on logarithmic concentrations. The following additional inequality constraints are used to constrain unidirectional fluxes, net internal reaction fluxes, net exchange reaction fluxes, logarithmic concentrations, logarithmic kinetic parameters and logarithmic standard Gibbs energies of formation

(147) $$v_{f}\leq u_{v_{f}}\;:-z_{v_{f}},$$

(148) $$v_{r}\leq u_{v_{r}}\;:-z_{v_{r}},$$

(149) $$l_{v}\leq v_{f}-v_{r}\leq u_{v}\;:z_{v}:=z_{lv}-z_{uv},$$

(150) $$l_{w}\leq w\leq u_{w}\;:z_{w}:=z_{lw}-z_{uw},$$

(151) $$l_{lnc}\leq lnc\leq u_{lnc}\;:z_{lnc}:=z_{llnc}-z_{ulnc},$$

(152) $$l_{lnk_{f}}\leq {lnk}_{f}\leq u_{lnk_{f}}\;:z_{lnk_{f}}:=z_{llnk_{f}}-z_{ulnk_{f}},$$

(153) $$l_{lnk_{r}}\leq lnk_{r}\leq u_{lnk_{r}}\;:z_{lnk_{r}}:=z_{llnk_{r}}-z_{ulnk_{r}},$$

(154) $$l_{u^{\circ }}\leq u^{\circ }\leq u_{u^{\circ }}\;:z_{u^{\circ }}:=z_{lu^{\circ }}-z_{uu^{\circ }}.$$

For each box constraint, a net dual vector and two non-negative dual vectors corresponding to lower and upper bound constraints are introduced, with dimensions corresponding to the primal variable concerned, e.g., $l_{v}\in R^{n}$ and $u_{v}\in R^{n}$ denote lower and upper bounds on net flux $v_{f}-v_{r},z_{lv}\in R^{n}$ denotes a non-negative dual vector to the lower bound constraint and $z_{uv}\in R^{n}$ denotes a non-negative dual vector to the upper bound constraint, with the dual vector corresponding to box constraints on net flux defined as the difference between these two vectors, that is $z_{v}:=z_{lv}-z_{uv}$. The upper bounds on the unidirectional fluxes are one-sided, so each introduces a single non-negative dual vector, $z_{v_{f}},z_{v_{r}}\in R_{\geq 0}^{n}$, rather than a net dual vector. The Lagrangian corresponding to the combined formulation of variational kinetics expressed as Problem (136)–(154) is provided in Supplementary Section 9.

## 10 Numerical experiments

Numerical experiments are presented as a rendered computational narrative (Supplementary_File_1.html https://doi.org/10.5281/zenodo.21633862) generated by the MATLAB R2024b (Mathworks Inc.) numerical computing environment, using the COBRA Toolbox (v3.8beta, specifically SHA-1 shorthand: 4713424eb) [39] that accesses an implementation of the sequential conic solver https://doi.org/10.5281/zenodo.21633862 https://github.com/Digital-Metabolic-Twin-Centre/varkin (SHA-1 shorthand: b130be3) and an industrial quality conic optimisation solver (MOSEK Version 11.2.0, feasibility and optimality tolerance 1*e* − 6), on a workstation (x86_64, Intel(R) Core(TM) i9–10980XE CPU @ 3.00GHz) running a Linux operating system (Linux 6.17.0–35-generic #35~24.04.1-Ubuntu). The computational narrative can be applied to a variety of genomescale metabolic models but in this section, results are summarised for experiments with a stoichiometrically, flux and thermodynamically flux consistent subset [35, 37] of a generic human genome-scale metabolic model (Recon3, [40]), containing 5,835 metabolites and 8,791 internal reactions.

Supplementary File 1 contains three numerical experiments. The three experiments differ in what boundary condition b is and what extra constraints are imposed. Experiment 1 (VK1) — satisfaction of elementary kinetics. The plainest case: find a concentration vector consistent with the elementary kinetics at steady state, kinetic parameters fixed at their given values, no synthetic target to recover and no moiety conservation. Constraints are the exponential cone kinetics, the hard steady state equality, and the concentration/flux bounds; all penalties off. It converged trivially a single major iteration with a clean conic certificate (less than kinetic equality tolerance tolEXP:=5e-5).

Experiment 2 (VK2) — recover a steady state from a known boundary condition. Here a random, thermodynamically feasible kinetic steady state is generated, from a random concentration vector and fixed kinetic parameters, ${lnk}_{f}={lnk}_{r}=0$, the corresponding boundary flux $b=N\cdot \left(exp\left(ln\left(k_{f}\right)+F^{T}\cdot ln(c)\right)-exp\left(ln\left(k_{r}\right)+\right.\right.\left.R^{T}\cdot ln(c)\right))$ is computed from and the solver must find a steady state satisfying that same b (with kinetic parameters fixed, but no knowledge of the generating c). This is the controlled test of whether the algorithm can recover it. Constraints are the same as VK1, the steady-state equality against the generated b, and bounds scaled to include the test concentrations and fluxes, but no moiety conservation. It converged, with the help of several reflected kinetic leaf iterates (cf. Supplementary Section F): to less than the kinetic equality tolerance tolEXP:=5e-5. An interesting point to note is that, at least for this model, the constraints defined uniquely the net internal flux.

Experiment 3 (VK3) — recover a moiety-conserved steady state. Same generate-and-recover setup as VK2, with the added requirement that the recovered steady state also respect moiety conservation, so the feasible set gains the conserved-moiety linear equalities on top of VK2’s kinetics, steady-state, and bound blocks. Converged maximum kinetic equality violation to approximately the kinetic equality tolerance tolEXP:=5e-5, at major iteration 18. An interesting point to note is that, at least for this model, the constraints defined uniquely the net internal flux, and less so the unidirectional fluxes, but not concentrations.

## 11 Discussion

Variational kinetics is the first computationally tractable modelling method that enables satisfaction of elementary reaction kinetic rate law constraints at genome-scale without recourse to mathematical approximation. Given a biochemical network with m molecular species and n reactions, the problem of finding an m dimensional concentration vector within the non-convex set satisfying elementary reaction kinetics and equilibrium or non-equilibrium steady state constraints is expressed as the minimisation of a strictly concave function over the intersection of convex exponential cones and an affine subspace defined by linear equalities and inequalities. The steady state constraint is the affine subspace, the concentration and unidirectional flux bounds are the linear inequalities, and the relaxations of the elementary kinetic constraints are the exponential cones. These coexist in one convex conic feasible set, and it is over that set that the strictly concave function is minimised. That function is strictly positive on the feasible set and attains a zero local minimum if, and only if, the 2n elementary kinetic constraints are satisfied, a pair for each reversible reaction. Its minimisation by a particular sequence of conic optimisation problems is guaranteed to find a solution to a variational kinetic problem, provided that such a solution exists.

The difficulty that this addresses is intrinsic to the problem. A set is non-convex when there exists a line between two points in that set where an interval of that line lies outside the set. It has long been recognised that the set of thermodynamically feasible steady state fluxes is non-convex [3], and the set of concentrations that also satisfy elementary kinetic rate law constraints is likewise non-convex. Furthermore, the fundamental equation defining the set of non-equilibrium steady states of a biochemical network, Eq. (28), has an asymmetric gradient, that is a transposed Jacobian that is not symmetric, so a non-equilibrium steady state cannot, in general, be obtained by minimisation of scalar valued function. This fact alone eliminates a wide variety of established optimisation algorithms. Numerically, Eq. (28) is not a monotone function for a wide variety of genome-scale metabolic models, and previously we demonstrated a specific biochemical network that was provably not monotone, but rather a generalised monotone function termed a duplomonotone function [34]. Whether all biochemical networks give rise to a duplomonotone function is an open question, though numerical tests support such a conclusion. It is the difficulty of categorising this fundamental equation in terms specific enough to admit established algorithms at high dimension that motivates the development of tailored algorithms.

An optimisation problem is convex when a convex function is minimised, equivalently when a concave function is maximised, over a convex set. It is conic when a linear function is optimised over a convex conic set, that is an intersection of convex cones. In the optimisation literature conic optimisation is therefore described as a subclass of convex optimisation, in which the objective is linear and each constraint defines a convex set, and since the minimisation of any convex function can be expressed as the minimisation of a linear function subject to conic constraints [41], that subclass is a structured one rather than a restrictive one, admitting polynomialtime algorithms whenever the cone is tractable. Minimising a strictly concave function over a conic set satisfies the convex set requirement but violates the convex objective requirement, so the minimisation lies outside the convex class. The direction matters, because maximising that same strictly concave function over the same set is a convex problem. When a strictly concave function is minimised over a conic set it attains its local minima on the extreme points of the feasible set, and where those cones have curved and continuous boundaries there is, in general, a continuum of local minima. From a biological perspective the exponential cone has substantial value for the mathematical modelling of biochemical networks, leveraging fundamental and applied algorithmic developments in this area [42, 43, 44]. Conic formulations enable powerful, structure-exploiting solvers, but they require the non-linear parts of a modelling problem to be expressible in terms of particular convex cones, which we have established is the case for non-linear kinetic and thermodynamic constraints. It is likely that there are other, lesser known cones of substantial relevance to biology. We envisage that this potential will encourage greater appreciation of conic optimisation among the biological modelling community, and motivate mathematical progress on the characterisation of novel cones that admit efficient optimisation algorithms.

A key advantage of variational kinetics is that it rests on a sequence of conic optimisation problems, and so inherits the tractability, reliability and scalability of convex optimisation: efficient algorithms of polynomial-time complexity, solutions that are robust and reproducible, increasing solver support, and a well-developed duality theory. Every kinetic steady state corresponds to a local minimum of the strictly concave merit function, and every local minimum of that function is a global minimum, which avoids the non-global local minima, heuristics and starting point sensitivity that complicate both analysis and computation in alternative approaches. It is expected that, in general, there exist multiple kinetic steady states compatible with the given constraints, and it is a strength of variational kinetics that the sequence of conic optimisation problems is not intrinsically biased toward any particular one of them. That absence of bias is not the same as independence of the initial point. Each conic optimisation problem in the sequence starts from an initial feasible point, and which of the admissible kinetic steady states is returned does depend on that point, so further analysis is required to establish the nature of this dependence. We hypothesise that such analysis will be tractable, given the substantial literature relating perturbations in convex optimisation parameters to perturbations in their solutions, e.g., [45].

Although exponential cones represent relaxations of elementary kinetics, the convexity of the feasible set guarantees that an optimal solution satisfies elementary reaction kinetic constraints to within the numerical tolerances set by the optimisation solver. This presumes that a steady state kinetic solution exists [30], that the objective driving satisfaction of elementary kinetics is not dominated by a competing objective, and that the numerical values of the data, e.g., the stoichiometric coefficients [46], are sufficiently well scaled in comparison to the precision of the numerical implementation. The computational experiments reported herein exercise these properties in three regimes. The first isolates satisfaction of elementary reaction kinetics, with the competing penalties inactive and violation of mass balance admitted under a quadratic penalty. The second tests whether a kinetic steady state that is known to exist can be recovered from the boundary condition that it satisfies. The third repeats that test with moiety conservation imposed, so that the predicted steady state must conserve moieties as well as satisfy the same boundary condition.

Set against these merits are several limitations, some intrinsic and some that we envisage being overcome in future work. A disadvantage of any comprehensive approach to modelling biochemical reaction networks is that each type of constraint or data requires the definition of a new variable, whose biochemical interpretation depends on prior understanding of the mathematical form of the physicochemical and biochemical constraints concerned. As the number of different constraints and variables increases, any modelling formalism becomes more challenging to understand. This is compounded in conic optimisation, where auxiliary variables are required to express the problem in a form amenable to solution with established conic optimisation solvers. Some of these variables have accessible biochemical interpretations, e.g., an upper bound on the total internal net reaction rate, while others are unfamiliar and therefore harder to interpret biochemically. Our approach to managing this proliferation is a consistent nomenclature, with subscripts specific to particular instances of the same type of variable, and repeated use of as few cones as possible. Nevertheless, understanding this approach does require a basic familiarity with conic optimisation, which is less widespread in the biochemical modelling community than, say, linear optimisation.

A second limitation concerns the rate laws themselves. Elementary kinetic rate laws based on mass-action kinetics are valid only for true elementary steps under ideal, well-mixed, dilute conditions. Where a reaction involves complex mechanisms, non-ideal behaviour, heterogeneous phases, transport limitations or biological regulation, alternative mechanistic or phenomenological rate laws are required. Genome-scale models predominantly represent enzyme-catalysed reactions as overall rather than elementary reactions, which is partly an artefact of their development for prediction with early constraint-based modelling approaches, and partly a pragmatic response to pathways whose stoichiometry or chemical structural specification is unclear for want of experimental data, e.g., lipid metabolism. Lumped reactions create a particular difficulty here, because the standard transformed reaction Gibbs energy of a lumped reaction is the sum over the series of overall reactions lumped together. An artificially large negative standard transformed reaction Gibbs energy makes the thermodynamic constraint on the difference between logarithmic pseudoelementary kinetic parameters numerically awkward, since the relative values of those parameters then differ greatly in magnitude. In turn the unidirectional forward rate becomes artificially large and the reverse rate artificially small, or vice versa, either of which strains the finite precision arithmetic of a numerical optimisation solver. This motivates continued reconstruction effort, combining manual and algorithmic approaches, to split lumped reactions, and it warrants further analysis of whether variational kinetics can be extended to phenomenological kinetic rate laws [47, 48] applied systematically at genome-scale through a formalism admissible to tractable computation, e.g., convenience kinetics [49].

Herein variational kinetics is presented primarily as a means to represent elementary kinetic constraints for a biochemical reaction network in which all molecular species concentrations are assumed to be at steady state. That assumption can be relaxed by admitting deviation from steady state under a quadratic penalty, where the deviation may be interpreted as a discrete differential of molecular species concentrations with a time unit consistent with the reaction rates. We likewise admit quadratic penalisation of deviation from given molecular species concentrations, assuming that the mean concentrations are compatible with a steady state. One could then consider an iterative scheme in which molecular species concentrations are fixed at the sum of the concentrations and the concentration deviation from the previous iteration, so that the quadratic penalty enforces a form of continuity, or smoothing, of trajectories with respect to time. Application to biochemical network dynamics via damped gradient flow is thus one of a variety of natural theoretical extensions.

## 12 Conclusions

Variational kinetics is a novel, scalable biochemical network modelling method that enables satisfaction of elementary reaction kinetics in non-equilibrium steady states, optionally with the addition of moiety conservation and thermodynamic constraints on elementary kinetic parameters. These constraints couple variables representing unidirectional fluxes, molecular species concentrations and, optionally, (pseudo)elementary kinetic variables, unless they are specified as given parameters. All variables can be constrained by given lower and upper bounds. Optionally, quadratic penalisation of deviations from steady state constraints enables a kinetically feasible state to be obtained despite conflicting constraints. Linear optimisation of external net reaction rates enables representation of biologically motivated objectives, subject to the aforementioned constraints. The method is implemented by a sequence of conic optimisation problems that is globally convergent to an elementary reaction kinetic steady state, which is demonstrated to exist for any stoichiometrically consistent biochemical network. Variational kinetics is envisaged to provide a foundation for genome-scale biochemical network modelling, extending the focus of constraint-based modelling beyond metabolism and bringing kinetic modelling within reach at genome-scale.

## Supplementary Material

Supplement 1

## Data Availability

The genome-scale metabolic model used (code/ data/ raw/ iDopaNeuroC_VK_withL.mat), the computational narrative (src/ matlab/ JTB/ driver_optimizeVKmodel_VK1to3.mlx) that reproduces the numerical experiments in Section 10, as well as all of the source code implementing variational kinetics, including the sequential conic solver is available at https://github.com/Digital-Metabolic-Twin-Centre/varkin (SHA-1 b130be3). Reproduction of numerical computations requires a numerical computing environment MATLAB R2024b (Mathworks Inc.), the COBRA Toolbox (v3.8beta, specifically SHA-1 shorthand: 4713424eb) and a conic optimisation solver (MOSEK Version 11.2.0, feasibility and optimality tolerance 1*e* − 6), a commercial solver for which free academic licences are available. The absolute paths in the narrative have to be updated to reflect the location that, e.g, iDopaNeuroC_VK_withL.mat exists on each system.

## Footnotes

1 Strictly, it is not proper to take the logarithm of a unit that has physical dimensions. This difficulty can be avoided by considering c as a vector of mole fractions rather than concentrations (Eq. 19.93 in [26]).

2 Such problems are solved using a numerical optimisation solver that represents real valued numbers in finite precision, so typically a value of $x_{i}^{⋆}=10^{-6}$ or less should be considered zero (though this depends on the optimisation solver).

3 Warning: some articles in the literature define the same exponential cone but with a different convention for the order of the variables $x_{1},x_{2},x_{3}$. Throughout, we stick to the convention adopted by the MOSEK conic optimisation solver an its associated documentation, e.g., https://docs.mosek.com/modeling-cookbook/expo.html
