# Supplementary material for: Variational kinetics: elementary reaction kinetics via conic optimisation
Source: ArXiv. 2026 Aug 3:arXiv:2607.25217v2. Preprint. [Version 2] (PMC13484415)
Supplement: Supplementary file 1 [file NIHPP2607.25217v2-supplement-1.pdf]

A Local rate-law approximations

Figure 4 illustrates how local rate-law approximations lose accuracy away from the reference state.

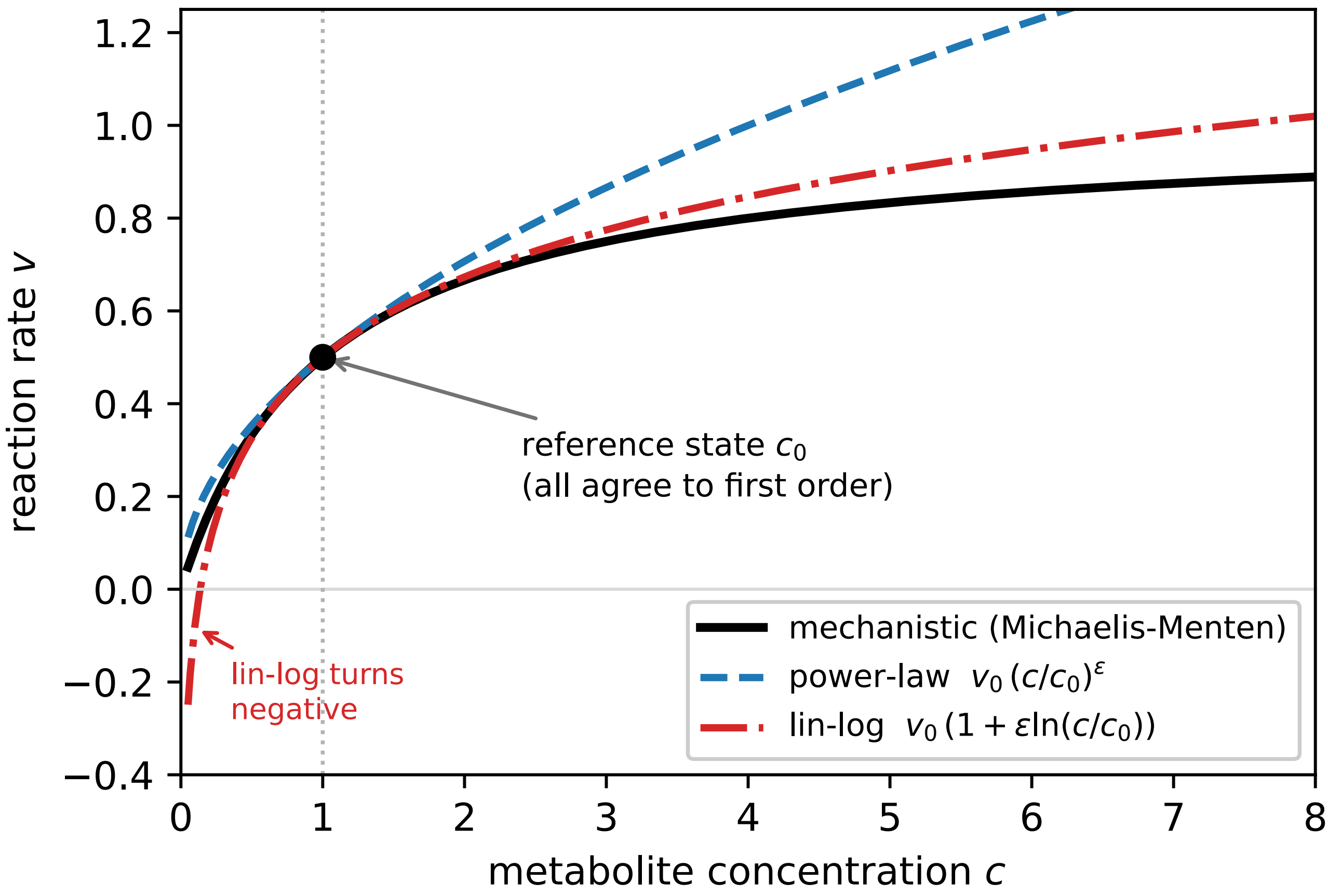

Figure 4: Local rate-law approximations lose accuracy away from the reference state. The mechanistic Michaelis-Menten rate $v = V_{\max} c/(K_M + c)$ (black) is approximated at a reference concentration c_0 by the power-law / S-system form $v_0(c/c_0)^\varepsilon$ (blue) and the linear-logarithmic (lin-log) form $v_0(1+\varepsilon \ln(c/c_0))$ (red), both built from the reference elasticity $\varepsilon = K_M/(K_M + c_0)$. All three agree to first order at c_0 but diverge away from it, the lin-log form even turning negative at low concentration.

B Elementary exponential and logarithmic identities

Let $x, y \in \mathbb{R}_{>0}$, then the following hold

$$
\begin{aligned}
\exp(\ln(x)) &= x \\
\exp(x+y) &= \exp(x)\cdot\exp(y) \\
\ln(xy) &= \ln(x)+\ln(y) \\
xy = \exp(\ln(xy)) &= \exp(\ln(x)+\ln(y)) = \exp(\ln(x))\cdot\exp(\ln(y)) \\
x^2 y = \exp(2\ln(x)+\ln(y)) &= \exp(2\ln(x))\cdot\exp(\ln(y)) \\
\prod x_i^{a_i} &= \exp(a^T\cdot\ln(x)) \\
\exp(0) &= 1 \\
\ln\left(\frac{x}{y}\right) &= \ln(x)-\ln(y) \\
\ln(x^y) &= y\ln(x) \\
-\ln(x) &= \ln\left(\frac{1}{x}\right)
\end{aligned}
$$

C Cyclic stoichiometric matrix

Theorem 5. *Let*

$$\bar{N} := \begin{bmatrix} N & -I_m \\ 0 & L \end{bmatrix} \in \mathbb{R}^{(2m-r)\times(n+m)}, \tag{155}$$

where

$$N \in \mathbb{R}^{m\times n}, \qquad L \in \mathbb{R}^{(m-r)\times m},$$

satisfy

$$LN = 0, \qquad rank(N) = r, \qquad rank(L) = m - r.$$

Then:
(i) The left nullspace of $\bar{N}$ *is*

$$\mathcal{N}_\ell(\bar{N}) = \left\{ \begin{bmatrix} L^T \\ I_{m-r} \end{bmatrix} y \ : \ y \in \mathbb{R}^{m-r} \right\}, \tag{156}$$

so a basis is given by the rows of $[L \quad I_{m-r}]$*, and*

$$\dim \mathcal{N}_\ell(\bar{N}) = m - r.$$

(ii) The right nullspace of $\bar{N}$ *is*

$$\mathcal{N}_r(\bar{N}) = \left\{ \begin{bmatrix} I_n \\ N \end{bmatrix} v \ : \ v \in \mathbb{R}^{n} \right\}, \tag{157}$$

so a basis is given by the columns of $\begin{bmatrix} I_n \\ N \end{bmatrix}$*, and*

$$\dim \mathcal{N}_r(\bar{N}) = n.$$

(iii) The rank of $\bar{N}$ *is*

$$rank(\bar{N}) = m.$$

Proof. Left nullspace. Let $y \in \mathbb{R}^{2m-r}$ be a left-null vector of $\bar{N}$, written in block form as

$$y = \begin{bmatrix} a \\ b \end{bmatrix}, \qquad a \in \mathbb{R}^m, \ b \in \mathbb{R}^{m-r}.$$

Then $y^T \cdot \bar{N} = 0$ is equivalent to

$$\begin{cases} a^T \cdot N = 0, \\ -a^T + b^T \cdot L = 0. \end{cases} \tag{158}$$

From $a^T \cdot N = 0$ and the assumption that the rows of L span the left nullspace of N, there exists $t \in \mathbb{R}^{m-r}$ such that $a^T = t^T \cdot L$. Substituting into the second equation of 158 gives

$$-t^T \cdot L + b^T \cdot L = 0 \quad \Longleftrightarrow \quad (b^T - t^T)L = 0.$$

Since $\text{rank}(L) = m - r$, L has full row rank, hence $(b^T - t^T)L = 0$ implies $b^T = t^T$. Therefore

$$y^T = [t^T \cdot L \quad t^T] = t^T \cdot [L \quad I_{m-r}].$$

Thus every left-null vector of $\bar{N}$ is a linear combination of the rows of $[L \quad I_{m-r}]$.

Conversely, a direct computation yields

$$[L \quad I_{m-r}] \begin{bmatrix} N & -I_m \\ 0 & L \end{bmatrix} = [LN \quad -L + L] = [0 \quad 0],$$

so every row of $[L \quad I_{m-r}]$ lies in $\mathcal{N}_\ell(\bar{N})$. Because these rows are linearly independent, they form a basis and $\dim \mathcal{N}_\ell(\bar{N}) = m - r$. Equivalently,

$$\mathcal{N}_\ell(\bar{N}) = \left\{ \begin{bmatrix} L^T \\ I_{m-r} \end{bmatrix} y : \ y \in \mathbb{R}^{m-r} \right\}.$$

Right nullspace. Let $x \in \mathbb{R}^{n+m}$ be written as

$$x = \begin{bmatrix} v \\ w \end{bmatrix}, \qquad v \in \mathbb{R}^n, \ w \in \mathbb{R}^m.$$

Then $\bar{N}x = 0$ is equivalent to the block equations

$$Nv - w = 0, \qquad Lw = 0. \tag{2}$$

The first equation in (2) implies $w = Nv$. Substituting into the second gives

$$Lw = LNv = 0,$$

which holds for every $v \in \mathbb{R}^n$ because $LN = 0$. Hence

$$\mathcal{N}_r(\bar{N}) = \left\{ \begin{bmatrix} v \\ Nv \end{bmatrix} : \ v \in \mathbb{R}^n \right\},$$

and the columns of $\begin{bmatrix} I_n \\ N \end{bmatrix}$ form a basis, so $\dim \mathcal{N}_r(\bar{N}) = n$.

Rank. By rank–nullity applied to $\bar{N} \in \mathbb{R}^{(2m-r)\times(n+m)}$,

$$\mathrm{rank}(\bar{N}) + \dim \mathcal{N}_\ell(\bar{N}) = 2m - r,$$

so using $\dim \mathcal{N}_\ell(\bar{N}) = m - r$ yields

$$\mathrm{rank}(\bar{N}) = (2m - r) - (m - r) = m.$$

□

Given 155, the forward and reverse stoichiometric matrices are

$$\bar{F} \ := \begin{bmatrix} F & I_m \\ 0 & 0 \end{bmatrix} \qquad \bar{R} \ := \begin{bmatrix} R & 0 \\ 0 & L \end{bmatrix} \in \mathbb{R}^{(2m-r)\times(n+m)},$$

and their horizontal concatenation is

$$\begin{bmatrix} \bar{F}, & \bar{R} \end{bmatrix} = \begin{bmatrix} F & I_m & R & 0 \\ 0 & 0 & 0 & L \end{bmatrix}$$

with $\mathrm{rank}\left(\begin{bmatrix} \bar{F}, & \bar{R} \end{bmatrix}\right) = 2m - r$, since $\mathrm{rank}\,(L) = m - r$. Note that $\mathrm{rank}\left(\begin{bmatrix} F, & I_m, & R \end{bmatrix}\right) = m$ irrespective of $\mathrm{rank}\left(\begin{bmatrix} F, & R \end{bmatrix}\right)$.

D Optimality conditions

The Lagrangian corresponding to the combined formulation of variational kinetics expressed as Problem (136)-(154) in Section 9 is

$$\begin{aligned}
&\mathcal{L}(v_f, v_r, lnk_f, lnk_r, lnc, c, u^\circ, r, w, t_r, \ldots \\
&y_N, y_{u^\circ}, y_L, s_{v_f}, s_{f_1}, s_F, s_{v_r}, s_{r_1}, s_R, s_c, s_{c_1}, s_{lnc}, s_{ce}, s_{t_r}, s_{r1}, s_{H_r}) := \\
&c_{v_f}^T \cdot v_f + c_{v_r}^T \cdot v_r + c_{lnk_f}^T \cdot lnk_f + c_{lnk_r}^T \cdot lnk_r + c_w^T \cdot w + c_{lnc}^T \cdot lnc + c_c^T \cdot c \ldots \\
&+ c_{t_r}^T \cdot t_r \ldots \\
&+ y_N^T \cdot (N \cdot (v_f - v_r) + r + B \cdot w) \ldots \\
&+ y_{u^\circ}^T \cdot (lnk_f - lnk_r + N^T \cdot u^\circ) \ldots \\
&+ y_L^T \cdot (L \cdot c - L \cdot c(0)) \ldots \\
&+ \begin{pmatrix} s_{v_f} \\ s_{f_1} \\ s_F \end{pmatrix}^T \cdot \begin{pmatrix} v_f \\ 1 \\ F^T \cdot lnc + lnk_f \end{pmatrix} + \begin{pmatrix} s_{v_r} \\ s_{r1} \\ s_R \end{pmatrix}^T \cdot \begin{pmatrix} v_r \\ 1 \\ R^T \cdot lnc + lnk_r \end{pmatrix} + \begin{pmatrix} s_c \\ s_{c_1} \\ s_{lnc} \end{pmatrix}^T \cdot \begin{pmatrix} c \\ 1 \\ lnc \end{pmatrix} \ldots \\
&+ \begin{pmatrix} s_{t_r} \\ s_{r1} \\ s_{H_r} \end{pmatrix}^T \cdot \begin{pmatrix} t_r \\ 1 \\ H_r \cdot r \end{pmatrix} \ldots \\
&+ z_{v_f}^T \cdot (u_{v_f} - v_f) + z_{v_r}^T \cdot (u_{v_r} - v_r) \ldots \\
&+ z_{lv}^T \cdot ((v_f - v_r) - l_v) + z_{uv}^T \cdot (u_v - (v_f - v_r)) + z_{lw}^T \cdot (w - l_w) + z_{uw}^T \cdot (u_w - w) \ldots \\
&+ z_{llnkf}^T \cdot (lnkf - l_{lnkf}) + z_{ulnkf}^T \cdot (u_{lnkf} - lnkf) + z_{llnkr}^T \cdot (lnkr - l_{lnkr}) + z_{ulnkr}^T \cdot (u_{lnkr} - lnkr) \ldots \\
&+ z_{llnc}^T \cdot (lnc - l_{lnc}) + z_{ulnc}^T \cdot (u_{lnc} - lnc) + z_{lu^\circ}^T \cdot (u^\circ - l_{u^\circ}) + z_{uu^\circ}^T \cdot (u_{u^\circ} - u^\circ),
\end{aligned}$$

where we require that the following dual variables are non-negative $z_{v_f}, z_{v_r}, z_{lv}, z_{uv}, z_{llnkf}, z_{ulnkf}, z_{llnkr}, z_{ulnkr} \in \mathbb{R}^n_{\geq 0}$, $z_{lw}, z_{uw} \in \mathbb{R}^k_{\geq 0}$, $z_{llnc}, z_{ulnc}, z^T_{lu^\circ}, z_{uu^\circ} \in \mathbb{R}^m_{\geq 0}$ and that the primal and dual variables are feasible with respect to the primal and dual cones, that is

$$\begin{pmatrix} v_f \\ 1 \\ F^T \cdot lnc + lnk_f \end{pmatrix} \in \mathcal{K}^n_{exp}, \qquad \begin{pmatrix} s_{vf} \\ s_{f1} \\ s_F \end{pmatrix} \in \mathcal{K}^{\star n}_{exp}, \tag{159}$$

$$\begin{pmatrix} v_r \\ 1 \\ R^T \cdot lnc + lnk_r \end{pmatrix} \in \mathcal{K}^n_{exp}, \qquad \begin{pmatrix} s_{vr} \\ s_{r1} \\ s_R \end{pmatrix} \in \mathcal{K}^{\star n}_{exp}, \tag{160}$$

$$\begin{pmatrix} c \\ 1 \\ lnc \end{pmatrix} \in \mathcal{K}^m_{exp}, \qquad \begin{pmatrix} s_c \\ s_{c1} \\ s_{lnc} \end{pmatrix} \in \mathcal{K}^{\star m}_{exp}, \tag{161}$$

$$\begin{pmatrix} t_r \\ 1 \\ H_r \cdot r \end{pmatrix} \in \mathcal{Q}^{2+m}, \qquad \begin{pmatrix} s_{t_r} \\ s_{r1} \\ s_{H_r} \end{pmatrix} \in \mathcal{Q}^{\star 2+m}. \tag{162}$$

Derivation of the optimality conditions to a general conic optimisation problem are described elsewhere ($5.9.2 [32]). The optimality conditions to Problem (136)-(154) may be obtained by setting the partial derivatives of the Lagrangian with respect to the variables $v_f, v_r, lnk_f, lnk_r, lnc, c, u^\circ, r, w, t_r, y_N, y_{u^\circ}$ and y_L to zero, that is

$$\frac{\partial \mathcal{L}}{\partial v_f} = c_{v_f} - N^T \cdot y^\star_N - s^\star_{vf} - s^\star_{ef1} + s^\star_{ef} - s^\star_{vfe} - z^\star_{v_f} + z^\star_v = 0 \tag{163}$$

$$\frac{\partial \mathcal{L}}{\partial v_r} = c_{v_r} + N^T \cdot y^\star_N - s^\star_{vr} - s^\star_{er1} + s^\star_{er} - s^\star_{vre} - z^\star_{v_r} - z^\star_v = 0 \tag{164}$$

$$\frac{\partial \mathcal{L}}{\partial lnk_f} = c_{lnk_f} - y^\star_{u^\circ} - s^\star_F - z^\star_{lnkf} = 0 \tag{165}$$

$$\frac{\partial \mathcal{L}}{\partial lnk_r} = c_{lnk_r} + y^\star_{u^\circ} - s^\star_R - z^\star_{lnkr} = 0 \tag{166}$$

$$\frac{\partial \mathcal{L}}{\partial lnc} = c_{lnc} - F \cdot s^\star_F - R \cdot s^\star_R - s^\star_{lnc} - z^\star_{lnc} = 0 \tag{167}$$

$$\frac{\partial \mathcal{L}}{\partial c} = c_c + s^\star_{c_e} - s^\star_c - z^\star_c + L^T \cdot y^\star_L = 0 \tag{168}$$

$$\frac{\partial \mathcal{L}}{\partial u^\circ} = c_{u^\circ} - z^\star_{u^\circ} + N \cdot y^\star_{u^\circ} = 0 \tag{169}$$

$$\frac{\partial \mathcal{L}}{\partial r} = c_r - H_r \cdot s^\star_{H_r} - z^\star_r + y^\star_N = 0 \tag{170}$$

$$\frac{\partial \mathcal{L}}{\partial w} = c_w - B^T \cdot y^\star_N - z^\star_w = 0 \tag{171}$$

$$\frac{\partial \mathcal{L}}{\partial t_r} = c_{t_r} - s^\star_{t_r} - z^\star_{t_r} = 0 \tag{172}$$

$$\frac{\partial \mathcal{L}}{\partial y_N} = N \cdot (v^\star_f - v^\star_r) + r^\star + B \cdot w^\star = 0$$

$$\frac{\partial \mathcal{L}}{\partial y_{u^\circ}} = lnk^\star_f - lnk^\star_r + N^T \cdot u^{\circ\star} = 0 \tag{173}$$

$$\frac{\partial \mathcal{L}}{\partial y_L} = L \cdot c^\star - L \cdot c(0) = 0 \tag{174}$$

and expressing the complementarity constraints between primal and dual terms, that is

$$\begin{pmatrix} s^{\star}_{v_f} \\ s^{\star}_{f1} \\ s^{\star}_{F} \end{pmatrix}^T \cdot \begin{pmatrix} v^{\star}_f \\ 1 \\ F^T \cdot lnc^{\star} + lnk^{\star}_f \end{pmatrix} = 0^{nx1} \tag{175}$$

$$\begin{pmatrix} s^{\star}_{v_r} \\ s^{\star}_{r1} \\ s^{\star}_{R} \end{pmatrix}^T \cdot \begin{pmatrix} v^{\star}_r \\ 1 \\ R^T \cdot lnc^{\star} + lnk^{\star}_r \end{pmatrix} = 0^{nx1}$$

$$\begin{pmatrix} s^{\star}_{c} \\ s^{\star}_{c1} \\ s^{\star}_{lnc} \end{pmatrix}^T \cdot \begin{pmatrix} c^{\star} \\ 1 \\ lnc^{\star} \end{pmatrix} = 0^{mx1} \tag{176}$$

$$\begin{pmatrix} s^{\star}_{t_r} \\ s^{\star}_{r1} \\ s^{\star}_{H_r} \end{pmatrix}^T \cdot \begin{pmatrix} t^{\star}_r \\ 1 \\ H_r \cdot r^{\star} \end{pmatrix} = 0^{1\times 1} \tag{177}$$

where 0^{nx1} denotes a set of n complementarity constraints, one for each reaction, and similarly for other zero vectors as indicated above. The final parts of the optimality conditions to Problem (136)-(154) are to specify that the primal and dual terms are constrained to reside within primal and dual conic cones, respectively, that is Eqs. (159)-(162), with the addition of * to each variable to denote optimality.

E Chemical potential

We assume constant temperature, $\mathcal{T} = 310.15$ K, constant pressure, $\mathcal{P} = 1$ atm, and let $x \in \mathbb{R}^m$ be a vector of mole fractions of molecular species in a solution. Assuming an ideal solution, the chemical potential is

$$u := u^{\circ}(\mathcal{T}, \mathcal{P}, x^{\circ}) + \mathcal{R}\mathcal{T} \ln(x)$$

where $u^{\circ}(\mathcal{T}, \mathcal{P}, x^{\circ})$ is the chemical potential at a standard mole fraction $x^{\circ} \in \mathbb{R}^m$ (3.7.1.2.2 in [50]) and $\mathcal{R}$ is the gas constant. The molar concentration of pure water is $c^{\circ}_w = 55.28$ mol/L, while in typical biochemical solutions the estimated total concentration of solutes is ~0.3 mol/L [51], which is $\sim$ 180 times less than the molar concentration of water solvent so a distinction between solvent and solute is appropriate (5.3 in [52]. We assume Raoult's law for the solvent, where the standard mole fraction approaches unity, $x^{\circ}_w \to 1$, and corresponds to a molar concentration of pure water $c^{\circ}_w = 55.28$ mol/L, consistent with pure water at the aforementioned temperature and pressure. We assume Henry's law for each solute, where the standard mole fraction approaches zero, $x^{\circ} \to 0$, and we assume the standard concentration of each solute is $c^{\circ} = 1$ mol/L.

Following an established approach to biochemical thermodynamics [53], refined for multi-compartmental systems [54], we assume constant compartment-specific pH [54], constant compartment-specific electrical potentials [54], and define standard transformed chemical potential as

$$u^{\circ\prime} := u^{\circ}(\mathcal{T}, \mathcal{P}, x^{\circ}) + \mathcal{R}\mathcal{T} \ln(a),$$

where $a \in \mathbb{R}^m$ is the *activity coefficient* of each molecular species, estimated using the extended Debye-Hückel equation (§3.6 in [53]). This absorbs an approximation to non-ideal behaviour into the standard transformed term, enabling definition of transformed chemical potential as

$$\begin{aligned} u &:= u^{\circ\prime} + \mathcal{R}\mathcal{T} \ln(x) \\ &= u^{\circ\prime} + \mathcal{R}\mathcal{T} \ln\left(\frac{c}{\mathbf{1}^T \cdot c}\right) \end{aligned} \tag{178}$$

where we assume $x = {}^{c}/_{\mathbf{1}^T \cdot c}$, where $c \in \mathbb{R}^m$ is a vector of molar concentrations. Transformed (standard) chemical potential is usually distinguished from (standard) chemical potential with $'$ but henceforth we assume (standard) chemical potential is transformed and omit the prime for clarity. For an ideal-dilute solution, assuming the approximation $\mathbf{1}^T \cdot c \approx c^{\circ}_w$, standard chemical potential in terms of concentration may be related (Eq 3.7-23 in [50]) to standard chemical potential in terms of mole fraction using

$$u^{\circ}(\mathcal{T}, \mathcal{P}, c^{\circ}) := u^{\circ}(\mathcal{T}, \mathcal{P}, x^{\circ}) - \mathcal{R}\mathcal{T} \ln(c^{\circ}_w). \tag{179}$$

F Adaptive Sequential Conic Linear Approximation Algorithm

F.1 Purpose and problem class

This section describes the adaptive sequential conic linear approximation algorithm used to solve the combined formulation of variational elementary kinetics of Section 9. That problem is itself a conic optimisation problem,

so a single conic solve returns an optimal solution, but not in general one at which the exponential cone constraints representing elementary kinetics are active. It is activity of those constraints that makes an optimal solution satisfy elementary reaction kinetics, and Theorem 4 establishes that, for the constraints of variational elementary kinetics, every stationary point of the iteration below has that property. The algorithm therefore treats the linear objective coefficients on the kinetic exponential-cone variables as parameters rather than as given data, and updates them across a sequence of conic optimisation problems until each of those constraints is active.

Every problem in that sequence is an inner problem, solved by a conic optimiser, whose endpoint supplies only a candidate search direction. Progress is measured instead by a nonlinear outer merit, assembled from the exponential-cone boundary residuals and evaluated after each candidate step, so the inner objective that generates a direction is distinct from the outer merit that decides whether the resulting step is accepted. Theorem 3 of Section 6 establishes, for the abstract sequence, convergence to a stationary point of the outer merit subject to the constraints. What follows is a concrete realisation of that scheme, together with the initialisation, step safeguards and adaptive strategy portfolio that it requires in order to converge on a genome-scale model.

The next subsection states the algorithm in full as pseudocode. The subsections after it define each of its ingredients in turn: the base conic model, the exponential-cone boundary residuals and the outer merit derived from them, the working scaling and fixed columns, the construction of an initial point, the portfolio of inner conic models, the step safeguards and line search, the portfolio controller, and the finalisation and audit of the returned primal-dual tuple. The description is independent of programming language and follows the notation of Section 2.

F.2 Algorithmic summary

The following summary gives the logical flow of the algorithm, in the order of the subsections below.

Definitions.
  Working feasible set $\mathcal{X} := \{x \in \mathbb{R}^n : b_l \leq A \cdot x \leq b_u,\ l \leq x \leq u,\ F \cdot x + d \in \mathcal{K}\}$.
  Active reduced exponential-cone images $t_1(x) := F_1 \cdot x + d_1$ and $t_3(x) := F_3 \cdot x + d_3$.
  Boundary residuals $h(x) := t_1(x) - \exp(t_3(x))$ and $g(x) := \ln(t_1(x)) - t_3(x)$.
  Outer merit $\phi(x) := \mathbf{1}^T \cdot h(x) + \mathbf{1}^T \cdot g(x)$.
  Convergence residual $\theta(x) := \max\{\|h(x)\|_\infty, \|g(x)\|_\infty\}$.
Working scaling and fixed columns.
1 if explicit row scaling is active then $A \leftarrow S \cdot A$, $b_l \leftarrow S \cdot b_l$ and $b_u \leftarrow S \cdot b_u$, leaving F, d, l, u and the caller objective a unscaled.
2 $\mathcal{F} \leftarrow$ the structurally fixed columns; when projection is enabled, every candidate start, inner endpoint and accepted trial point is projected onto $\{x : x_\mathcal{F} = l_\mathcal{F} = u_\mathcal{F}\}$.
Initialisation.
3 $a_0 \leftarrow a$ with the active merit-column entries set to zero.
4 for each start mode, in the order centred feasible, elastic feasible, feasible, relaxed feasible, all-ones, do
5   for the centred feasible mode, solve the elastic Phase I problem, then the centring problem driving $t_1 \to \mathbf{1}$ and $t_3 \to 0$, then, if the raw explicit residual exceeds the repair threshold, the one-norm-movement-penalised repair problem.
6   $x^0 \leftarrow$ the candidate point, projected onto the fixed-column subspace.
7   if $r_{\text{exp}}(x^0) \leq \tau_{\text{start}}$, or the soft repaired-centred allowance is met, then accept x^0 and leave the loop.
8 if no start mode is accepted then stop: there is no usable initial point.
Outer loop.
9 $k \leftarrow 0$; select the first strategy of the portfolio.
10 while $k < k_{\max}$ do
11   evaluate $\phi(x^k)$, h, g, $\theta(x^k)$, $\nabla\phi(x^k)$ and the curvature diagnostics.
12   if $\theta(x^k) \leq \tau_{\text{stop}}$ then stop: the outer loop has converged.
13   repeat
14   build the inner conic model of the current strategy over $\mathcal{X}$:
      cost approximation, minimising $(c_{\text{in}}^k)^T \cdot x$ with c_{in}^k the active merit gradient;
      reachable-boundary cost approximation, adding the boundary attraction $\eta_{\text{bd}}^k (a_{\text{bd}}^k)^T \cdot x$ and the target-centred rotated quadratic penalty;

block-curvature regularised cost approximation;
residual-balanced quadratically regularised cost approximation;
quadratically regularised cost approximation;
local-box quadratically regularised cost approximation, which adds temporary affine-image bounds on t_1 and t_3.

15 solve the inner conic model and set $d^k \leftarrow x^k_{\text{in}} - x^k$; for a safeguarded gradient strategy set $d^k \leftarrow -\nabla\phi(x^k) \oslash \max\{1, \|\nabla\phi(x^k)\|_\infty\}$ instead.
16 if the inner solve failed, or d^k is not finite, then advance the strategy and return to step 14.
17 if $\nabla\phi(x^k)^T \cdot d^k \geq -\tau_{\text{desc}}$, and the curvature-rescue model does not admit d^k, then advance the strategy and return to step 14.
18 $\lambda \leftarrow$ the least of the step caps: full step, explicit bound, log domain, exponential boundary, coefficient change and numerical movement.
19 while $\lambda > \lambda_{\min}$ do
20 $x(\lambda) \leftarrow x^k + \lambda d^k$, projected onto the fixed-column subspace.
21 accept $x(\lambda)$ if $r_{\exp}(x(\lambda)) \leq \tau_{\exp}$, if $t_1(x(\lambda)) > \eta\mathbf{1}$, if $\phi(x(\lambda)) \leq \phi(x^k) + \sigma\lambda\nabla\phi(x^k)^T \cdot d^k$, and, once $\theta(x^k)$ has entered the moderate-residual regime, if $\theta(x(\lambda)) \leq \theta(x^k) - \delta_\theta + \tau_\theta$.
22 if $x(\lambda)$ is accepted then leave the line search, else $\lambda \leftarrow \beta\lambda$.
23 if no trial point was accepted, or the accepted step is a microscopic boundary-limited step, then advance the strategy and return to step 14.
24 until a step is accepted, or every strategy has been attempted
25 if every strategy has been attempted without an accepted step then stop: the portfolio is exhausted.
26 $x^{k+1} \leftarrow x^k + \lambda_k d^k$ and $k \leftarrow k+1$.
27 update the portfolio controller: after repeated calm accepted steps, probe back toward cost approximation, unless the high-curvature, repeated no-descent or small-residual suppressors apply; advance to a safer strategy on stagnation, on curvature dominance, or on boundary saturation.

Finalisation.

28 $x_{\text{acc}} \leftarrow$ the last accepted outer iterate.
29 if polish is permitted then solve the unrestricted conic polish problem with the caller objective a over $\mathcal{X}$, and accept its endpoint only if it passes the final explicit-feasibility, outer-residual and merit gates.
30 if no polished point is accepted then solve the fixed-primal recovery problem for dual variables at x_{acc}, without moving the primal point.
31 if that also fails then return x_{acc} with zero dual placeholders and a failure status.
32 audit the returned tuple: θ_{fin}, the explicit residual in both the working and the original row units, and the Karush-Kuhn-Tucker residual of the returned pair.
33 report convergence from $\theta_{\text{fin}} \leq \tau_{\text{stop}}$.

F.3 Base conic model

Let the primal variable be a column vector $x \in \mathbb{R}^n$. The inner conic models are all built over a common feasible set, possibly augmented by temporary epigraph variables or temporary local rows. The unaugmented working feasible set is

$$\mathcal{X} := \{x \in \mathbb{R}^n : b_l \leq A \cdot x \leq b_u,\ l \leq x \leq u,\ F \cdot x + d \in \mathcal{K}\},$$

where

$$A \in \mathbb{R}^{p\times n}, \quad b_l, b_u \in (\mathbb{R} \cup \{-\infty, +\infty\})^p, \quad l, u \in (\mathbb{R} \cup \{-\infty, +\infty\})^n,$$

$$F \in \mathbb{R}^{q\times n}, \quad d \in \mathbb{R}^q, \quad \mathcal{K} = \mathcal{K}_1 \times \cdots \times \mathcal{K}_r.$$

All vector inequalities are understood componentwise. The finite lower and upper bounds in the first two displays define the explicit part of primal feasibility. The conic inclusion defines the affine conic part of primal feasibility. The caller-supplied linear objective is represented by a vector $a \in \mathbb{R}^n$, but this objective is not the outer merit used to control the iterative boundary-matching process. For the combined formulation of variational elementary kinetics of Section 9, the vector a collects the linear objective coefficients on the exponential-cone columns, which the algorithm treats as parameters and overwrites at every major iteration, together with the

two fixed coefficients c_{t_r} and c_w of Eqs. (138) and (139), which lie on columns that no exponential cone touches. The inner conic objective of every strategy below is assembled from the outer merit gradient on the exponential-cone columns alone, and is zero on every other column, so the two fixed terms $c_{t_r}^T \cdot t_r$ and $c_w^T \cdot w$ do not themselves steer the outer iteration. They act at two points only: the initial point is constructed from a with the exponential-cone entries set to zero, and the final polish solve minimises a over the working conic model, its endpoint being accepted only if it passes the final feasibility and residual gates.

For diagnostics and stopping tests, the explicit primal residual is defined as

$$r_{\exp}(x) := \max\left\{\|[b_l - A \cdot x]_+\|_\infty, \|[A \cdot x - b_u]_+\|_\infty, \|[l - x]_+\|_\infty, \|[x - u]_+\|_\infty\right\}.$$

Here $[\cdot]_+$ denotes the componentwise positive part. This residual is used as the raw explicit feasibility gate for accepted initial points and accepted outer trial points.

F.4 Exponential-cone boundary residuals

The algorithm focuses on a subset of primal exponential-cone blocks. For each active block, the second cone coordinate is fixed at one after reduction. Thus each active block is represented by

$$(x_1, x_2, x_3) = (t_1(x), 1, t_3(x)).$$

The MOSEK primal exponential-cone convention is

$$(x_1, x_2, x_3) \in \mathcal{K}_{exp} \iff x_1 \geq x_2 \exp(x_3/x_2), \quad x_1 > 0, \quad x_2 > 0.$$

The corresponding reduced feasibility condition is therefore

$$t_1(x) \geq \exp(t_3(x)), \quad t_1(x) > 0.$$

The active affine images are written as

$$t_1(x) := F_1 \cdot x + d_1 \in \mathbb{R}^m, \qquad t_3(x) := F_3 \cdot x + d_3 \in \mathbb{R}^m,$$

where $F_1, F_3 \in \mathbb{R}^{m \times n}$ select the first and third coordinates of the active reduced exponential-cone blocks after embedding them in the full primal space, with constant offsets $d_1, d_3 \in \mathbb{R}^m$. The two residual vectors are

$$h(x) := t_1(x) - \exp(t_3(x)),$$

$$g(x) := \ln(t_1(x)) - t_3(x).$$

The vector $h \in \mathbb{R}^m$ is an additive boundary gap. The vector $g \in \mathbb{R}^m$ is a logarithmic boundary gap. Both are zero on the reduced exponential-cone boundary $t_1 = \exp(t_3)$, and both are nonnegative in the strictly feasible reduced exponential-cone region. Their simultaneous reduction gives a scale-aware measure of approach to the target boundary.

The dual exponential cone is interpreted with the corresponding MOSEK convention

$$(s_1, s_2, s_3) \in \mathcal{K}_{exp}^* \iff s_1 \geq -s_3 \exp\left((s_2/s_3) - 1\right),$$

where $s \in \mathbb{R}^q$ denotes the affine-conic dual vector. This convention is relevant for final primal-dual auditing, but the outer iteration itself is driven primarily by primal residuals h and g.

F.5 Outer merit and derivatives

The default outer merit is the sum of the additive and logarithmic boundary gaps:

$$\phi(x) := \mathbf{1}^T \cdot h(x) + \mathbf{1}^T \cdot g(x).$$

The associated convergence residual is not the scalar merit but the maximum componentwise residual

$$\theta(x) := \max\left\{\|h(x)\|_\infty, \|g(x)\|_\infty\right\}.$$

This distinction is important. The scalar merit aggregates all active blocks and is useful for line search, whereas $\theta(x)$ is the reported nonlinear stopping residual. Late in the iteration, a trial point may reduce the sum merit while worsening the largest component. The algorithm may therefore impose an additional maximum-residual acceptance gate after $\theta(x)$ enters a moderate residual regime.

For the default merit, the gradient is

$$\nabla\phi(x) = F_1^T \cdot \left(\mathbf{1} + t_1(x)^{-1}\right) - F_3^T \cdot \left(\mathbf{1} + \exp(t_3(x))\right),$$

where $t_1(x)^{-1}$ denotes the componentwise reciprocal. The gradient of h is written, using the convention that the first dimension equals the number of variables and the second dimension equals the range dimension, as

$$\nabla h(x) = F_1^T - F_3^T \cdot \operatorname{diag}(\exp(t_3(x))) \in \mathbb{R}^{n\times m}.$$

F.6 Working scaling and fixed columns

Before the initial point is constructed, the explicit linear rows may be scaled by a positive diagonal matrix. If row scaling is active, the working explicit constraints become

$$A_{\text{sc}} := S \cdot A, \qquad b_{l,\text{sc}} := S \cdot b_l, \qquad b_{u,\text{sc}} := S \cdot b_u,$$

where $S \in \mathbb{R}^{p\times p}$ is a positive diagonal matrix. The affine conic image $F \cdot x + d$, the variable bounds l and u, and the original conic objective vector a are not scaled. Consequently, the exponential-cone boundary $t_1(x) = \exp(t_3(x))$ is unchanged. The entire initialisation, all inner models, all line searches, and finalisation use a single working representation, either scaled or unscaled. At return, explicit row duals are mapped back to the caller's original row units. Some variables may be structurally fixed. Let the fixed-column set be denoted by $\mathcal{F} \subseteq \{1, \dots, n\}$. The fixed-column affine subspace is

$$\{x \in \mathbb{R}^n : x_{\mathcal{F}} = l_{\mathcal{F}} = u_{\mathcal{F}}\}.$$

Candidate starts, inner endpoints, and accepted trial points are optionally projected back to this affine subspace. This prevents a small fixed-bound residual in the initial point from being inherited throughout the outer iteration.

F.7 Initialisation

The initialisation stage is not intended to minimise the original conic objective. Its purpose is to construct a numerically usable outer starting point x^0 in the working feasible set, compatible with fixed columns and well scaled for evaluating the nonlinear merit. To reduce the risk of choosing an extreme starting point, the active merit-column entries of the original conic objective are set to zero before the start problem is solved. The remaining inactive part may act only as a weak tie-breaker in the direct feasible start; the Phase I, centring and repair problems replace the objective entirely. The preferred initialisation is a centred feasible construction. First, an elastic Phase I conic problem is solved. This introduces nonnegative elastic variables that relax finite explicit row and variable bounds, while leaving the original affine conic geometry unchanged. In abstract form, the Phase I objective is

$$\min\ \mathbf{1}^T \cdot r_l^A + \mathbf{1}^T \cdot r_u^A + \mathbf{1}^T \cdot r_l^x + \mathbf{1}^T \cdot r_u^x,$$

subject to relaxed lower and upper explicit constraints and the original conic inclusion. The resulting point provides a feasible or nearly feasible reference for the second stage.

Second, a centring conic problem is solved over the original feasible set. Its role is to place the reduced exponential-cone images near the well-scaled boundary point $(1, 1, 0)$. A representative centring problem is

$$\min_{x,r_1,r_3}\ \mathbf{1}^T \cdot r_1 + \mathbf{1}^T \cdot r_3$$

$$\text{subject to } x \in \mathcal{X}, \qquad -r_1 \le t_1(x) - \mathbf{1} \le r_1, \qquad -r_3 \le t_3(x) \le r_3,$$

$$r_1 \ge 0, \qquad r_3 \ge 0.$$

Thus the start is encouraged to satisfy t_1 approximately equal to one and t_3 approximately equal to zero. These values correspond to the reduced exponential-cone boundary point $(x_1, x_2, x_3) = (1, 1, 0)$, since $1 = \exp(0)$.

If the centred point is good in scaled units but misses the raw explicit feasibility gate, an optional raw-feasibility repair step solves another conic problem in the same feasible set while penalising movement from the centred reference. A typical repair penalty is a one-norm movement proxy.

$$\min_{x,p,q}\ \omega_{\text{rep}}\, \mathbf{1}^T \cdot (p + q)$$

$$\text{subject to } x \in \mathcal{X}, \qquad x - x_{\text{ref}} \le p, \qquad x_{\text{ref}} - x \le q, \qquad p \ge 0, \quad q \ge 0.$$

Here $\omega_{\text{rep}} > 0$ is the repair penalty weight and $p, q \in \mathbb{R}^n$ are the nonnegative positive and negative parts of the movement from the centred reference x_{ref}.

Other start modes can be used as fallbacks: a direct feasible conic solve, an elastic feasible solve, a relaxed feasible solve, and a last-resort all-ones algebraic point. Irrespective of the start mode, acceptance is governed by the same raw explicit residual gate

$$r_{\text{exp}}(x^0) \leq \tau_{\text{start}}.$$

A scaled residual may be retained for diagnosis, but it does not by itself make a raw-infeasible point acceptable. The one relaxation of this gate is that a centred start whose raw residual exceeds τ_{start} is still admitted when the repair step strictly improved it and it meets a slightly larger soft allowance. This design ensures that the point entering the outer loop is feasible in the same row units used later by the explicit-bound step cap and by the line-search acceptance test.

F.8 Adaptive portfolio of inner conic models

At each major iteration k, the algorithm evaluates the current merit state at x^k and then chooses a strategy from an ordered portfolio. Each strategy builds an inner conic model whose feasible set is the working conic feasible set, possibly augmented by temporary local rows or convex epigraph variables. The inner objective is constructed from the current outer merit gradient, optional residual-balancing weights, and optional convex regularisation terms. The endpoint of the inner conic solve is not accepted directly; it defines a direction that must pass descent and line-search safeguards.

F.8.1 Cost approximation

The simplest strategy is cost approximation. It solves an inner conic problem of the form

$$\min_{x \in \mathcal{X}} (c^k_{\text{in}})^T \cdot x,$$

where $c^k_{\text{in}} \in \mathbb{R}^n$ equals the active part of the current merit gradient, with fixed and structurally inactive slots suppressed and with optional small inactive tie-break terms. The endpoint $x^k_{\text{in}} \in \mathbb{R}^n$ defines the candidate direction

$$d^k := x^k_{\text{in}} - x^k \in \mathbb{R}^n.$$

In an exact conic solve, if x^k is feasible for the same inner model and c^k_{in} is precisely the gradient model, the inner objective should not increase along this endpoint direction. A value of $\nabla\phi(x^k)^T \cdot d^k$ that is not sufficiently negative, specifically $\nabla\phi(x^k)^T \cdot d^k \geq -\tau_{\text{desc}}$ for a solver-tolerance-aware threshold $\tau_{\text{desc}} \geq 0$, is interpreted as a no-descent event and triggers diagnostics or a switch to a safer strategy, unless a curvature-rescue test on the finite-step second-order model admits the direction despite the failed first-order test.

F.8.2 Reachable-boundary cost approximation

The reachable-boundary cost approximation strategy augments the cost approximation model with a linear attraction toward a locally reachable exponential boundary. It first solves an auxiliary projection problem over the explicit feasible region to determine whether movement toward the local boundary is retained under the explicit constraints. Let $w^k \in \mathbb{R}^m_{\geq 0}$ be nonnegative weights selecting boundary-relevant residual components. With the gradient convention above, a boundary-attraction vector can be written as

$$a^k_{\text{bd}} := \nabla h(x^k) \cdot w^k \in \mathbb{R}^n.$$

The inner objective is then biased by the additional linear term

$$\eta^k_{\text{bd}} \, (a^k_{\text{bd}})^T \cdot x,$$

where the scalar η^k_{bd} is set to zero when the projection diagnostic indicates that useful boundary movement is not reachable. The purpose of this linear bias is not to introduce nonconvexity. Rather, it preserves conic representability while encouraging a direction whose first-order effect reduces the active boundary gap.

In addition to this linear attraction, the reachable-boundary strategy also appends a convex target-centred penalty on the selected blocks. For each such block it introduces a nonnegative epigraph variable $q_{\text{i}} \in \mathbb{R}_{\geq 0}$ together with the curvature-scaled deviations of $t_1(x)$ and $t_3(x)$ from a projected boundary target, collected in a vector $z_{\text{i}} \in \mathbb{R}^2$, imposes $(q_{\text{i}}, 1, z_{\text{i}})$ in a rotated quadratic cone (so that $\|z_{\text{i}}\|_2^2 \leq 2q_{\text{i}}$), and adds the term $\mu \sum_{\text{i}} w_{\text{i}} q_{\text{i}}$ to the inner objective, with the same nonnegative weights w_{i} and a penalty scale $\mu > 0$; the curvature scaling is frozen at x^k. This penalty is a convex second-order-cone term, so the inner model remains conic-representable, and it is inert when $\mu = 0$ or no block is selected.

F.8.3 Quadratic and local-box regularisation

When the first-order cost approximation becomes unreliable, the algorithm can add convex quadratic regularisation while keeping the inner model conic representable. Let $W_x \in \mathbb{R}^{n\times n}$ and $W_1, W_3 \in \mathbb{R}^{m\times m}$ be nonnegative diagonal weight matrices. A representative quadratically regularised model is

$$\min\ (c_{\text{in}}^k)^T \cdot x + \lambda_x \xi_x + \lambda_1 \xi_1 + \lambda_3 \xi_3$$

$$\text{subject to } x \in \mathcal{X},$$

$$\left\| W_x^{1/2} \cdot (x - x^k) \right\|_2^2 \le \xi_x,$$

$$\left\| W_1^{1/2} \cdot (t_1(x) - t_1(x^k)) \right\|_2^2 \le \xi_1,$$

$$\left\| W_3^{1/2} \cdot (t_3(x) - t_3(x^k)) \right\|_2^2 \le \xi_3.$$

Each squared-norm epigraph is represented by a rotated quadratic cone, for example by imposing $(\xi, 1, z)$ in a rotated quadratic cone so that $\|z\|_2^2 \le 2\xi$. In a local-box variant, temporary affine-image bounds are also added:

$$\left| t_1(x) - t_1(x^k) \right| \le \Delta_1^k, \qquad \left| t_3(x) - t_3(x^k) \right| \le \Delta_3^k.$$

These local rows are temporary. They stabilise the inner endpoint by restricting movement in the affine images that control the exponential boundary, but they do not redefine the global feasible set. The local widths may be adapted according to the observed agreement between predicted and actual merit decrease.

F.8.4 Block-curvature and residual-balanced variants

A block-curvature variant applies conic quadratic regularisation selectively to exponential-cone blocks whose curvature diagnostics at the current iterate indicate that the linear model is unreliable. This avoids adding unnecessary epigraph variables to all blocks while still damping the directions that dominate the mismatch between the linear model and the observed merit change. A residual-balanced variant rescales the h and g contributions to the merit gradient so that one residual family does not numerically dominate the inner objective solely because of scale.

F.8.5 Safeguarded gradient directions

If the conic endpoint models fail to provide a useful direction, the portfolio includes safeguarded gradient directions. The basic gradient direction is

$$d^k := -\nabla\phi(x^k) \oslash \max\{1, \left\|\nabla\phi(x^k)\right\|_\infty\},$$

with fixed and structural coordinates suppressed as required. A local-box gradient variant combines this direction with the same affine-image neighbourhood restrictions used by local-box cost-approximation strategies.

F.9 Step safeguards and line search

After a candidate direction d^k has been constructed, the algorithm searches along the ray

$$x(\lambda) := x^k + \lambda d^k, \qquad \lambda \ge 0.$$

The first admissible trial step is the minimum of several independent caps. The explicit-bound cap is the largest step satisfying

$$b_l - \tau_{\text{exp}}\mathbf{1} \le A \cdot x(\lambda) \le b_u + \tau_{\text{exp}}\mathbf{1}, \qquad l - \tau_{\text{exp}}\mathbf{1} \le x(\lambda) \le u + \tau_{\text{exp}}\mathbf{1},$$

where τ_{exp} is the accepted-trial explicit feasibility tolerance in the current working row units. A log-domain cap prevents t_1 from approaching the logarithmic singularity:

$$t_1(x(\lambda)) > \eta\mathbf{1}.$$

An exponential-boundary cap prevents a boundary-safe strategy from stepping across the reduced exponential graph. Along the trial ray the boundary gap is

$$h(x(\lambda)) = t_1(x(\lambda)) - \exp(t_3(x(\lambda))) \in \mathbb{R}^m.$$

The boundary cap is the first positive λ at which any component of $h(x(\lambda))$ reaches zero, multiplied by a fraction-to-boundary safety factor. Additional caps can limit predicted coefficient changes and numerically excessive affine-image movement. These caps are diagnostic as well as protective: the active cap identifies whether a failed or tiny step was caused by explicit feasibility, log-domain safety, exponential-boundary safety, coefficient damping, or numerical movement damping.

The primary line-search acceptance test is an Armijo condition on the outer merit:

$$\phi(x^k + \lambda d^k) \le \phi(x^k) + \sigma\lambda\nabla\phi(x^k)^T \cdot d^k, \qquad 0 < \sigma < 1.$$

A trial point must also satisfy the explicit feasibility gate and the log-domain gate. Once the maximum residual $\theta(x)$ is sufficiently small, the optional maximum-residual gate requires

$$\theta(x^k + \lambda d^k) \le \theta(x^k) - \delta_\theta + \tau_\theta,$$

where δ_θ is a required decrease and τ_θ is an absolute or relative numerical allowance. If a trial fails, λ is reduced geometrically and the tests are repeated. The accepted update is

$$x^{k+1} := x^k + \lambda_{\mathrm{k}} d^k.$$

For nominal no-descent directions, a curvature-rescue test may be applied before rejection. This test evaluates a finite-step second-order model

$$\Delta_2(\lambda) := \lambda\nabla\phi(x^k)^T \cdot d^k - \chi_{\mathrm{curv}}(\lambda d^k),$$

where χ_{curv} is a positive curvature correction estimated from the exponential and logarithmic nonlinearities. A direction that is not a strict first-order descent direction can still proceed to line search if the finite-step model and the actual merit decrease are both favourable.

F.10 Portfolio adaptation

The portfolio controller maintains an ordered list of strategies from simpler to safer or more regularised models. The principal strategies are cost approximation, reachable-boundary cost approximation, block-curvature regularised cost approximation, residual-balanced quadratically regularised cost approximation, quadratically regularised cost approximation, local-box quadratically regularised cost approximation, safeguarded gradient, and local-box safeguarded gradient. A user-supplied order may use all or a subset of these strategies. The default order omits the two safeguarded gradient strategies, which are enabled only on request.

Within each major iteration, if the current strategy fails to solve its inner conic problem, produces a nonfinite direction, produces a no-descent direction, or fails line search, or yields only a microscopic boundary-limited step, the controller advances to the next available strategy in the same major iteration. If all strategies have been attempted without an accepted step, the portfolio is exhausted. Conversely, after repeated calm accepted steps, the controller may probe back toward a simpler strategy, unless recent diagnostics indicate high curvature, repeated no-descent classifications, or a small-residual regime in which the simpler model is likely to repeat the previous failure mode.

The controller also uses diagnostic triggers. A stagnation detector advances to a safer strategy when the same accepted strategy produces too little relative merit decrease over a short window. A curvature detector switches from ordinary cost approximation to a curvature-aware strategy when recent accepted cost-approximation rows have a large curvature ratio or a repeated dominant curvature block. A boundary-saturation detector recognises regimes in which the model remains feasible but the step is repeatedly throttled by exponential-boundary caps.

F.11 Finalisation and returned certificate

The outer iteration returns an accepted primal point, but a downstream conic interface often requires a primal-dual tuple. The finalisation stage therefore separates three concepts: the last accepted outer primal point, the possibly polished returned primal point, and the dual certificate for the returned point. Let x_{acc} denote the last accepted outer iterate. The final accepted outer residual is

$$\theta_{\mathrm{acc}} := \theta(x_{\mathrm{acc}}).$$

If the finalisation policy permits polish, and either the residual is already below a polish threshold or polish is explicitly requested, an unrestricted conic polish solve is attempted using the caller-supplied objective a over the original unboxed working conic model. A polished point is accepted only if it passes the final explicit-feasibility

and outer-residual acceptance gates. If polish is not attempted, fails, or is rejected, an auxiliary fixed-primal recovery problem may be solved to recover dual variables at x_{acc} without moving the primal point.

The final returned point x_{fin} is audited in the same working row units used by the inner solves, and explicit residuals are also reported in the original input row units when row scaling was used. The final Karush-Kuhn-Tucker residual is evaluated on the actual returned primal-dual tuple, not merely on the auxiliary problem that produced it. The outer convergence status is determined from

$$\theta_{\mathrm{fin}} := \theta(x_{\mathrm{fin}}) = \max\left\{\|h(x_{\mathrm{fin}})\|_\infty, \|g(x_{\mathrm{fin}})\|_\infty\right\}.$$

If polish moves the primal point and brings θ_{fin} below the requested tolerance, the returned solution is classified as converged at the returned primal point even if the pre-polish portfolio stopped because all direction models were exhausted. This avoids conflating failure of the outer step generator with failure of the returned primal solution.

F.12 Diagnostics and interpretation

The algorithm records compact diagnostic rows for initial starts, accepted steps, rejected attempts, and finalisation. These diagnostics are not merely logging artefacts; they separate mathematically distinct failure modes. A solve failure means that the inner conic optimiser did not return a usable endpoint. A no-descent event means that the endpoint did not define a descent direction for the selected outer merit. A line-search failure means that a descent direction was found but no trial step satisfied the explicit feasibility, domain, boundary, merit, and maximum-residual gates. A microscopic boundary-limited step means that a formally acceptable step was so small, typically because of an exponential-boundary cap, that it was treated as no real progress.

No-descent diagnostics compare the tested directional derivative, the movement in the actual inner objective, explicit feasibility of the current and endpoint points, and the Karush-Kuhn-Tucker residual of the endpoint. This distinction is important because a no-descent row can arise from stationarity, complementarity, loose endpoint feasibility, objective scaling, masking of structural variables, or local-box constraints. Boundary-skating diagnostics identify active components for which h is already small and the first-order derivative points toward the exponential boundary, yielding a tiny fraction-to-boundary step. Curvature diagnostics estimate how much the nonlinear merit deviates from its first-order model and identify blocks that repeatedly dominate this deviation.

The diagnostic summaries are used to interpret the solve and to guide the adaptive portfolio. They do not replace the mathematical acceptance gates. The reported solution should therefore be interpreted through three simultaneous quantities: explicit primal feasibility, outer nonlinear residual, and the final primal-dual Karush-Kuhn-Tucker residual.

F.13 Convergence interpretation

The algorithm should be interpreted as a safeguarded sequential approximation approach rather than as a single monolithic conic optimisation problem. Each inner conic solve supplies a direction for a nonlinear boundary-matching merit; it does not by itself certify convergence of the outer problem. Conversely, a successful final polish can return a primal point satisfying the nonlinear residual tolerance even when the adaptive portfolio has exhausted its direction models. The most meaningful success criterion is therefore the final returned residual θ_{fin}, together with explicit primal feasibility and the final Karush-Kuhn-Tucker audit of the returned tuple.

The adaptive design is motivated by the mixed numerical regimes typical of large exponential-cone models. Far from the boundary, a simple cost approximation often gives useful progress. Near the boundary, the same first-order model may become curvature dominated or boundary throttled. The portfolio addresses this by adding reachable-boundary attraction, conic quadratic regularisation, local affine-image boxes, residual balancing, and safeguarded gradient steps only when diagnostics indicate that the simpler model is insufficient.
